# Living With *ALK* Fusion-Positive NSCLC: A French Nationwide Survey of the Psychosocial Impact Among Patients, Caregivers, and Health Care Professionals

**DOI:** 10.1016/j.jtocrr.2026.101038

**Published:** 2026-06-30

**Authors:** Jacques Cadranel, Romain Corre, Peggy Roget, Michael Wingler, Sandrine Alliaume, Pascale Bausson, Leslie Capdevielle, Joana Mendes, Alice Mogenet, Bertrand Mennecier

**Affiliations:** a Service d’Oncologie Thoracique et Pneumologie, Hôpital Tenon, Paris, France; b Service de Pneumologie, CH de Cornouaille, Quimper, France; c Service d’Oncologie Thoracique, CHU de Lille, Lille, France; d Patients en Reseau–Mon Réseau Cancer du Poumon, Paris, France; e Association ALK+ ROS1 Cancer Poumon France, Saint-Cyr-au-Mont-d’Or, France; f APLUSA (A+A), Boulogne-Billancourt, France; g Affaires Médicales, Takeda France, Paris, France; h Multidisciplinary Oncology and Therapeutic Innovations Department, Hôpitaux Universitaires de Marseille Nord Chemin des Bourrely, Marseille, France; i Department de Pneumologie, CHRU Brabois, Nancy, France

**Keywords:** Anaplastic lymphoma kinase, Caregivers, Disease burden, Non–small cell lung cancer, Patient-reported outcomes

## Abstract

**Introduction:**

The development of ALK inhibitors has transformed the management of *ALK* fusion-positive (*ALK+*) NSCLC from a high-mortality disease to a more manageable chronic condition.

**Methods:**

This quantitative, nationwide French survey collated information via online questionnaires from patients (*n* = 85), caregivers (*n* = 27), and health care professionals (HCPs; *n* = 50) on the impact of *ALK+* NSCLC on daily lives and psychosocial issues.

**Results:**

Overall, 46 patients (54%) had received first-line and 39 patients (46%) second-line or later targeted therapy for a mean of 2.4 years. The most common disease-related challenges reported by patients and HCPs were psychological issues (56.5% and 78.0%), life-planning difficulties (50.6% and 64.0%), and limitations in physical activities of daily life (40.0% and 48.0%), respectively. HCPs consistently underestimated the impact of *ALK+* NSCLC on patients’ daily lives, especially regarding psychological difficulties, issues with relationships and intimacy, and financial struggles. *ALK+* NSCLC had a major impact on caregivers’ psychological problems, difficulties in realizing life plans, and professional difficulties. The most important patient-rated support and guidance needs were to establish a separate clinic dedicated to nonmedical problems, improve the systematic provision of wellness activities (e.g., yoga), make it easier to report adverse events to the medical team, provide psychological support, and enable exchanges with other patients (e.g., via discussion groups).

**Conclusions:**

Our study highlights several measures that can be introduced to reduce disease burden and improve quality of life and, besides routine medical follow-up, markedly improve the overall patient journey through *ALK+* NSCLC.

## Introduction

*ALK* fusion-positive (*ALK*+) NSCLC accounts for 3% to 5% of all NSCLC cases.^1^, ^2^, ^3^ *ALK+* NSCLC typically affects younger individuals and is often associated with extensive disease, brain metastases, pleural effusion, and mediastinal lymph-node involvement at diagnosis.^4^, ^5^, ^6^ Historically, outcomes were poor, but the introduction of crizotinib, a first-generation ALK tyrosine kinase inhibitor (ALKi), substantially improved prognosis. However, crizotinib has poor central nervous system (CNS) penetration, limiting its efficacy in brain metastases, and provides a median progression-free survival (PFS) of only 10 to 11 months.^7^, ^8^, ^9^, ^10^

Second-generation (e.g., alectinib, brigatinib)^9^^,^^11^ and third-generation (e.g., lorlatinib)^12^ ALKis significantly enhance survival outcomes, such that ALKis are now the standard of care for advanced *ALK*+ NSCLC^10^^,^^13^^,^^14^ and have changed *ALK*+ NSCLC from a high-mortality condition to a more manageable, chronic disease.^15^

Based on 5-year data from the phase 3 CROWN study of lorlatinib versus crizotinib in patients with advanced *ALK*+ NSCLC not previously treated with an ALKi,^16^ lorlatinib is now prioritized as a first-line (1L) treatment for *ALK*+ NSCLC by some experts.^17^ Other 1L options include alectinib (ALEX study)^18^ and brigatinib (ALTA-1L study).^11^ However, these agents may have a detrimental impact on patient quality of life (QoL).^17^^,^^19^^,^^20^ For example, lorlatinib is often associated with cognitive dysfunction, weight gain, and peripheral neuropathy in addition to metabolic adverse events (AEs), which might be associated with long-term cardiovascular toxicity.^17^ Later-generation ALKis with improved tolerability profiles are being investigated, such as neladalkib (ALKAZAR study; NCT06765109); however, results are not expected until later this decade.

Given the long survival now associated with *ALK*+ NSCLC and the specific characteristics of patients, mostly young (median age at diagnosis is approximately 50 years), female nonsmokers or light smokers,^4^^,^^7^^,^^10^^,^^14^^,^^21^, ^22^, ^23^ people living with *ALK*+ NSCLC may face major ongoing psychosocial implications in their daily lives. Indeed, many patients may try to resume professional activities or sports participation, improve their ability to perform daily activities, and enhance their emotional and psychological well-being.^24^, ^25^, ^26^, ^27^, ^28^

From the perspective of health care providers (HCPs), it is important to provide relevant psychological and supportive care for patients and caregivers (e.g., via education programs, coordination nurses, psychologists, and support groups). Because *ALK*+ NSCLC disease management tends to focus primarily on the prevention and management of disease recurrence, the nonmedical needs of patients and caregivers may sometimes be overlooked.^25^^,^^29^^,^^30^ Oncologists alone are often not best suited to identify and address such nonmedical needs.^31^^,^^32^

The aim of this French nationwide survey was to collate and analyze detailed information from patients, caregivers, and HCPs (hospital oncologists and nurses) on the practical impact of *ALK*+ NSCLC and its treatment on the daily lives and future planning of patients and caregivers, the psychosocial issues faced by patients and caregivers, and the needs and expectations of patients and caregivers regarding supportive care.

## Materials and Methods

This French survey comprised two phases: a qualitative phase (Supplementary information and Supplementary Tables 1 and 2) and a quantitative phase. The qualitative phase (previously reported at the Congrès de Pneumologie de Langue Française [CPLF] in 2025^33^) was designed to provide an in-depth exploration of the impact of the disease and its management on patients’ daily lives, thereby offering a complementary perspective to the quantitative data collected and contributing to the refinement and completion of more focused and definitive questionnaires for patients, caregivers, and HCPs.

The quantitative phase involved gathering responses to the questionnaires to evaluate disease impact and its management through systematically collected quantifiable data to understand the perspectives of patients, caregivers, and HCPs. Patients were recruited from patient associations, the “ALK+ROS1” association and the association “PATIENTS EN RESEAU - Mon Réseau Cancer du Poumon®, Paris”, both based in France. A few patients and caregivers were recruited directly via physicians contacted by *A*PLUS*A* (A+A). All survey participants provided informed consent to participate in the study. This manuscript focuses on data from the quantitative phase only.

### Quantitative Phase

This study phase was conducted online, where patients, caregivers, and HCPs completed self-administered questionnaires. The patient questionnaire (Appendix A) and caregiver questionnaire (Appendix B) were each divided into four parts: (1) based on 1L or second-line (2L) or later targeted therapy, it addressed factors pertinent to the *ALK*+ NSCLC diagnosis; (2) addressed *ALK*+ NSCLC–targeted therapy (i.e., alectinib, brigatinib, and lorlatinib) and its perception; (3) assessed the impact of disease management on daily life, as measured by the visual analogue scale (VAS), with scores ranging from 0 (not affected at all) to 10 (very affected); and (4) collected sociodemographic information. The HCP questionnaire (Appendix C) comprised five parts focused on the following: (1) characteristics of patients with *ALK*+ NSCLC, which were consistent with the first part of the patient questionnaire; (2) accessible care within the institution; (3) announcements of the initial *ALK*+ NSCLC diagnosis and relapse; (4) the follow-up of patients receiving targeted therapy; and (5) perceived disease impact (measured by VAS) and the influence of disease management on patients’ daily lives.

### Statistical Analyses

The sample size for the quantitative analysis was chosen to ensure representativeness and sufficient precision while maintaining feasibility. All statistics were descriptive only, and all analyses were conducted using STAATS® statistical software (internal software developed by the company that conducted the survey). Quantitative variables were expressed as means, and qualitative variables were expressed as the number of individuals and percentages. Between-group comparisons were made using the Z-test and chi-square test. The significance threshold was defined as a *p* value less than 0.05. Inferential tests were conducted for exploratory purposes only and should be interpreted with caution due to the limited sample size.

## Results

### Patient, Caregiver, and HCP Characteristics

The quantitative phase was conducted between November 27, 2024, and February 20, 2025. The survey was completed by 85 patients with *ALK*+ NSCLC who were receiving targeted therapy, 27 family caregivers, and 50 thoracic or general medical oncologists who were treating patients with *ALK*+ NSCLC with targeted therapy. The enrolled patients were not necessarily cared for by the participating caregivers or oncologists.

### Patient Characteristics

Most of the patients were female (74.1%) and aged above or equal to 50 years (72.9%; Table 1). In most cases (52 patients; 61.2%), treatment was initiated at a university hospital or cancer center.

**Table 1:** Characteristics of Patients Surveyed: Quantitative Phase

Characteristic, *n* (%)	Patients (*n* = 85)
Sex	
Female	63 (74.1)
Male	22 (25.9)
Age	
<40 y	8 (9.4)
40–49 y	15 (17.6)
50–60 y	35 (41.2)
>60 y	27 (31.8)
Current household composition	
Couple, no children	40 (47.1)
Couple, with children	18 (21.2)
Single, no children	18 (21.2)
Single, with children	9 (10.6)
Geographic region	
North East	13 (15.3)
North West	19 (22.4)
South East	30 (35.3)
South West	10 (11.8)
Île-de-France	12 (14.1)
French overseas	1 (1.2)
Population size	
>100,000	20 (23.5)
50,000–99,999	10 (11.8)
10,000–49,999	21 (24.7)
2000–9999	21 (24.7)
<2000	13 (15.3)
Current treatment center	
University hospital	29 (34.1)
Cancer center	23 (27.1)
General hospital	23 (27.1)
Private hospital or clinic	7 (8.2)
Other	2 (2.4)
Unknown	1 (1.2)
Metastatic NSCLC^a^	
Yes	58 (68.2)
Cancer in one lung and other organs	42 (49.4)
Cancer in both lungs and other organs	16 (18.8)
No	27 (31.8)
Cancer in associated lymph nodes	13 (15.3)
Cancer in both lungs but not other organs	6 (7.1)
Do not know	8 (9.4)
Location of extrathoracic metastases (*n* = 58)^a^	
Brain	36 (62.1)
Bone	24 (41.4)
Liver	11 (19.0)
Other region	14 (24.1)
NOS	2 (3.4)
Treatment line	
1L	46 (54.1)
2L or later	39 (45.9)
Current targeted therapy with ALKi	
Marketed ALKi^b^	78 (91.8)
Alectinib	30 (35.3)
1L (*n* = 46)	21 (45.7)
2L (*n* = 39)	9 (23.1)
Brigatinib	24 (28.2)
1L (*n* = 46)	19 (41.3)
2L (*n* = 39)	5 (12.8)
Lorlatinib	26 (30.6)
1L (*n* = 46)	6 (13.0)
2L (*n* = 39)	20 (51.3)
Clinical trial^c^	8 (9.4)^d^

1L, first-line; 2L, second-line; ALKi, ALK inhibitor; NOS, not otherwise specified.

a Reported by patients during the survey. Patients who reported cancer in only one of their lungs and that no other organs were affected were excluded.

b Alectinib, brigatinib, lorlatinib.

c Administration of neladalkib (NVL-655) in the phase 1/2 ALKOVE-1 study (ClinicalTrials.gov identifier: NCT05384626).

d One patient received combination therapy with neladalkib plus a marketed ALKi.

Overall, 46 patients (54.1%) were receiving 1L targeted therapy, and 39 patients (45.9%) were receiving 2L or later targeted therapy. In almost all cases (91.8%), targeted therapy comprised alectinib, brigatinib, or lorlatinib. Alectinib (70.0% of cases) and brigatinib (79.2%) were administered predominantly in the 1L setting, and lorlatinib was used primarily in the 2L or later setting (76.9%). Significantly more patients in the 1L than in the 2L or later subgroup were treated with alectinib (45.6% versus 23.1%; *p* < 0.05) or brigatinib (41.3% versus 12.8%; *p* < 0.01), whereas the opposite was true for lorlatinib (13.0% versus 51.3%; *p* < 0.01). Eight patients in the 2L or later subgroup were receiving neladalkib (NVL-655) as part of the ongoing ALKOVE-1 clinical trial (NCT05384626).

Overall, patients in both subgroups had received targeted therapy for a mean of 2.4 years. Furthermore, 58 patients (68.2%) reported that they had metastatic NSCLC, with metastases involving the brain in almost two-thirds of the cases (62.1%). Brain metastases were significantly less frequent in the 1L subgroup than in the 2L or later subgroup (50.0% versus 76.9%; *p* < 0.05).

### Caregiver Characteristics

Caregivers were generally female (51.9%) and aged 50 to 60 years (44.4%); the overall mean age was 53.8 years. Most caregivers (85.2%) were the spouses of patients with *ALK*+ NSCLC; some caregivers (11.1%) were the children of patients. Caregivers most frequently reported that they provided moral and psychological support (100.0% of cases); helped with everyday tasks, such as housework and preparing meals (92.6%); accompanied patients on hospital visits (81.5%); talked to the treating medical team (70.4%); and searched for information about the illness and treatments (63.0%).

### HCP Characteristics

Altogether, 60.0% of the HCPs were thoracic oncologists and 40.0% were general medical oncologists. The mean duration of clinical practice was 15 years; 44.0% of the HCPs had been practicing for 6 to 15 years and 32.0% for 16 to 20 years. In total, 76.0% of the HCPs were practicing in university, general, or private nonprofit hospitals.

### Announcement of Diagnosis and Relapse

Specific consultations to announce the diagnosis of *ALK*+ NSCLC were not systematic. Overall, 70.6% of patients and 84.0% of HCPs reported having a specific consultation or system for diagnostic announcement; for the announcement of relapse, these proportions were 76.9% for patients in the 2L or later subgroup and 88.0% for HCPs. Moreover, some patients (24.7%) and oncologists (48.0%) reported having a systematic appointment with a nurse on the same day or a few days later to discuss the initial diagnosis of *ALK*+ NSCLC. For the announcement of relapse, the corresponding proportions were 15.4% for patients in the 2L or later subgroup and 40.0% for HCPs.

At initial diagnosis, patients reported being well looked after and supported by the medical team (52.9%) or by a relative or close friend (58.8%); at relapse, the corresponding values for patients in the 2L or later subgroup were 66.7% and 69.2%. However, at initial diagnosis, only 62.4% of patients overall reported receiving a sufficient level of information about the disease. At relapse, 84.6% of patients in the 2L or later subgroup reported receiving adequate disease information (*p* < 0.01 versus initial diagnosis). The corresponding proportions regarding the receipt of sufficient information about the implemented treatment were 74.1% at initial diagnosis and 84.6% at relapse.

Awareness of the AEs that might manifest during treatment was reported by some patients at initial diagnosis (40.0%) and in the 2L or later subgroup at relapse (43.6%). Although 42.0% of HCPs reported systematically providing patients with written support information or brochures about *ALK*+ NSCLC, only 21.2% of patients at initial diagnosis and 15.4% of patients at relapse reported receiving such information. Many patients at initial diagnosis versus those at relapse looked for information by themselves (85.9% versus 74.4%) or for information on the disease (81.2% versus 56.4%; *p* < 0.01), the implemented treatment (70.6% versus 64.1%), patient associations (54.1% versus 48.7%), and dedicated oncology support offered (51.8% versus 28.2%; *p* < 0.01). The main source of patient information was personal internet research (82.4% versus 66.7%).

### Follow-Up Consultations and Attitudes to Treatment

Overall, in response to the question “Which of the following professionals are currently following you in the context of your *ALK+* lung cancer?” patients reported that the most important roles during follow-up were held by oncologists (83.5%) or general practitioners or attending physicians (60.0%). More than two-thirds of patients (68.2%) considered family and friends to have an important role, and almost half of the patients (45.9%) thought patient associations had a major function.

Patients reported that during follow-up consultations, the psychological and emotional impact of the disease was addressed less systematically than other factors. Only 45.9% of patients reported that psychological and emotional factors were discussed “very often”, whereas computed tomography results (91.8%), treatment efficacy (76.5%), physical symptoms (71.8%), and treatment tolerability (67.1%) were discussed very often. Significantly fewer patients in the 1L versus 2L or later subgroup (22.4% versus 46.2%; *p* < 0.05) considered that the disease’s impact on daily life was discussed very often during follow-up. Almost two-thirds of HCPs (62.0%) reported that the impact of the disease on patients’ relationships and sex lives was rarely or never discussed during follow-up.

Overall, 97.6% of patients considered that they were very knowledgeable or fairly knowledgeable about their current targeted therapy, although only 28.2% received relevant information from non-oncologist sources, such as therapeutic education sessions, information workshops, or hospital pharmacists. HCPs reported that information services available (and used systematically or by most patients) within their institutions included coordination nurses (68.0% of HCPs), phone support arranged by a nurse or medical secretary (58.0%), medication review by a pharmacist (54.0%), and advanced practice nurses (38.0%). The use of phone support to monitor current treatment was reported by significantly more patients in the 1L than in the 2L or later setting (50.0% versus 17.9%; *p* < 0.01). Phone support helped patients better understand their treatment (66.7%) and manage AEs more effectively (70.0%). Of the 55 patients without phone support, 40 (72.7%) expressed that they would like to receive such support.

On a rating scale of 0 to 10, patients and HCPs consistently rated targeted therapy more favorably than chemotherapy regarding the following factors: ease of administration, benefits versus limitations, provision of hope, ability to lead an almost-normal life, preserved quality of life, long-lasting efficacy, and tolerable AEs (Supplementary Fig. 1).

### Impact of Disease and Treatment on Patients’ Daily Lives

HCPs consistently underestimated the impact of *ALK*+ NSCLC on patients’ daily lives, particularly regarding their psychological difficulties, problems with relationships and intimacy, and financial difficulties (Table 2).

**Table 2:** Disease Impact on Patients’ Daily Lives, as Reported by Patients and Health Care Professionals

Affected by the Following Difficulties, *n* (%):	Patients (*n* = 85)	HCPs (*n* = 50)^a^	*p* Value
Psychological	70 (82.4)	34 (68.0)	NS
Realizing life plans, future planning	69 (81.2)	37 (74.0)	NS
Physical difficulties in performing everyday tasks	63 (74.1)	–	–
Relationships and intimacy	56 (65.9)	15 (30.0)	<0.01
Professional	53 (62.4)	29 (58.0)	NS
Social life (family, friends)	52 (61.2)	–	–
Financial	52 (61.2)	19 (38.0)	<0.01

HCPs, health care professionals; NS, not significant.

a HCPs reporting that most or all of their patients were affected by the listed difficulties.

Overall, 39 of 85 patients (45.9%) had experienced AEs to targeted therapy in the previous month. Among these patients, nine (23.1%) reported that the AEs severely limited their daily activities. Conversely, 35 of 50 HCPs (70.0%) reported that only a few patients had AEs to targeted therapy that severely limited their daily activities.

The top-ranked disease-related difficulties by patients and HCPs, respectively, were psychological issues (56.5% and 78.0%), problems with life plans (50.6% and 64.0%), and problems with physical activities of daily life (40.0% and 48.0%) (Fig. 1). The most frequently reported psychological difficulties were insecurity and fear of the future (49.4% of patients), feelings of anxiety (48.2%), feelings of anger and injustice (42.4%), and mood disorders (37.6%) (Fig. 2). Feelings of anger and injustice were reported significantly less often in the 1L subgroup than in the 2L or later subgroup (32.6% versus 53.8%; *p* < 0.05). Altogether, more HCPs than patients (36.7% versus 18.8%; *p* < 0.05) reported that psychological difficulties had a major impact on patients daily lives. In total, 70 of 85 patients (82.4%) reported experiencing at least one psychological difficulty, with 46 of these patients (65.7%) discussing this with a continuity of care HCP; this was rated as very helpful in 48.6% of cases.

**Figure 1:**
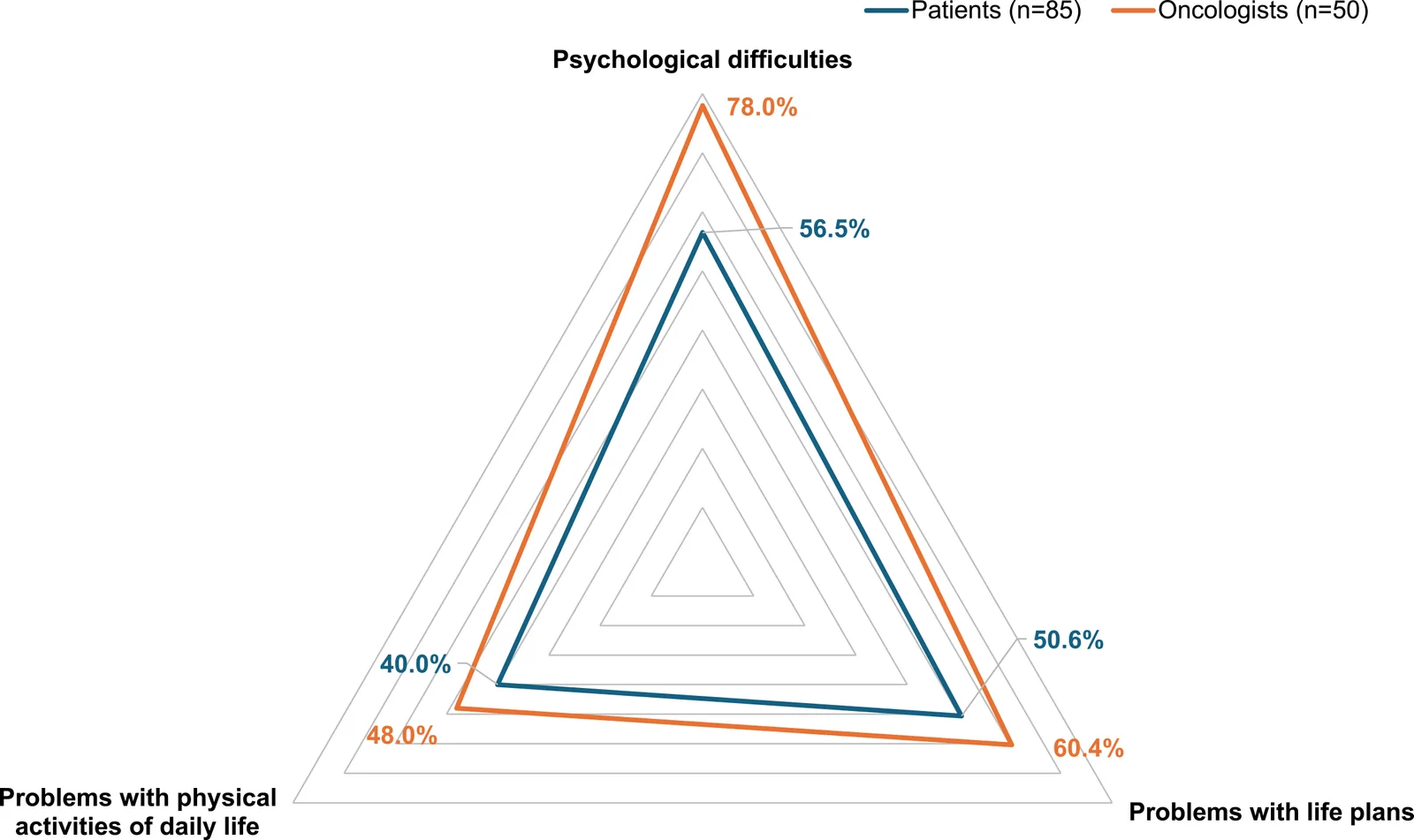
The top-ranked, disease-related difficulties by patients and oncologists.∗ In response to the following statement: Of all the difficulties caused by the disease, please select the three that most affect your daily life (patient questionnaire) or the daily lives of most of your patients (oncologist questionnaire).

**Figure 2:**
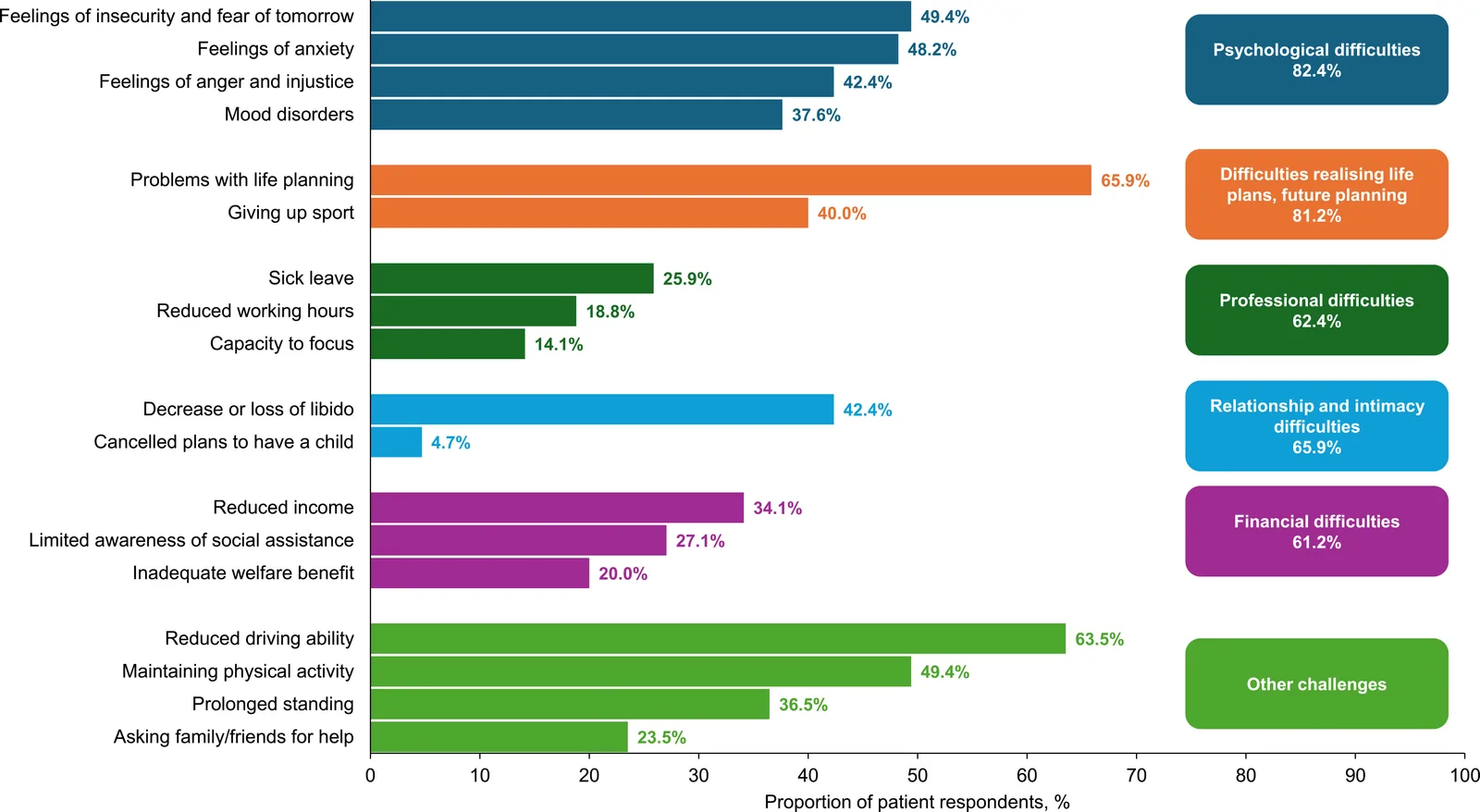
The most frequent patient-reported daily-life difficulties.∗ In response to the following statement: Of all the difficulties caused by the disease, please select the three that most affect your daily life (patient questionnaire).

Patients cited the most frequent problems with life projects as follows: “I find it hard to project myself into the future” (65.9%) and “I have had to give up certain sporting activities” (40.0%). More HCPs than patients (46.7% versus 25.9%; *p* < 0.05) reported that problems with life projects had a major impact (VAS score 8–10) on patients’ daily lives.

The most frequent physical difficulties listed by patients were as follows: “It is difficult for me to maintain a physical and/or sporting activity” (49.4%) and “It is hard for me to stay on my feet for too long” (36.5%); the latter point was expressed significantly less often by patients in the 1L subgroup than by those in the 2L or later subgroup (21.7% versus 53.8%; *p* < 0.01). Importantly, 44.7% of patients reported that they received assistance with domestic tasks from a relative, and 14.1% reported that they received such assistance from a home care worker. In total, 63 of 85 patients (74.1%) reported discussing physical difficulties with a continuity of care HCP, which patients rated as very helpful in 44.4% of cases.

Other disease-related difficulties reported by patients included a decrease in or loss of libido (42.4%) and having to stop work (25.9%; Supplementary Table 3).

### Impact of Disease and Treatment on Caregivers’ Daily Lives

Several caregivers reported fatigue (77.8%), anxiety (70.4%), sleep disorders (59.3%), and organizational difficulties or burnout (33.3%) associated with their caregiving role. Overall, 24 of 27 caregivers (88.9%) lived with the patient. Considerably more caregivers than patients rated *ALK*+ NSCLC as having a major impact on certain aspects of their daily life: psychological difficulties (100.0% of caregivers versus 82.4% of patients); difficulties in realizing life plans or planning for the future (88.9% versus 81.2%); and professional difficulties (66.7% versus 62.4%).

Regarding psychological difficulties, 70.4% of caregivers experienced anxiety, 66.7% experienced episodes of sadness, and 51.9% experienced feelings of insecurity. Only 37.0% of caregivers had discussed their psychological difficulties with a continuity of care HCP, and only 22.2% of caregivers had found these discussions to be very helpful.

In terms of life planning, caregivers reported difficulties with projecting future plans (59.3%); having to give up organizing trips and holiday plans (51.9%); and difficulties in setting up projects and planning (48.1%). However, 75.0% of caregivers were not interested in discussing these difficulties with a continuity of care HCP.

In total, 29.6% of caregivers expressed difficulties fulfilling professional obligations; 14.8% reported having to rearrange working hours because of their relative’s illness; and 14.8% had to change professions. However, 70.6% of caregivers were not interested in discussing these difficulties with a continuity of care HCP.

### Supportive Care Needs and Expectations

The most important support and guidance needed by patients (versus HCPs) to help patients deal with the challenges of *ALK*+ NSCLC were to establish a separate hospital consultation, dedicated to nonmedical problems, for patients and caregivers (65.9% of patients versus 58.0% of HCPs); other important needs are found in Figure 3.

**Figure 3:**
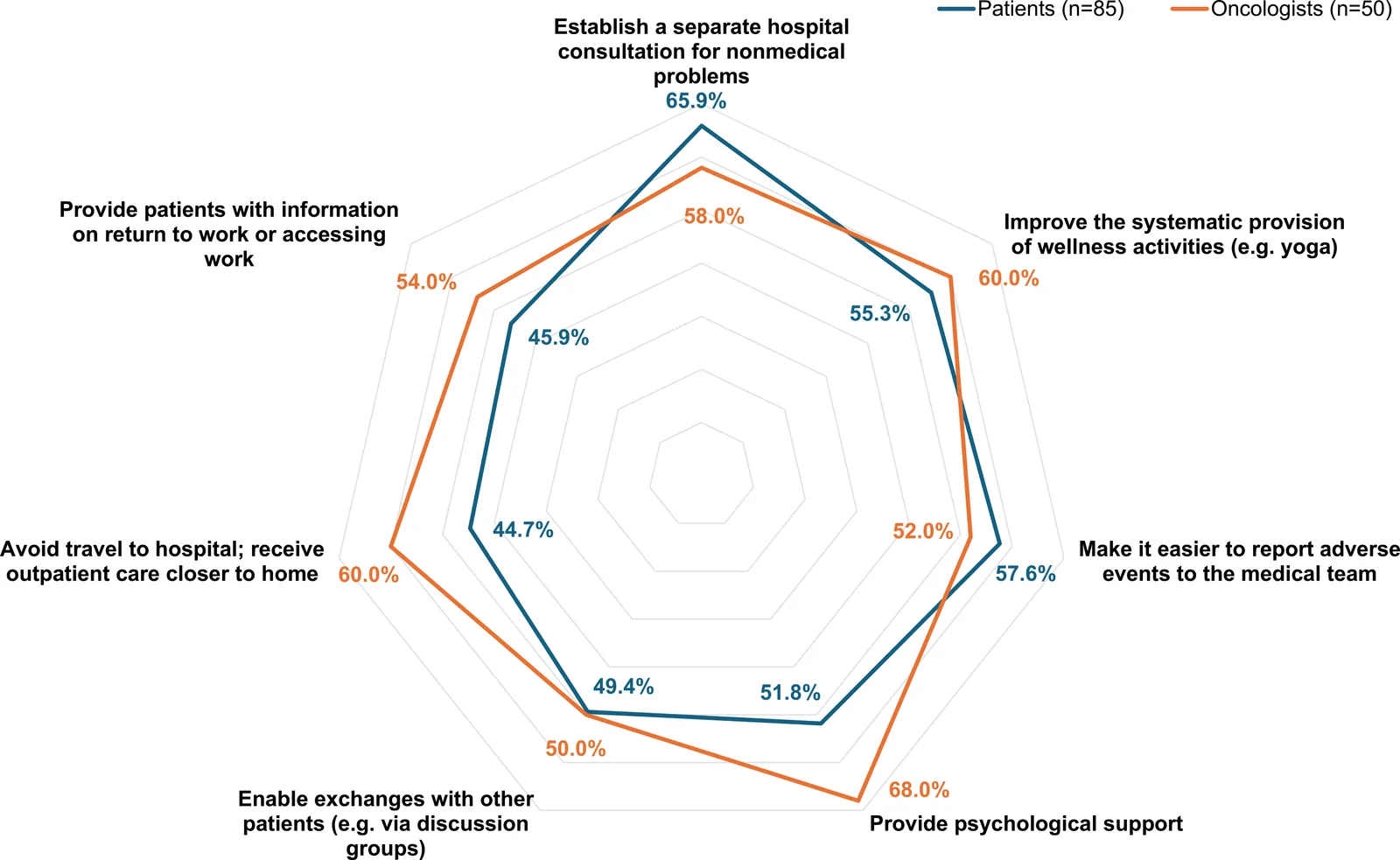
The top-ranked support and guidance needs rated by patients and oncologists.∗ In response to the following statement: Here are several actions/initiatives that could be put in place to support patients with *ALK+* cancer (undergoing targeted therapy); how important do you think these are?

Caregivers considered the most important support and guidance measures that they required were: increased information on the disease and its progression (74.1%); increased information on caregiver rights (59.3%); improved tools and support for self-training as caregivers (59.3%); the availability of a separate hospital consultation dedicated to nonmedical problems for patients and caregivers (59.3%); and assistance with learning how to better communicate with individuals who are unwell (48.1%).

## Discussion

Our survey results clearly underscore the frequent and diverse long-term burden outside of the usual AEs after drug use that is typically reported in *ALK*+ NSCLC trials for patients and caregivers, and the discrepancies that exist between patient and HCP perspectives on the disease impact on patients’ daily lives. The top-ranked disease-related difficulties reported by patients and HCPs were problems with psychological health, life plans, and physical activities of daily life.

Patients who participated in the quantitative phase of the study received targeted therapy with alectinib, brigatinib, or lorlatinib for a mean of 2.4 years. At initial diagnosis, many patients (30% to 40%) felt that they received insufficient information on their disease and its treatment, and approximately one-quarter of patients in the 2L or later subgroup reported receiving no specific consultation for the announcement of relapse, thus reflecting potentially limited health care resources in some regions. Fewer than two-thirds of patients considered the duration of follow-up consultations adequate, and fewer than half of patients thought that the psychological and emotional impact of the disease was addressed adequately during follow-up.

Thus, from the patient perspective, one of the main areas to address to improve patient supportive care and QoL is the better provision, at initial diagnosis, of information on the disease, suggested treatment, patient associations, and dedicated oncology support available. Psychological and emotional factors should be more rigorously addressed, as should be the general impact of *ALK*+ NSCLC on daily life. Indeed, in the 1L setting, almost 80% of patients reported that the disease impact was not adequately discussed during follow-up; in addition, half of the patients in the 1L subgroup reported that they did not receive relevant phone support to monitor the treatment they were receiving. Assistance in other areas, such as receiving physical help from a professional carer or support in returning to work and sporting activities, would also benefit many patients; for instance, half of the patients surveyed reported difficulty in maintaining a physical or sporting activity, and one-quarter reported having to give up work.

For caregivers, improved support is needed around psychological difficulties, which were reported by all caregivers surveyed; planning for the future (problematic for approximately 90% of caregivers); and addressing professional difficulties (reported by two-thirds of caregivers). Most caregivers reported fatigue, anxiety, and sleep disorders associated with their caregiving role; however, fewer than half of the caregivers had discussed psychological difficulties with a continuity of care HCP, and caregivers generally did not want to discuss project and life-planning issues or professional difficulties with a continuity of care HCP.

HCP perspectives revealed that HCPs consistently underestimated the impact of *ALK*+ NSCLC on patients’ daily lives. Nonetheless, areas for improvement suggested by 58% to 74% of HCP responses were the need to more thoroughly address patients’ problems in realizing life plans, psychological problems, and professional difficulties.

The study identified several common needs among patients, caregivers, and HCPs regarding potential improvements in care factors. For example, the need to provide a distinct consultation to help patients and caregivers address nonmedical problems and the need to systematically enhance access to general wellness activities, such as yoga and meditation. Other important areas identified for potential improvement were as follows: easier reporting of AEs to the medical team, as requested by patients; the provision of psychological support to patients, as expressed by more than two-thirds of HCPs; and increased information on *ALK*+ NSCLC and its progression, as requested by almost three-quarters of caregivers.

Overall, our findings are supported by data from other recent, real-world studies. The distribution of 1L and 2L ALKis in our study, as in the study by Bauman et al.,^34^^,^^35^ reflects both the availability of ALKis in France at that time—only alectinib and brigatinib were available initially, followed by lorlatinib—and the practice of sequential therapy and the predominance of lorlatinib in 2L. Thus, in our cohort, 73.9% of patients received alectinib or brigatinib as 1L treatment (100% in the Bauman study, and almost exclusively alectinib),^34^ and 51% received lorlatinib as 2L treatment (56% in the Bauman study).^34^ Similarly, the efficacy of ALKis observed in our cohort was similar to that described in other real-life cohorts,^34^, ^35^, ^36^, ^37^ as was the relatively short duration of efficacy among patients who received sequential ALKi therapy,^34^^,^^35^ possibly reflecting the poor prognosis of these patients developing early ALKi resistance.

However, lorlatinib is likely now the first-choice 1L therapy for many oncologists and patients, although data on overall survival, the potentially detrimental effects on long-term tolerability, and QoL will require close monitoring.^17^ At 5 years, grade 3/4 weight increase affected social life in 23% of patients due to lorlatinib (versus 2% with crizotinib) in the CROWN study; CNS AEs were experienced by 42% of the cohort, 86% of which were grade 1/2.^16^ Indeed, grade 1/2 CNS AEs were found to have a negative impact on QoL by interfering with work performance and limiting important daily activities.^37^ In addition, in line with our study findings, several other studies highlight factors such as the detrimental impact of NSCLC itself, with or without brain metastases, on QoL for patients and caregivers; the association with psychological problems; and relevant supportive measures that could be implemented for patients and caregivers.^29^^,^^36^^,^^38^

In other cancers with oncogenic drivers, such as breast cancer and chronic myeloid leukemia, various targeted therapies have also demonstrated effectiveness in real-world settings.^39^^,^^40^ The importance of tailored supportive care strategies, such as health education, lifestyle modification, physical activity, and psychological intervention, has also been emphasized in these scenarios.^40^^,^^41^

Some potential study limitations should be acknowledged. Although this is one of the largest studies of its type conducted to date in patients with *ALK*+ NSCLC, the relatively small sample size in each group (patients, caregivers, HCPs) may require that data extrapolation to some larger metropolitan populations or resource-poor regions be conducted with caution. Another potential study limitation was that fewer than one-third of patients (27/85 [31.8%]) were linked to the participating HCPs, thus possibly making some comparisons of disease and treatment perspectives and societal implications inaccurate. In addition, considerably more patients (74.1%) than caregivers (51.9%) were female, which may have introduced bias regarding participant perspectives on *ALK*+ NSCLC. Another bias may have been due to participants being recruited from patient association groups being more informed about their disease than those who do not belong to such groups; this may skew the perceptions of patient and caregiver knowledge of *ALK*+ NSCLC. Other possible sources of bias could not be avoided (e.g., the recruitment of participants via different sources in the qualitative phase; varying proportions of patients with 1L versus 2L or later treatment or brain metastases; variability in oncologist selection of 1L ALKi therapy; geographic variability based on metropolitan versus rural regions). Although participant recruitment via different sources and regions could be construed as a possible source of bias, data from diverse populations and care settings can also be regarded as a study strength.

Altogether, our study highlights various relevant measures that can be introduced to best address the many concerns of patients, caregivers, and HCPs to reduce the burden of disease and improve QoL for patients with *ALK*+ NSCLC. Indeed, implementation of some of these measures (e.g., widening access to supportive care and health care resources to include outpatient facilities closer to patients’ homes, so that patients’ travel times are reduced; facilitating AE reporting to medical teams; encouraging HCPs to focus more keenly on patient QoL; and actively addressing nonmedical issues) may allow more patients and caregivers to continue to work or return to work.

In conclusion, this nationwide survey of patients with *ALK*+ NSCLC, caregivers, and HCPs in France identified important areas for care optimization. These included medical aspects, such as initial disease announcement, patient education, and ongoing access to the medical team, and nonmedical issues, such as the need for improved psychological support for patients and caregivers. Systematic strategies targeting these care-optimization areas may substantially reduce the impact of *ALK*+ NSCLC on patients’ daily lives and enhance their overall QoL.

## CRediT Authorship Contribution Statement

Emma Lalaquit Gallant:

**Jacques Cadranel:** Conceptualization, Formal analysis, Investigation, Methodology, Validation, Visualization, Writing – review & editing.

**Romain Corre:** Conceptualization, Data curation, Investigation, Methodology, Validation, Visualization, Writing – review & editing.

**Peggy Roget:** Conceptualization, Methodology, Writing – review & editing.

**Mikael Wingler:** Conceptualization, Methodology, Resources, Writing – review & editing.

**Sandrine Alliaume:** Conceptualization, Methodology, Resources, Writing – review & editing.

**Pascale Bausson:** Conceptualization, Data curation, Formal analysis, Investigation, Methodology, Project administration, Resources, Supervision, Validation, Visualization, Writing – review & editing.

**Leslie Capdevielle:** Conceptualization, Funding acquisition, Methodology, Project administration, Supervision, Writing – review & editing.

**Joana Mendes:** Conceptualization, Funding acquisition, Methodology, Project administration, Supervision, Writing – review & editing.

**Alice Mogenet:** Conceptualization, Data curation, Investigation, Methodology, Project administration, Supervision, Validation, Visualization, Writing – review & editing.

**Bertrand Mennecier:** Conceptualization, Formal analysis, Investigation, Methodology, Validation, Visualization, Writing – review & editing.

## Data Availability

The data that support the findings of this study are available from the corresponding author on reasonable request.

## Ethics Approval and Consent to Participate

All survey participants provided informed consent to participate in this study, which was noninterventional and, therefore, did not require review and approval by the Comité de Protection des Personnes. All personal data processed in the study were stored on secure central computer servers in pseudonymized form (unless legally necessary to keep directly identified personal data, e.g., for payment purposes) and were held for 3 years after study completion. The data were processed, which included, without limitation, their collection and storage, in accordance with French Law number 78–17 of January 6, 1978, on computers, files and liberties (“Loi relative à l'informatique, aux fichiers et aux libertés”, also known as “LIL”) and Regulation (EU) 2016/679 of the European Parliament and the Council of April 27, 2016, on the protection of natural persons with regard to the processing of personal data and the free movement of such data (“GDPR”).

## Disclosure

Dr. Cadranel has received travel expenses from Pfizer, Takeda, and J&J and fees as a consultant and for participating on the board of experts from Amgen, AstraZeneca, Daiichi, J&J, MSD, Pfizer, Roche, and Takeda. Ms. Roget has received fees from Takeda and Roche for participating as board of experts or a consultant. Mr. Wingler and Mrs. Alliaume are representatives of patient associations. Mrs. Bausson is an employee of *A*PLUSA (A+A). Mrs. Capdevielle and Mrs. Mendes are employees of Takeda. Dr. Mogenet has received travel expenses from BMS and Takeda and honoraria and speaker fees from AstraZeneca, Pfizer, Takeda, Viatris, and Amgen. Dr. Mennecier has received fees from Takeda, Roche, and Pfizer for participating as board of experts or a consultant. The remaining author declare no conflict of interest.
