# Supplementary material for: Living With ALK Fusion-Positive NSCLC: A French Nationwide Survey of the Psychosocial Impact Among Patients, Caregivers, and Health Care Professionals
Source: JTO Clin Res Rep. 2026 Jun 30;7(10):101038. doi: 10.1016/j.jtocrr.2026.101038 (PMC13626941; doi:10.1016/j.jtocrr.2026.101038)
Supplement: Supplementary file 1 [file mmc1.docx]

**Living with ALK fusion-positive NSCLC: a French nationwide survey of the psychosocial impact among patients, caregivers and healthcare professionals**

**Authors:** Jacques Cadranel, Romain Corre, Peggy Roget, Mikael Wingler, Sandrine Alliaume, Pascale Bausson, Leslie Capdevielle, Joana Mendes, Alice Mogenet, Bertrand Mennecier

**Corresponding author:** Jacques Cadranel, Service d’Oncologie Thoracique et Pneumologie, Hôpital Tenon, 4 Rue de la Chine, 75970, Paris, France. Email: jacques.cadranel@aphp.fr

# Supplementary data

# Supplementary methods

## Qualitative phase

This phase was conducted between 10 June 2024 and 12 July 2024 and involved: 10 patients with locally advanced or metastatic ALK fusion-positive (*ALK*+) non-small cell lung cancer (NSCLC) receiving first-line (1L) targeted therapy (n=5) or second-line (2L) or later targeted therapy (n=5); three caregivers; and nine healthcare professionals (HCPs; seven thoracic or medical oncologists and two nurses) treating patients with *ALK*+ NSCLC. The 10 patients were selected through the *ALK*+ *ROS1* and *My Lung Cancer Network* patients’ associations (n=5), or directly by physicians (n=5). Time since the start of targeted therapy was >1 year for 80% of patients in the 1L subgroup and ≥2 years for all patients in the 2L or later subgroup (Table S1). Only 30% of patients were in full-time or part-time employment. Caregivers were recruited through the previously mentioned patients’ associations, and HCPs were recruited by the market research company (*A*PLUS*A*) conducting the study. The HCPs had diverse clinical experience (Table S2).

The qualitative survey comprised interviews conducted with patients, caregivers and HCPs by phone or videoconference with two trained moderators per interviewee using interview guides. The interview duration was 75 minutes for patients and caregivers and 60 minutes for HCPs. The interview guide for patient/caregiver interviews was constructed in four parts: 1) approach and knowledge of *ALK*+ NSCLC; 2) impact of the disease and treatment on patient quality of life (QoL), focusing on several factors, such as daily life, family and social interaction, leisure, physical, professional and psychological aspects, and financial issues; 3) disease management since diagnosis; and 4) relationship with the medical team. The interview guide for HCP interviews was constructed in three parts: principal stages of the patient journey through advanced *ALK*+ NSCLC; impact of the disease and treatment on patient QoL; and addressing societal issues associated with *ALK*+ NSCLC. No standardized measurement scales were applied; instead, open-ended questions were used to encourage participants to share their experiences and perspectives in their own words.

The sample size for the qualitative analysis was chosen as being sufficient to ensure a diversity of profiles to cover the widest possible range of attitudes towards *ALK*+ NSCLC and its management.

# Supplementary tables

## Table S1

Characteristics of patients and caregivers surveyed: qualitative phase

| **Characteristic, n** | **Patients (n=10)** | **Caregivers (n=3)** |
| --- | --- | --- |
| *Age* |  |  |
| <35 years | 3 | 0 |
| 35–60 years | 3 | 3 |
| >60 years | 4 | 0 |
| *Sex* |  |  |
| Female | 8 | 2 |
| Male | 2 | 1 |
| *Treatment line* |  |  |
| 1L | 5 | 2^a^ |
| 2L or later | 5 | 1^a^ |
| *Time since start of first targeted therapy for patients in the 1L subgroup* | | |
| 1 year | 1 | 0 |
| 1–2 years | 3 | 0 |
| >2 years | 1 | 2^a^ |
| *Time since start of first targeted therapy for patients in the 2L or later* *subgroup* | | |
| 2 years | 3 | 0 |
| >2 years | 2 | 1^a^ |
| *Marital status* |  |  |
| Single | 1 | 0 |
| Married, in a relationship | 8 | 3 |
| Separated, divorced, widowed | 4 | 0 |
| *Dependent child(ren)* |  |  |
| Yes | 1 | 1 |
| No | 9 | 2 |
| *Employment status* |  |  |
| Full-time | 2 | 0 |
| Part-time | 1 | 2 |
| On sick leave | 1 | 1 |
| Retired | 4 | 0 |
| Not working | 2 | 0 |
| *Place of residence, population size* |  |  |
| Île-de-France | 2 | 0 |
| >100,000 | 2 | 1 |
| 50,000–99,000 | 1 | 0 |
| 10,000–49,999 | 1 | 1 |
| 2000–9999 | 3 | 0 |
| <2000 | 1 | 1 |
| *Socio-professional category* |  |  |
| Unemployed | 1 | 0 |
| Manual worker | 0 | 1 |
| Employee | 3 | 1 |
| Mid-level executive | 3 | 1 |
| Senior executive | 2 | 0 |
| Self-employed professional | 1 | 0 |

^a^Responses from caregivers regarding a relative with *ALK+* NSCLC.

*Abbreviations:* 1L, first line; 2L, second line; ALK, anaplastic lymphoma kinase; NSCLC, non-small cell lung cancer.

## Table S2

Characteristics of healthcare professionals surveyed: qualitative phase

| **Characteristic, n** | **HCPs (n=9)** |
| --- | --- |
| *Specialty* |  |
| General medical oncologist | 4 |
| Thoracic oncologist | 3 |
| Nurse | 2 |
| *Sex* |  |
| Female | 5 |
| Male | 4 |
| *Age* |  |
| <45 years | 5 |
| ≥45 years | 4 |
| *Place of practice* |  |
| General hospital | 4 |
| Academic hospital | 3 |
| Cancer centre | 2 |
| *Place of residence* |  |
| Île-de-France | 4 |
| Auvergne-Rhône-Alpes | 2 |
| Occitanie | 2 |
| Nouvelle-Aquitaine | 1 |
| *Number of patients being treated with ALK+ NSCLC* |  |
| <10 | 4 |
| 10–20 | 3 |
| >20 | 2 |

*Abbreviations:* ALK, anaplastic lymphoma kinase; HCPs, healthcare professionals; NSCLC, non-small cell lung cancer.

## Table S3

Additional disease-related difficulties reported by patients (n=85)

| **Difficulty, n (%)** | **Patients (n=85)** |
| --- | --- |
| Decrease or loss of libido | 36 (42.4) |
| Having to stop work | 22 (25.9) |
| 1L subgroup | 16 (34.8)^a^ |
| 2L or later subgroup | 6 (15.4) |
| Having to adjust working hours | 18 (21.2) |
| Asking family or friends for help | 20 (23.5) |
| 1L subgroup | 7 (15.2)^a^ |
| 2L or later subgroup | 13 (33.3) |
| Cancelling plans to have a child | 5 (5.9) |
| Impaired ability to drive | 47 (62.7)^b^ |
| Reduced income | 29 (34.1) |
| Lack of information on social assistance schemes | 23 (27.1) |
| Inadequate welfare benefits | 17 (20.0) |

^a^p<0.05 vs ≥2L or later subgroup.

^b^Of 75 patients who were drivers or who had driven previously.

*Abbreviations:* 1L, first line; 2L, second line.

# Supplementary figures

## Fig. 1

(A) Patient and (B) oncologist ratings of targeted therapy versus chemotherapy (rating scale 0–10). AEs, adverse events; QoL, quality of life

Patients (n=85)

(A)

Mean rating scale score

|  | Targeted therapy | Chemotherapy |
| --- | --- | --- |
| Ease of treatment administration | 9.4 | 3.64815 |
| Favourable balance between treatment benefits and constraints | 8.7 | 5.2037 |
| Treatment that gives hope | 8.6 | 4.85455 |
| Treatment that allows you to live an almost normal life | 8.4 | 3.85714 |
| QoL preserved with treatment | 8.1 | 3.83929 |
| Treatment with long-lasting efficacy | 7.4 | 4.89362 |
| Tolerable AEs | 7.3 | 3.96364 |
| Treatment well monitored by medical team | 8.5 | 7.80769 |

Oncologists (n=50)

(B)

Mean rating scale score

|  | Targeted therapy | Chemotherapy |
| --- | --- | --- |
| Ease of treatment administration | 8.56 | 4.62 |
| Favourable balance between treatment benefits and constraints | 8.12 | 5.1 |
| Treatment that gives hope | 8 | 4.74 |
| Treatment that allows you to live an almost normal life | 7.98 | 4.24 |
| QoL preserved with treatment | 7.96 | 4.26 |
| Treatment with long-lasting efficacy | 7.92 | 4.84 |
| Tolerable AEs | 7.84 | 4.46 |
| Treatment well monitored by medical team | 7.67347 | 7.59184 |
