# Supplementary material for: Living With ALK Fusion-Positive NSCLC: A French Nationwide Survey of the Psychosocial Impact Among Patients, Caregivers, and Health Care Professionals
Source: JTO Clin Res Rep. 2026 Jun 30;7(10):101038. doi: 10.1016/j.jtocrr.2026.101038 (PMC13626941; doi:10.1016/j.jtocrr.2026.101038)
Supplement: Supplementary file 2 [file mmc2.docx]

**Appendix A**

**National Survey of Patients and Their Caregivers:**

**ALK+ Lung Cancer – Experience, Needs and Expectations of this Disease**

**PATIENT QUESTIONNAIRE [do not display – code 1 in S0]**

**Part A. [if patient in L1] The diagnosis of ALK+ lung cancer (3-5')/**

**[if patient in L2 and +] The initial diagnosis of ALK+ lung cancer and relapse [4-6']**

**Patients in L1** [= code 1 in S6 or code 2 in S6C] [do not display]

**Let's start with diagnosing your ALK+ lung cancer.**

**Q1.** How old were you when you were diagnosed with ALK+ lung cancer?

/__/__/Years

**Q2.** Was he diagnosed **in the same establishment** in which you are currently being followed?

*Only one answer possible*

| 1 | Yes | 🞊 |
| --- | --- | --- |
| 2 | No | 🞊 |
| 3 | I don't know | 🞊 |

**Q2B.** [if not in Q2] In which **school profile** was he diagnosed?

*Only one answer possible*

| 1 | University Hospital Center (CHU) / Regional University Hospital Center (CHUR) | 🞊 |
| --- | --- | --- |
| 2 | Anticancer Center / Center for the Fight Against Cancer (CLCC) | 🞊 |
| 3 | General Hospital; public health care establishment other than university hospital or cancer centre | 🞊 |
| 4 | Private Clinic | 🞊 |
| 5 | Private Hospital | 🞊 |
| 6 | Others, please specify **[______________________________________]** | 🞊 |
| 7 | I don't know exactly | 🞊 |

**Q3.** Is the doctor who told you the diagnosis ...

*Only one answer possible*

| 1 | The one who is currently following you for your ALK+ lung cancer | 🞊 |
| --- | --- | --- |
| 2 | Another doctor | 🞊 |

**Q4.** What is the **specialty of the doctor** who told you the diagnosis? *Only one answer possible*

**Q5. Have other health professionals been involved** in the announcement of your diagnosis, either on the day of the announcement or in the days that followed?

**NB: We are talking here about the people who intervened in the announcement of the diagnosis and not those who intervened later in your management of the disease**

*Several possible answers except for the last line*

|  |  | Specialty of the doctor who announced the diagnosis (only one answer possible) | Other stakeholders in the announcement (multiple responses possible) |
| --- | --- | --- | --- |
| 1 | An oncologist | 🞊 | 🞎 |
| 2 | A pulmonologist | 🞊 | 🞎 |
| 3 | A hospital nurse |  | 🞎 |
| 4 | An internist | 🞊 | 🞎 |
| 5 | A radiotherapist | 🞊 | 🞎 |
| 6 | A surgeon | 🞊 | 🞎 |
| 7 | The attending physician | 🞊 | 🞎 |
| 8 | A psychologist / psychiatrist |  | 🞎 |
| 9 | A health facilitator (ERI Meeting and information space) |  | 🞎 |
| 10 | A social worker |  | 🞎 |
| 11 | A radiologist | 🞊 | 🞎 |
| 12 | Others: *please specify*  …………………………………………………………… | 🞊 | 🞎 |
| 13 | I don't know this doctor's specialty | 🞊 |  |
| 14 | No, no other person involved in the announcement of the diagnosis |  | 🞊 |

**Q6.** Was this announcement of the diagnosis the subject of...

|  | *Only one possible answer per line* | Yes | No | I don't remember |
| --- | --- | --- | --- | --- |
| 1 | A specific consultation / an announcement consultation | 🞊 | 🞊 | 🞊 |
| 2 | An appointment with an announcement nurse on the same day or a few days later | 🞊 | 🞊 | 🞊 |

**Q7.** Were you accompanied by **a loved one** when this diagnosis was announced to you?

*Only one answer possible*

| 1 | Yes | 🞊 |
| --- | --- | --- |
| 2 | No | 🞊 |
| 3 | I don't remember | 🞊 |

**Q8.** Would you say that when you were diagnosed with ALK+ lung cancer, you **felt well surrounded, accompanied** by the medical team?

*Only one answer possible*

| 1 | Yes, absolutely | 🞊 |
| --- | --- | --- |
| 2 | Yes, partially | 🞊 |
| 3 | No | 🞊 |
| 4 | I don't remember | 🞊 |

**Q9.** Also when the diagnosis was announced and before starting your treatment, did you find that **the information that was provided to you to better understand** ...

|  | *Only one possible answer per line* | **Were sufficient** | **Were insufficient** | **I don't remember** |
| --- | --- | --- | --- | --- |
| 1 | ALK+ lung cancer | 🞊 | 🞊 | 🞊 |
| 2 | The treatment implemented | 🞊 | 🞊 | 🞊 |

**Q10.** Would you say that when you announced this diagnosis and before you started your treatment, you understood...

|  | *Only one possible answer per line* | **Yes, absolutely** | **Yes, partially** | **No** |
| --- | --- | --- | --- | --- |
| 1 | The particularities of ALK+ lung cancer | 🞊 | 🞊 | 🞊 |
| 2 | That this disease carries a risk of relapse | 🞊 | 🞊 | 🞊 |
| 3 | The reasons for choosing the treatment protocol set up for you | 🞊 | 🞊 | 🞊 |
| 4 | How your treatment works | 🞊 | 🞊 | 🞊 |
| 5 | The benefits you could expect from your treatment | 🞊 | 🞊 | 🞊 |
| 6 | Side effects you may experience during your treatment | 🞊 | 🞊 | 🞊 |
| 7 | That you should take your treatment chronically (i.e. every day) without stopping it even if you feel better | 🞊 | 🞊 | 🞊 |

**Q11.** When you announced your diagnosis and before starting your treatment, did the doctor tell you about other possible treatment(s) that could be implemented in the future in the event of a relapse?

*Only one answer possible*

| 1 | Yes, absolutely | 🞊 |
| --- | --- | --- |
| 2 | Yes, partially | 🞊 |
| 3 | No | 🞊 |
| 4 | I don't remember | 🞊 |

**Q12.** [if code 1 or 2 in Q11] What other possible treatment(s) did he tell you about?

*Several possible answers*

| **1** | Chemotherapy | 🞎 |
| --- | --- | --- |
| **2** | Other targeted therapy | 🞎 |
| **3** | Immunotherapy | 🞎 |
| **4** | Clinical Trial | 🞎 |
| **5** | Surgery | 🞎 |
| **6** | Radiotherapy | 🞎 |
| **7** | I don't remember exactly | 🞊 |

**Q13.** At the time of diagnosis or in the days that followed, did the medical team give you an information material/brochure

*Only one possible answer per line*

|  |  | **Yes** | **No** | **I don't remember** |
| --- | --- | --- | --- | --- |
| 1 | About ALK+ lung cancer | 🞊 | 🞊 | 🞊 |
| 2 | On the treatment put in place | 🞊 | 🞊 | 🞊 |
| 3 | On the different supportive care that can be offered | 🞊 | 🞊 | 🞊 |
| 4 | On patient associations | 🞊 | 🞊 | 🞊 |

***Note on supportive care:*** *supportive care is all the care and support offered to sick people throughout the disease (access to a dietician, a pain specialist, adapted physical activity, a psychologist, etc.)*

**Q14.** Have you searched **for** additional information on your own following the diagnosis?

|  | *Only one response per line* | **Yes** | **No** |
| --- | --- | --- | --- |
| 1 | About ALK+ lung cancer | 🞊 | 🞊 |
| 2 | On the treatment put in place | 🞊 | 🞊 |
| 3 | On the different supportive care that can be offered | 🞊 | 🞊 |
| 4 | On patient associations | 🞊 | 🞊 |

**Q14B.** [If ≥ 1 yes answer in Q14] How did you look for this information?

*Several possible answers*

| 1 | Via personal research on the internet | 🞎 |
| --- | --- | --- |
| 2 | Via patient associations | 🞎 |
| 3 | Via health professionals | 🞎 |
| 4 | Via the attending physician | 🞎 |
| 5 | Others, please specify....................................... | 🞎 |

***Note on supportive care:*** *supportive care is all the care and support offered to sick people throughout the disease (access to a dietician, a pain specialist, adapted physical activity, a psychologist, etc.)*

**Patients in L2+** [= code 2 or 3 in S4] [do not display]

**Let's start by talking about when the 1st diagnosis of your ALK+ lung cancer was made.**

**Q1A.** How old were you when the 1st diagnosis of your ALK+ lung cancer was made?

/__/__/Years

**Q2A.** Was he diagnosed **in the same establishment** in which you are currently being followed?

*Only one answer possible*

| 1 | Yes | 🞊 |
| --- | --- | --- |
| 2 | No | 🞊 |
| 3 | I don't know | 🞊 |

**Q2BA.** [if not in Q2] In which **school profile** was he diagnosed?

*Only one answer possible*

| 1 | University Hospital Center (CHU) / Regional University Hospital Center (CHUR) | 🞊 |
| --- | --- | --- |
| 2 | Anticancer Center / Center for the Fight Against Cancer (CLCC) | 🞊 |
| 3 | General Hospital; public health care establishment other than university hospital or cancer centre | 🞊 |
| 4 | Private Clinic | 🞊 |
| 5 | Private Hospital | 🞊 |
| 6 | Others, please specify **[______________________________________]** | 🞊 |
| 7 | **I don't know exactly** | 🞊 |

**Q3A.** Is the doctor who told you the 1st diagnosis ...

*Only one answer possible*

| 1 | The one who is currently following you for your ALK+ lung cancer | 🞊 |
| --- | --- | --- |
| 2 | Another doctor | 🞊 |

**Q4A.** What is the **specialty of the doctor** who told you the 1st diagnosis?

*Only one answer possible*

**Q5A. Have other health professionals been involved** in the announcement of your 1st diagnosis, either on the day of the announcement or in the following days?

**NB: We are talking here about the people who intervened in the announcement of the diagnosis and not those who intervened later in your management of the disease**

*Several possible answers except for the last line*

|  |  | Specialty of the doctor who announced the diagnosis (only one answer possible) | Other stakeholders in the announcement (multiple responses possible) |
| --- | --- | --- | --- |
| 1 | An oncologist | 🞊 | 🞎 |
| 2 | A pulmonologist | 🞊 | 🞎 |
| 3 | A hospital nurse |  | 🞎 |
| 4 | An internist | 🞊 | 🞎 |
| 5 | A radiotherapist | 🞊 | 🞎 |
| 6 | A surgeon | 🞊 | 🞎 |
| 7 | The attending physician | 🞊 | 🞎 |
| 8 | A psychologist / psychiatrist |  | 🞎 |
| 9 | A health facilitator (ERI Meeting and information space) |  | 🞎 |
| 10 | A social worker |  | 🞎 |
| 11 | A radiologist | 🞊 | 🞎 |
| 12 | Others: *please specify*  …………………………………………………………… | 🞊 | 🞎 |
| 13 | I don't know this doctor's specialty | 🞊 |  |
| 14 | No, no other person involved in the announcement of the diagnosis |  | 🞊 |

**Q6A.** Was this announcement of your 1st diagnosis the subject of ...

|  | *Only one possible answer per line* | Yes | No | I don't remember |
| --- | --- | --- | --- | --- |
| 1 | A specific consultation / an announcement consultation | 🞊 | 🞊 | 🞊 |
| 2 | An appointment with an announcement nurse on the same day or a few days later | 🞊 | 🞊 | 🞊 |

**Q7A.** Were you accompanied by a loved one when this 1st diagnosis was announced to you?

*Only one answer possible*

| 1 | Yes | 🞊 |
| --- | --- | --- |
| 2 | No | 🞊 |
| 3 | I don't remember | 🞊 |

**Q8A.** Would you say that when you were diagnosed with your ALK+ lung cancer, you **felt well surrounded, accompanied** by the medical team?

*Only one answer possible*

| 1 | Yes, absolutely | 🞊 |
| --- | --- | --- |
| 2 | Yes, partially | 🞊 |
| 3 | No | 🞊 |
| 4 | I don't remember | 🞊 |

**Q9A.** Also when the diagnosis was announced and before starting your treatment, did you find that **the information that was provided to you to better understand** ...

|  | *Only one possible answer per line* | **Were sufficient** | **Were insufficient** | **I don't remember** |
| --- | --- | --- | --- | --- |
| 1 | ALK+ lung cancer | 🞊 | 🞊 | 🞊 |
| 2 | The treatment implemented | 🞊 | 🞊 | 🞊 |

**Q10A.** Would you say that when this diagnosis was announced and before you started your treatment, you understood...

|  | *Only one possible answer per line* | **Yes, absolutely** | **Yes, partially** | **No** |
| --- | --- | --- | --- | --- |
| 1 | The particularities of ALK+ lung cancer | 🞊 | 🞊 | 🞊 |
| 2 | That this disease carries a risk of relapse | 🞊 | 🞊 | 🞊 |
| 3 | The reasons for choosing the treatment protocol set up for you | 🞊 | 🞊 | 🞊 |
| 4 | How your treatment works | 🞊 | 🞊 | 🞊 |
| 5 | The benefits you could expect from your treatment | 🞊 | 🞊 | 🞊 |
| 6 | Side effects you may experience during your treatment | 🞊 | 🞊 | 🞊 |
| 7 | That you should take your treatment chronically (i.e. every day) without stopping it even if you feel better | 🞊 | 🞊 | 🞊 |

**Q11A.** When the diagnosis was announced and before starting your treatment, did the doctor talk to you about other possible treatment(s) that could be implemented in the future in the event of a relapse?

*Only one answer possible*

| 1 | Yes, absolutely | 🞊 |
| --- | --- | --- |
| 2 | Yes, partially | 🞊 |
| 3 | No | 🞊 |
| 4 | I don't remember | 🞊 |

**Q12A.** [if code 1 or 2 in Q11A] What other possible treatment(s) did he tell you about?

*Several possible answers*

| **1** | Chemotherapy | 🞎 |
| --- | --- | --- |
| **2** | Other targeted therapy | 🞎 |
| **3** | Immunotherapy | 🞎 |
| **4** | Clinical Trial | 🞎 |
| **5** | Surgery | 🞎 |
| **6** | Radiotherapy | 🞎 |
| **7** | I don't remember exactly | 🞊 |

**Q13A.** At the time of diagnosis or in the days that followed, did the medical team give you an information material/brochure

*Only one possible answer per line*

|  |  | **Yes** | **No** | **I don't remember** |
| --- | --- | --- | --- | --- |
| 1 | About ALK+ lung cancer | 🞊 | 🞊 | 🞊 |
| 2 | On the treatment put in place | 🞊 | 🞊 | 🞊 |
| 3 | On the different supportive care that can be offered | 🞊 | 🞊 | 🞊 |
| 4 | On patient associations | 🞊 | 🞊 | 🞊 |

***Note on supportive care:*** *supportive care is all the care and support offered to sick people throughout the disease (access to a dietician, a pain specialist, adapted physical activity, a psychologist, etc.)*

**Q14A.** Have you looked for **additional information on your own**, following this 1st diagnosis...

|  | *Only one response per line* | **Yes** | **No** |
| --- | --- | --- | --- |
| 1 | About ALK+ lung cancer | 🞊 | 🞊 |
| 2 | On the treatment put in place | 🞊 | 🞊 |
| 3 | On the different supportive care that can be offered | 🞊 | 🞊 |
| 4 | On patient associations | 🞊 | 🞊 |

**Q14B2.** [If ≥ 1 answer yes in Q14A] How did you look for this information?

*Several possible answers*

| 1 | Via personal research on the internet | 🞎 |
| --- | --- | --- |
| 2 | Via patient associations | 🞎 |
| 3 | Via health professionals | 🞎 |
| 4 | Via the attending physician | 🞎 |
| 5 | Others, please specify....................................... | 🞎 |

***Note on supportive care:*** *supportive care is all the care and support offered to sick people throughout the disease (access to a dietician, a pain specialist, adapted physical activity, a psychologist, etc.)*

**Now let's talk about the announcement of your relapse. If you have experienced several relapses, we will talk about your last relapse here.**

**Q15.** How old were you when your relapse was diagnosed?

/__/__/Years

**Q16.** What is the **specialty of the doctor** who told you you had relapsed?

*Only one answer possible*

**Q17. Have other health professionals intervened** in the announcement of your relapse, either on the day of the announcement or a little later?

**NB: We are talking here about the people who intervened in the announcement of your relapse**

*Several possible answers except for the last line*

|  |  | Specialty of the doctor who announced your relapse *(only one answer possible)* | Other stakeholders in the announcement of the relapse *(several possible answers)* |
| --- | --- | --- | --- |
| 1 | An oncologist | 🞊 | 🞎 |
| 2 | A pulmonologist | 🞊 | 🞎 |
| 3 | A hospital nurse |  | 🞎 |
| 4 | An internist | 🞊 | 🞎 |
| 5 | A radiotherapist | 🞊 | 🞎 |
| 6 | A surgeon | 🞊 | 🞎 |
| 7 | The attending physician | 🞊 | 🞎 |
| 8 | A psychologist / psychiatrist |  | 🞎 |
| 9 | A health facilitator (ERI Meeting and information space) |  | 🞎 |
| 10 | A social worker |  | 🞎 |
| 11 | A radiologist | 🞊 | 🞎 |
| 12 | Others: *please specify*  …………………………………………………………… | 🞊 | 🞎 |
| 13 | I don't know this doctor's specialty | 🞊 |  |
| 14 | No, no other player in the announcement of the relapse |  | 🞊 |

**Q18.** Was this announcement of your relapse the subject of

|  | *Only one possible answer per line* | **Yes** | **No** | **I don't remember** |
| --- | --- | --- | --- | --- |
| 1 | A specific consultation / an announcement consultation | 🞊 | 🞊 | 🞊 |
| 2 | An appointment with an announcement nurse on the same day or a few days later | 🞊 | 🞊 | 🞊 |

**Q19.** Were you accompanied by a loved one when the relapse was announced to you?

*Only one answer possible*

| 1 | Yes | 🞊 |
| --- | --- | --- |
| 2 | No | 🞊 |
| 3 | I don't remember | 🞊 |

**Q20.** Would you say that when this relapse was announced, you felt **well surrounded, accompanied** by the medical team?

*Only one answer possible*

| 1 | Yes, absolutely | 🞊 |
| --- | --- | --- |
| 2 | Yes, partially | 🞊 |
| 3 | No | 🞊 |
| 4 | I don't remember | 🞊 |

**Q21.** Also when announcing your relapse and before starting your new treatment, did you find that **the information provided to you to better understand** ...

*Only one possible answer per line*

|  |  | **Were sufficient** | **Were insufficient** | **I don't remember** |
| --- | --- | --- | --- | --- |
| 1 | The progression of your disease | 🞊 | 🞊 | 🞊 |
| 2 | The new treatment protocol put in place | 🞊 | 🞊 | 🞊 |

**Q22.** Would you say that, when you announced your relapse and before you started your new treatment, you understood

|  | *Only one possible answer per line* | **Yes, absolutely** | **Yes, partially** | **No** |
| --- | --- | --- | --- | --- |
| 1 | The reasons for choosing the new treatment protocol implemented | 🞊 | 🞊 | 🞊 |
| 2 | How does the new treatment work | 🞊 | 🞊 | 🞊 |
| 3 | The benefits you could expect from this new treatment | 🞊 | 🞊 | 🞊 |
| 4 | Side effects you may have experienced with this new treatment | 🞊 | 🞊 | 🞊 |

**Q23.** When announcing your relapse and before starting your new treatment, did the doctor tell you about other possible treatment(s) that could be implemented in the future in the event of a new relapse? *Only one answer possible*

| 1 | Yes, absolutely | 🞊 |
| --- | --- | --- |
| 2 | Yes, partially | 🞊 |
| 3 | No | 🞊 |
| 4 | I don't remember | 🞊 |

**Q24.** [if code 1 or 2 in Q23] What other possible treatment(s) did he tell you about?

*Several possible answers*

| **1** | Chemotherapy | 🞎 |
| --- | --- | --- |
| **2** | Other targeted therapy | 🞎 |
| **3** | Immunotherapy | 🞎 |
| **4** | Clinical Trial | 🞎 |
| **5** | Surgery | 🞎 |
| **6** | Radiotherapy | 🞎 |
| **7** | I don't remember exactly | 🞊 |

**Q25.** When you announced your relapse or in the days that followed, did the medical team give you an information material / a brochure

*Only one possible answer per line*

|  |  | **Yes** | **No** | **I don't remember** |
| --- | --- | --- | --- | --- |
| 1 | About ALK+ lung cancer | 🞊 | 🞊 | 🞊 |
| 2 | On the treatment put in place | 🞊 | 🞊 | 🞊 |
| 3 | On the different supportive care that can be offered | 🞊 | 🞊 | 🞊 |
| 4 | On patient associations | 🞊 | 🞊 | 🞊 |

***Note on supportive care:*** *supportive care is all the care and support offered to sick people throughout the disease (access to a dietician, a pain specialist, adapted physical activity, a psychologist, etc.)*

**Q26.** Have you searched **for** additional information on your own following your relapse... *Only one response per line*

|  |  | **Yes** | **No** |
| --- | --- | --- | --- |
| 1 | About ALK+ lung cancer | 🞊 | 🞊 |
| 2 | On the treatment put in place | 🞊 | 🞊 |
| 3 | On the different supportive care that can be offered | 🞊 | 🞊 |
| 4 | On patient associations | 🞊 | 🞊 |

***Note on supportive care:*** *supportive care is all the care and support offered to sick people throughout the disease (access to a dietician, a pain specialist, adapted physical activity, a psychologist, etc.)*

**Q26B.** [If ≥ 1 answer yes in Q26] How did you look for this information?

*Several possible answers*

| 1 | Via personal research on the internet | 🞎 |
| --- | --- | --- |
| 2 | Via patient associations | 🞎 |
| 3 | Via health professionals | 🞎 |
| 4 | Via the attending physician | 🞎 |
| 5 | Others, please specify....................................... | 🞎 |

**Part B. ALK+ lung cancer treatment and its perception [7-8']**

**Now let's talk about your current treatment with targeted therapy and your medical follow-up.**

Reminder of what is a targeted therapy [TKI – Tyrosine Kinase inhibitor with one of the following treatment names: Alecensa® (Alectinib), Alunbrig® (Brigatinib), Lorviqua® (Lorlatinib), Xalkori® (Crizotinib), Zykadia® (Ceretinib)]

**Q27.** Could you indicate the name of your current targeted therapy?

| 1 | Alecensa® (Alectinib) | 🞊 |
| --- | --- | --- |
| 2 | Alunbrig® (Brigatinib) | 🞊 |
| 3 | Lorviqua® (Lorlatinib) | 🞊 |
| 4 | Xalkori® (Crizotinib) | 🞊 |
| 5 | Zykadia® (Ceretinib) | 🞊 |
| 6 | I don't know | 🞊 |

**Q27B.** When did you start the targeted therapy treatment you are currently taking ?

/__/__/ /__/__/__/__/ (please enter the two digits of the month and then the year – if you don't remember the month, you can just enter the year)

🞊 I don't know

**Q28.** [for L2+ patients] What treatment(s) have you received in the past to treat your ALK+ lung cancer?

*Several possible answers*

| 1 | Chemotherapy | 🞎 |
| --- | --- | --- |
| 2 | Targeted therapy | 🞎 |
| 3 | Radiotherapy | 🞎 |
| 4 | Immunotherapy | 🞎 |
| 5 | Surgery | 🞎 |
| 6 | Treatment in a clinical trial | 🞎 |
| 7 | Others, please specify /_______________/ | 🞎 |
| 8 | I don't remember | 🞎 |

**Q28B.** [if code 2 in Q28] Could you indicate the name of the targeted therapy you have received in the past?

| 1 | Alecensa® (Alectinib) | 🞊 |
| --- | --- | --- |
| 2 | Alunbrig® (Brigatinib) | 🞊 |
| 3 | Lorviqua® (Lorlatinib) | 🞊 |
| 4 | Xalkori® (Crizotinib) | 🞊 |
| 5 | Zykadia® (Ceretinib) | 🞊 |
| 6 | I don't know | 🞊 |

**Q29.** Do you feel that you are today**,** about your targeted therapy treatment...

*Only one answer possible*

| 1 | Very knowledgeable | 🞊 |
| --- | --- | --- |
| 2 | Fairly knowledgeable | 🞊 |
| 3 | Somewhat uninformed | 🞊 |
| 4 | Very uninformed | 🞊 |

**Q30.** Still concerning your treatment with targeted therapy, have you received any information in your healthcare establishment **other than that communicated to you by your oncologist? (For example, through: therapeutic education sessions (TPE), information workshops, medication reconciliation with a hospital pharmacist)**

*Only one answer possible*

| 1 | Yes | 🞊 |
| --- | --- | --- |
| 2 | No | 🞊 |
| 3 | I don't remember | 🞊 |

**Q31.** Here are some criteria that can be associated with treatment. For each of these criteria, how would you rate each of these treatments in relation to the image you have of them?

*1, means that you do not attribute this criterion to the corresponding treatment at all. 10, that you strongly attribute this criterion to the corresponding treatment. Intermediate grades allow you to modulate your judgment.*

|  | *[programming: cursor to be provided]* | **Targeted therapy** | **Chemotherapy** |
| --- | --- | --- | --- |
| 1 | Quality of life preserved with this treatment | \|___\| 🞊NSP | \|___\|¤NSP |
| 2 | Favorable balance between benefits and constraints with this treatment | \|___\| 🞊NSP | \|___\|¤NSP |
| 3 | Tolerable side effects | \|___\| 🞊NSP | \|___\|¤NSP |
| 4 | Treatment that gives hope | \|___\| 🞊NSP | \|___\|¤NSP |
| 5 | Ease of administration of this treatment | \|___\| 🞊NSP | \|___\|¤NSP |
| 6 | Treatment well monitored by the medical team | \|___\| 🞊NSP | \|___\|¤NSP |
| 7 | Treatment that allows you to live almost normally | \|___\| 🞊NSP | \|___\|¤NSP |
| 8 | Treatment with long-lasting effectiveness | \|___\| 🞊NSP | \|___\|¤NSP |

**Q32.** Today, how often do you see the doctor who follows you for your cancer?

All |___| month

**Q33**. How long do these follow-up consultations last, on average?

|___| Minutes

**Q34**. Has telephone support been set up for the follow-up of your current treatment? (Regular phone calls from a nurse at your healthcare facility)

*Only one answer possible*

| 1 | Yes | 🞊 |
| --- | --- | --- |
| 2 | No | 🞊 |

**Q34B**. [If not in Q34] Would you have liked to have had telephone support set up for the follow-up of your current treatment?

*Only one answer possible*

| 1 | Yes | 🞊 |
| --- | --- | --- |
| 2 | No | 🞊 |

**Q35.** Here are different statements about the monitoring of your disease and your current treatment. For each of them, please indicate if you agree by ticking the box that best corresponds to how you feel

| *Only one response per line* | | Strongly disagree | Rather disagree | Tend to agree | Totally agree |
| --- | --- | --- | --- | --- | --- |
| 1 | The duration of my follow-up consultations is, as a rule, sufficient | 🞊 | 🞊 | 🞊 | 🞊 |
| 2 | I don't miss any follow-up medical appointments because being monitored regularly reassures me | 🞊 | 🞊 | 🞊 | 🞊 |
| 3 | The time between the results of the CT scan and the consultation with the oncologist is often too long and a source of anxiety and stress | 🞊 | 🞊 | 🞊 | 🞊 |
| 4 | I always experience the check-ups with great concern | 🞊 | 🞊 | 🞊 | 🞊 |
| 5 | [if yes in Q34] The telephone support allowed me to better understand my treatment | 🞊 | 🞊 | 🞊 | 🞊 |
| 6 | [if yes in Q34] The telephone support allowed me to better manage the side effects of my treatment | 🞊 | 🞊 | 🞊 | 🞊 |
| 7 | As soon as I feel better, I want to stop taking my medication | 🞊 | 🞊 | 🞊 | 🞊 |
| 8 | I sometimes feel like my treatment is doing me more harm than good | 🞊 | 🞊 | 🞊 | 🞊 |

**Q36.** During follow-up consultations with the doctor who is treating you for your cancer, what are the points you discuss?

| *Only one response per line* | | **Very often** | **From time to time** | **Rarely** | **Never** |
| --- | --- | --- | --- | --- | --- |
| 1 | The results of the CT scan | 🞊 | 🞊 | 🞊 | 🞊 |
| 2 | The physical symptoms of your disease: fatigue, pain, etc. | 🞊 | 🞊 | 🞊 | 🞊 |
| 3 | The concrete repercussions of the disease on your daily life, such as: giving up certain activities, professional or family difficulties,... | 🞊 | 🞊 | 🞊 | 🞊 |
| 4 | Side effects of your treatment | 🞊 | 🞊 | 🞊 | 🞊 |
| 5 | The effectiveness of your treatment | 🞊 | 🞊 | 🞊 | 🞊 |
| 6 | Your morale, experience of the disease on a psychological and emotional level | 🞊 | 🞊 | 🞊 | 🞊 |

**Q37.** Who are all of the following professionals who are currently following you in the context of your ALK+ lung cancer? *Several possible answers*

**Q37B.** And who are the ones who provide you with the most support in the face of the impact of the disease on your daily life? *Several possible answers*

|  |  | **All the current stakeholders in the management of my cancer** | **Those who have given me the most support in the face of the impact of the disease on my daily life [filter Q37]** |
| --- | --- | --- | --- |
| 1 | Oncologist | 🞎 | 🞎 |
| 2 | Pulmonologist | 🞎 | 🞎 |
| 3 | Internist | 🞎 | 🞎 |
| ~~4~~ | Radiotherapist | ~~🞎~~ | ~~🞎~~ |
| 5 | General practitioner / general practitioner | 🞎 | 🞎 |
| 6 | Surgeon | 🞎 | 🞎 |
| 7 | Psychologist, psychiatrist | 🞎 | 🞎 |
| 8 | Health Facilitator (ERI Meeting and Information Space) | 🞎 | 🞎 |
| 9 | Hospital Nurse, Clinic Nurse | 🞎 | 🞎 |
| 10 | Advanced Practice Nurse (APN) | 🞎 | 🞎 |
| 11 | Home Nurse | 🞎 | 🞎 |
| 12 | Dietician/Nutritionist | 🞎 | 🞎 |
| 13 | Social worker | 🞎 | 🞎 |
| 14 | Physiotherapist | 🞎 | 🞎 |
| 15 | Sexologist | 🞎 | 🞎 |
| 16 | Pain algologist | 🞎 | 🞎 |
| 17 | Psychomotor therapist | 🞎 | 🞎 |
| 18 | Occupational therapist | 🞎 | 🞎 |
| 19 | Entourage (family / friends) | 🞎 | 🞎 |
| 20 | Patient Associations | 🞎 | 🞎 |
| 21 | Other patients met at the hospital | 🞎 | 🞎 |
| 22 | Life assistant | 🞎 | 🞎 |
| 23 | Esthetician / Esthetician | 🞎 | 🞎 |
| 24 | APA (Adapted Physical Activity) Instructor / Educator | 🞎 | 🞎 |
| 25 | Other: *please specify*  ……………………………………………………………………………………………. | 🞎 | 🞎 |
| 26 | I am not receiving any support for the impact of the disease on my daily life |  | 🞊 |

**Q37D.** [if the following stakeholders listed in Q37] Do the following stakeholders in the management of your loved one's cancer practice?

*Only one possible answer per line*

|  |  | **At the hospital or clinic** in which your loved one is being followed | Outside the hospital or clinic in which your loved one is being cared for **(= in the city)** |
| --- | --- | --- | --- |
| 7 | Psychologist, psychiatrist | 🞊 | 🞊 |
| 11 | Dietician/Nutritionist | 🞊 | 🞊 |
| 12 | Social worker | 🞊 | 🞊 |
| 13 | Physiotherapist | 🞊 | 🞊 |
| 14 | Sexologist | 🞊 | 🞊 |
| 15 | Pain algologist | 🞊 | 🞊 |
| 16 | Psychomotor therapist | 🞊 | 🞊 |
| 17 | Occupational therapist | 🞊 | 🞊 |
| 22 | Esthetician / Esthetician | 🞊 | 🞊 |

**Part C. The impact of the disease and its management on your daily life (16-18')**

Now, we would like to better understand and better understand how the disease and its management/treatment affect the lives of patients.

**Q38**. If you had to give a score on a scale of 1 to 10 to **describe the overall impact of your disease and its management/treatment on your daily life over the past month**, what score would you give?

*You have scores from 1 to 10 to answer this question. 1 means that the disease has not limited your daily activities at all; 10 that it has severely limited them. Intermediate grades allow you to modulate your judgment*

| ***1*** 💣 \| ***2*** 💣 \| ***3*** 💣 \| ***4*** 💣 \| ***5*** 💣 \| ***6*** 💣 \| ***7*** 💣 \| ***8*** 💣 \| ***9*** 💣 \| ***10*** 💣 | | |
| --- | --- | --- |
| ***Didn't limit your daily activities at all*** | ***Severely limited your daily activities*** |  |

**Q39**. Also in the last month, have you encountered any **adverse events with your targeted therapy**?

*Only one answer possible*

| 1 | Yes | 🞊 |
| --- | --- | --- |
| 2 | No | 🞊 |
| 3 | I don't know | 🞊 |

**Q39B.** [if code 1 in Q39] What has been the impact of these adverse events – encountered with your targeted therapy – on your daily life?

*You have scores from 1 to 10 to answer this question. 1 means that these adverse events did not limit your daily activities at all; 10 that they have severely limited them. Intermediate grades allow you to modulate your judgment*

| ***1*** 💣 \| ***2*** 💣 \| ***3*** 💣 \| ***4*** 💣 \| ***5*** 💣 \| ***6*** 💣 \| ***7*** 💣 \| ***8*** 💣 \| ***9*** 💣 \| ***10*** 💣 | | |
| --- | --- | --- |
| ***Didn't limit your daily activities at all*** | ***Severely limited your daily activities*** |  |

**Q39C.** [if code 1 in Q39] Have your adverse events with your targeted therapy been managed?

| 1 | Yes for the majority of them | 🞊 |
| --- | --- | --- |
| 2 | Yes, but only for a minority of them | 🞊 |
| 3 | No support | 🞊 |

**Q39D.** [if code 1 or 2 in Q39C] Are you satisfied with this management of adverse events in your targeted therapy?

| 1 | Yes Absolutely | 🞊 |
| --- | --- | --- |
| 2 | Yes partially | 🞊 |
| 3 | No | 🞊 |

**Here are a number of concrete difficulties/repercussions of the disease on daily life that can be expressed by patients.**

A. Performing everyday gestures

**Q40.** Could you indicate if you are affected by some of these difficulties related to gestures/activities of daily living?

**Please select only the difficulties that you attribute to your ALK+ lung cancer**

*Several possible answers*

| 1 | It is difficult for me to maintain my home and/or exterior/to complete household chores | 🞎 |
| --- | --- | --- |
| 2 | It is difficult for me to prepare meals | 🞎 |
| 3 | It is difficult for me to wash and take care of myself | 🞎 |
| 4 | It's hard for me to eat | 🞎 |
| 5 | It's hard for me to stand for too long | 🞎 |
| 6 | It is difficult for me to use public transport | 🞎 |
| 7 | It is difficult for me to maintain physical and/or sporting activity | 🞎 |
| 8 | It's hard for me to focus on one activity | 🞎 |
| 9 | Other gestures/activities of daily living that are difficult to perform in connection with the disease, please specify............................................................... | 🞎 |
| 10 | I am not concerned by any difficulty in carrying out the gestures of daily life that would be related to the disease | 🞊 |

**Q40A.** [if ≠10 in Q40] Were you able to discuss these difficulties related to the gestures of daily life?

*Only one possible answer per column*

|  |  | With a health professional in the hospital or clinic who is following you for your cancer | With a healthcare professional outside the hospital or clinic where you are being treated for your cancer |
| --- | --- | --- | --- |
| **1** | Yes and he/she was very helpful | 🞊 | 🞊 |
| **2** | Yes, but he/she has not been particularly helpful | 🞊 | 🞊 |
| **3** | No, but I don't want to talk about this type of subject | 🞊 | 🞊 |
| **4** | No, but I would have liked to have been able to address this type of subject | 🞊 | 🞊 |

**Q40B.** [if ≠10 in Q40] To what extent do these difficulties related to the gestures of daily life affect your daily life?

**Please give a score from 1 to 10.**

*1 = my daily life is not affected at all by these difficulties*

*10 = My daily life is very affected by these difficulties*

*Intermediate grades allow you to modulate your judgment*

| ***1***  💣 | ***2***  💣 | ***3***  💣 | ***4***  💣 | ***5***  💣 | ***6***  💣 | ***7***  💣 | ***8***  💣 | ***9***  💣 | ***10***  💣 |
| --- | --- | --- | --- | --- | --- | --- | --- | --- | --- |

***My daily life is not affected at all***	***My daily life is very affected***

**Q40B.** [if ≠10 in Q40] To carry out certain daily tasks, do you benefit from ...

|  | *Only one possible answer per line* | **Yes** | **No** |
| --- | --- | --- | --- |
| 1 | Help from a loved one (family member, friend) | 🞊 | 🞊 |
| 2 | A domestic helper | 🞊 | 🞊 |
| 3 | Meals delivered at home | 🞊 | 🞊 |

**Q40C**. Are you currently receiving help, support from a loved one...

*Only one possible answer per line*

|  |  | Yes | No |
| --- | --- | --- | --- |
| 1 | To accompany you during your hospital trips | 🞊 | 🞊 |
| 2 | To support you morally | 🞊 | 🞊 |

**Q40D.** [if yes in Q40B line 1 or yes in line 1 or 2 in Q40C] To what extent does the help and support of your loved one allow you to live better with the disease

**Please give a score from 1 to 10.**

*1 = the help and support of my loved one does not have a positive influence on my experience of the disease*

*10 = the help and support of my loved one has a very positive influence on my experience of the disease*

*Intermediate grades allow you to modulate your judgment*

| ***1***  💣 | ***2***  💣 | ***3***  💣 | ***4***  💣 | ***5***  💣 | ***6***  💣 | ***7***  💣 | ***8***  💣 | ***9***  💣 | ***10***  💣 |
| --- | --- | --- | --- | --- | --- | --- | --- | --- | --- |

***Does not have a positive influence***	***Has a very positive impact***

**Q40E.** Do you drive a car?

*Only one answer possible*

| 1 | Yes, and I've been driving since I got sick | 🞊 |
| --- | --- | --- |
| 2 | I was driving before the disease but I have since stopped driving | 🞊 |
| 3 | No, I have never driven a car | 🞊 |

**Q40F** [if code 1 or 2 in Q40E] Has your cancer affected your ability to drive long distances?

*Only one answer possible*

| 1 | Yes | 🞊 |
| --- | --- | --- |
| 2 | No | 🞊 |

**Q40G.** [if code 2 in Q40E] For what reason(s) did you stop driving?

*Several possible answers*

| 1 | I don't feel capable of it anymore | 🞎 |
| --- | --- | --- |
| 2 | My doctor recommended it to me | 🞎 |
| 3 | I have brain metastases | 🞎 |
| 4 | I am taking medication that is incompatible with driving | 🞎 |
| 5 | Other, please specify............................................. | 🞎 |

**Q40H.** [if code 1 or 2 in Q40E] Has the subject of driving been discussed with the medical team that is treating you for your cancer?

*Only one answer possible*

| 1 | Yes | 🞊 |
| --- | --- | --- |
| 2 | No | 🞊 |

**Q40I.** [if code 1 or 2 in Q40E] Have you carried out a medical examination of fitness to drive?

*Only one answer possible*

| 1 | Yes | 🞊 |
| --- | --- | --- |
| 2 | No | 🞊 |

B. Professional life

**Q41**. What is your current professional situation?

*Several possible answers*

| 1 | In full-time professional activity | 🞎 |
| --- | --- | --- |
| 2 | In part-time employment | 🞎 |
| 3 | On sick leave | 🞎 |
| 4 | On disability | 🞎 |
| 5 | Looking for a job | 🞎 |
| 6 | School / Student | 🞎 |
| 7 | Retired | 🞎 |
| 8 | Non-active (without profession, ...) | 🞎 |

**Q41B.** [if code 2 or 3 or 4 or 6 or 7 in Q41] Is your current situation [part-time or work stoppage or disability or job search or inactive] related to your ALK+ lung cancer?

| 1 | Yes | 🞊 |
| --- | --- | --- |
| 2 | No | 🞊 |

**Q41C.** [if in a relationship] What is your spouse's current situation?

*Only one answer possible*

| 1 | In full-time professional activity | 🞊 |
| --- | --- | --- |
| 2 | In part-time employment | 🞊 |
| 3 | On sick leave | 🞊 |
| 4 | Looking for a job | 🞊 |
| 5 | School / Student | 🞊 |
| 6 | Retired | 🞊 |
| 7 | Non-active (without profession, ...) | 🞊 |

**Q41D.** [if code 2 or 3 or 4 or 6 or 7 in Q41C] Is your spouse's current situation [part-time or work stoppage or job search or inactive] related to your ALK+ lung cancer?

| 1 | Yes | 🞊 |
| --- | --- | --- |
| 2 | No | 🞊 |

**Q41E.** [if code 1 or 2 in Q41] Have you been put in touch with the following professionals when you resumed your activity?

| *Only one possible answer per line* | Yes | No | I don't remember |
| --- | --- | --- | --- |
| Occupational physician | 🞊 | 🞊 | 🞊 |
| Social worker | 🞊 | 🞊 | 🞊 |

**Q41F.** [if code 1 or 2 in Q41] Have you been able to discuss the subject of resuming your professional activity?

*Only one possible answer per column*

|  |  | With a health professional in the hospital or clinic who is following you for your cancer | With a healthcare professional outside the hospital or clinic where you are being treated for your cancer |
| --- | --- | --- | --- |
| **1** | Yes and he/she was very helpful | 🞊 | 🞊 |
| **2** | Yes, but he/she has not been particularly helpful | 🞊 | 🞊 |
| **3** | No, but I don't want to talk about this type of subject | 🞊 | 🞊 |
| **4** | No, but I would have liked to have been able to address this type of subject | 🞊 | 🞊 |

**Q41G***.* [if code 1 or 2 in Q41] Did you carry out a pre-resumption visit before resuming your work?

| **1** | Yes | 🞊 |
| --- | --- | --- |
| **2** | No, it was not offered to me and I had never heard of it | 🞊 |
| **3** | No, it was offered to me but I didn't want to do it | 🞊 |

**Q42.** Could you indicate if you are affected by some of these professional difficulties?

**Please select only the difficulties that you attribute to your ALK+ lung cancer**

*Several possible answers*

| 1 | I had to adapt my working hours and switch to part-time work | 🞎 |
| --- | --- | --- |
| 2 | I lost a job because of my illness | 🞎 |
| 3 | I have to deal with frequent sick leave | 🞎 |
| 4 | I had to adjust my working hours because of my illness | 🞎 |
| 5 | I had to stop working because of my illness | 🞎 |
| 6 | I had to change position/profession | 🞎 |
| 7 | I'm having trouble finding a new job | 🞎 |
| 8 | I'm afraid of losing my job | 🞎 |
| 9 | I am having difficulty progressing professionally / I have been refused a promotion | 🞎 |
| 10 | I have relationship difficulties with my employer due to the disease | 🞎 |
| 11 | I have relationship difficulties with my colleagues related to the disease | 🞎 |
| 12 | I find it difficult to keep up with my professional obligations as before | 🞎 |
| 13 | I have difficulty concentrating at work, I don't have all my intellectual/cognitive abilities | 🞎 |
| 14 | Other professional difficulties related to the disease, please specify............................................................... | 🞎 |
| 15 | I am not affected by any professional difficulties that would be related to the disease | 🞊 |
| 16 | I am not working now or since I became ill | 🞊 |

**Q42B**. [if ≠15 or 16 in Q42] Have you been able to discuss your professional difficulties?

*Only one possible answer per column*

|  |  | With a health professional in the hospital or clinic who is following you for your cancer | With a healthcare professional outside the hospital or clinic where you are being treated for your cancer |
| --- | --- | --- | --- |
| **1** | Yes and he/she was very helpful | 🞊 | 🞊 |
| **2** | Yes, but he/she has not been particularly helpful | 🞊 | 🞊 |
| **3** | No, but I don't want to talk about this type of subject | 🞊 | 🞊 |
| **4** | No, but I would have liked to have been able to address this type of subject | 🞊 | 🞊 |

**Q42C.** [if ≠15 or 16 in Q42] To what extent is your professional life affected by the disease?

**Please give a score from 1 to 10.**

*1 = my professional life is not affected by the disease at all*

*10 = my professional life is very affected by the disease*

*Intermediate grades allow you to modulate your judgment*

| ***1***  💣 | ***2***  💣 | ***3***  💣 | ***4***  💣 | ***5***  💣 | ***6***  💣 | ***7***  💣 | ***8***  💣 | ***9***  💣 | ***10***  💣 |
| --- | --- | --- | --- | --- | --- | --- | --- | --- | --- |

***Not affected at all	Is very affected***

**Q42D.** [if ≠15 or 16 in Q42] To what extent do the adverse effects of your treatment affect your professional life?

**Please give a score from 1 to 10.**

*1 = my professional life is not affected at all by the side effects of my treatment*

*10 = my professional life is very affected by the side effects of my treatment*

*Intermediate grades allow you to modulate your judgment*

| ***1***  💣 | ***2***  💣 | ***3***  💣 | ***4***  💣 | ***5***  💣 | ***6***  💣 | ***7***  💣 | ***8***  💣 | ***9***  💣 | ***10***  💣 |
| --- | --- | --- | --- | --- | --- | --- | --- | --- | --- |

***Not affected at all***	***Is very affected***

**Q42E.** [if ≠15 or 16 in Q42] Does your employer understand the impact of your illness on your work?

*Only one answer possible*

| 1 | Yes, he/she understands very well | 🞊 |
| --- | --- | --- |
| 2 | Yes, he/she understands quite well | 🞊 |
| 3 | No, he/she doesn't understand well enough | 🞊 |
| 4 | No, he/she doesn't understand at all | 🞊 |
| **5** | My employer doesn't know I have cancer | 🞊 |

**Q42F.** [if ≠15 or 16 in Q42] Has your employer been a caregiver to adapt your working conditions?

*Only one answer possible*

| **1** | Yes | 🞊 |
| --- | --- | --- |
| **2** | No | 🞊 |
| **3** | My employer doesn't know I have cancer | 🞊 |

**Q42G.** [if ≠ 16 in Q42] Have you been able to benefit from a liaison interview at your request or that of your employer following your sick leave?

***NB. The liaison interview is a meeting organised between the employer, the employee and the occupational health service, most often represented by the occupational physician***

*Only one answer possible*

| **1** | Yes | 🞊 |
| --- | --- | --- |
| **2** | No | 🞊 |
| **3** | I didn't have any sick leave | 🞊 |

**Q42H.** [if code 5 in Q41] To what extent is your school/student life affected by the disease?

**Please give a score from 1 to 10.**

*1 = my school/student life is not affected by the disease at all*

*10 = my school/student life is very affected by the disease*

*Intermediate grades allow you to modulate your judgment*

| ***1***  💣 | ***2***  💣 | ***3***  💣 | ***4***  💣 | ***5***  💣 | ***6***  💣 | ***7***  💣 | ***8***  💣 | ***9***  💣 | ***10***  💣 |
| --- | --- | --- | --- | --- | --- | --- | --- | --- | --- |

***Not affected at all	Is very affected***

C. Couple life, intimate life

**Q43.** Could you indicate if you are concerned by some of these difficulties in your life as a couple, intimate life?

**Please select only the difficulties that you attribute to your ALK+ lung cancer**

| 1 | I have had difficulty investing in a life as a couple since the illness | 🞎 |
| --- | --- | --- |
| 2 | The disease generates tension in my life as a couple | 🞎 |
| 3 | I feel that my spouse doesn't understand what I'm going through | 🞎 |
| 4 | I feel like I'm a burden to my spouse | 🞎 |
| 5 | The disease has strengthened my life as a couple | 🞎 |
| 6 | The disease precipitated my breakup with my spouse | 🞎 |
| 7 | I have been experiencing a drop or loss of libido since the disease | 🞎 |
| 8 | Other marital difficulties related to the disease, please specify............................................................... | 🞎 |
| 9 | I am not concerned by any difficulty in my life as a couple that would be related to the disease | 🞊 |
| 10 | I haven't been in a relationship since I got sick | 🞊 |

**Q43B**. [if ≠9 in Q43] Were you able to discuss your relationship difficulties?

*Only one possible answer per column*

|  |  | With a health professional in the hospital or clinic who is following you for your cancer | With a healthcare professional outside the hospital or clinic where you are being treated for your cancer |
| --- | --- | --- | --- |
| **1** | Yes and he/she was very helpful | 🞊 | 🞊 |
| **2** | Yes, but he/she has not been particularly helpful | 🞊 | 🞊 |
| **3** | No, but I don't want to talk about this type of subject | 🞊 | 🞊 |
| **4** | No, but I would have liked to have been able to address this type of subject | 🞊 | 🞊 |

**Q43C**. [If the patient has sexual difficulties=code 7 in Q43] Has your healthcare team offered you to consult a sexologist or any documentation to overcome the difficulties you are experiencing sexually?

*Only one answer possible*

| **1** | Yes | 🞊 |
| --- | --- | --- |
| **2** | No | 🞊 |

**Q43D.** [if ≠ code 9 in Q43] To what extent is your life as a couple affected by the disease?

**Please give a score from 1 to 10.**

*1 = my life as a couple is not affected at all by the disease*

*10 = my life as a couple is very affected by the disease*

*Intermediate grades allow you to modulate your judgment*

| ***1***  💣 | ***2***  💣 | ***3***  💣 | ***4***  💣 | ***5***  💣 | ***6***  💣 | ***7***  💣 | ***8***  💣 | ***9***  💣 | ***10***  💣 |
| --- | --- | --- | --- | --- | --- | --- | --- | --- | --- |

***Not affected at all	Is very affected***

D. Family and Friendship Life

**Q44.** Could you indicate if you are affected by some of these difficulties in your family and/or friendship life?

**Please select only the difficulties that you attribute to your ALK+ lung cancer**

| 1 | I don't give my family the time I would like | 🞎 |
| --- | --- | --- |
| 2 | I don't give my friends the time I'd like | 🞎 |
| 3 | My family doesn't understand or doesn't understand that I can't be available as often as I'd like | 🞎 |
| 4 | My friends don't understand or don't understand that I can't be available as often as I'd like | 🞎 |
| 5 | I feel like I'm a burden to my family | 🞎 |
| 6 | I have difficulty talking about the disease/explaining my illness to my family and/or friends | 🞎 |
| 7 | It is difficult for me to ask my family and/or friends for help | 🞎 |
| 8 | Since I've been sick, I've limited social/friendly relationships | 🞎 |
| 9 | Other difficulties in family or friend life related to the disease, please specify............................................................... | 🞎 |
| 10 | I am not affected by any difficulties in family or friendship life that would be related to the disease | 🞊 |

**Q44B**. [if ≠10 in Q44] Have you been able to discuss your difficulties in your family and/or friendship life?

*Only one possible answer per column*

|  |  | With a health professional in the hospital or clinic who is following you for your cancer | With a healthcare professional outside the hospital or clinic where you are being treated for your cancer |
| --- | --- | --- | --- |
| **1** | Yes and he/she was very helpful | 🞊 | 🞊 |
| **2** | Yes, but he/she has not been particularly helpful | 🞊 | 🞊 |
| **3** | No, but I don't want to talk about this type of subject | 🞊 | 🞊 |
| **4** | No, but I would have liked to have been able to address this type of subject | 🞊 | 🞊 |

**Q44C.** [if ≠ 10 in Q44] How much is your home life affected by the disease?

**Please give a score from 1 to 10.**

*1 = my family life is not affected by the disease at all*

*10 = My family life is very affected by the disease*

*Intermediate grades allow you to modulate your judgment*

| ***1***  💣 | ***2***  💣 | ***3***  💣 | ***4***  💣 | ***5***  💣 | ***6***  💣 | ***7***  💣 | ***8***  💣 | ***9***  💣 | ***10***  💣 |
| --- | --- | --- | --- | --- | --- | --- | --- | --- | --- |

***Not affected at all	Is very affected***

E. Pregnancy/child project

**Q45.** Since you have ALK+ lung cancer, have you had a pregnancy/child project?

| 1 | Yes, and we were able to have a child | 🞊 |
| --- | --- | --- |
| 2 | Yes, but without success for the moment | 🞊 |
| 3 | Yes, but we gave up on having a child because of the disease | 🞊 |
| 4 | Yes and we have decided to adopt a child | 🞊 |
| 5 | No | 🞊 |

**Q45B**. [if ≠not in Q45] Have you been able to discuss your pregnancy/child project with the medical team that is following you for your cancer?

*Only one answer possible*

| **1** | Yes and she was very helpful | 🞊 |
| --- | --- | --- |
| **2** | Yes, but she wasn't particularly helpful | 🞊 |
| **3** | Yes, but she advised us not to embark on this project of having a child | 🞊 |
| **4** | No | 🞊 |

**Q45C.** [if ≠not in Q45] Has the medical team offered a preventive treatment to preserve your fertility or that of your loved one?

| 1 | Yes | 🞊 |
| --- | --- | --- |
| 2 | No | 🞊 |

F. Moral, Psychological well-being

**Q46.** Could you indicate if you are affected by some of these psychological difficulties?

**Please select only the difficulties that you attribute to your illness**

*Several possible answers*

| 1 | Feeling angry, unfair, unfair, | 🞎 |
| --- | --- | --- |
| 2 | Depressive episodes | 🞎 |
| 3 | Episodes of sadness | 🞎 |
| 4 | Guilt, feeling like a burden to those around me | 🞎 |
| 5 | Feeling insecure, afraid of tomorrow | 🞎 |
| 6 | Feeling excluded, of being alone | 🞎 |
| 7 | Loss of self-confidence | 🞎 |
| 8 | Mood disorders |  |
| 9 | Anxiety, Anguish | 🞎 |
| 10 | Dark thoughts | 🞎 |
| 11 | Other psychological difficulties related to the disease, please specify............................................................... | 🞎 |
| 12 | I am not affected by any psychological difficulty that would be related to the disease | 🞊 |

**Q46B**. [if ≠12 in Q46] Were you able to discuss your psychological difficulties?

*Only one possible answer per column*

|  |  | With a health professional in the hospital or clinic who is following you for your cancer | With a healthcare professional outside the hospital or clinic where you are being treated for your cancer |
| --- | --- | --- | --- |
| **1** | Yes and he/she was very helpful | 🞊 | 🞊 |
| **2** | Yes, but he/she has not been particularly helpful | 🞊 | 🞊 |
| **3** | No, but I don't want to talk about this type of subject | 🞊 | 🞊 |
| **4** | No, but I would have liked to have been able to address this type of subject | 🞊 | 🞊 |

**Q46C.** [if ≠ code 12 in Q46] To what extent do these psychological difficulties related to the disease affect your daily life?

**Please give a score from 1 to 10.**

*1 = my daily life is not affected at all by these difficulties*

*10 = My daily life is very affected by these difficulties*

*Intermediate grades allow you to modulate your judgment*

| ***1***  💣 | ***2***  💣 | ***3***  💣 | ***4***  💣 | ***5***  💣 | ***6***  💣 | ***7***  💣 | ***8***  💣 | ***9***  💣 | ***10***  💣 |
| --- | --- | --- | --- | --- | --- | --- | --- | --- | --- |

***Not affected at all	Is very affected***

**Q46D.** Would you say today, that in the face of this disease you feel ...

Please select a maximum of 4 adjectives that best fit you from the list below

| ***1*** | 🞎 |  | Optimistic | | ***6*** | 🞎 |  | Confident | | | ***11*** | 🞎 | Surrounded/listened to |
| --- | --- | --- | --- | --- | --- | --- | --- | --- | --- | --- | --- | --- | --- |
| ***2*** | 🞎 |  | Resigned | | ***7*** | 🞎 |  | Active/Active | | | ***12*** | 🞎 | Angry |
| ***3*** | 🞎 |  | Discouraged | | ***8*** | 🞎 |  | Fragile | | | ***13*** | 🞎 | Lucid |
| ***4*** | 🞎 |  | Combative | | ***9*** | 🞎 |  | Solid | | | ***14*** | 🞎 | Pessimist |
| ***5*** | 🞎 |  | Anxious | | ***10*** | 🞎 |  | Isolated | | | ***15*** | 🞎 | Powerless |
|  |  |  | |  |  |  | | |  |  |  |  |  |

G. Financial angle

**Q47.** Could you indicate whether you are affected by some of these financial difficulties?

**Please select only the difficulties that you attribute to your ALK+ lung cancer**

*Several possible answers*

| 1 | I have experienced a drop in income in the context of my professional activity (loss of a job, part-time work, change of profession, disability,...) | 🞎 |
| --- | --- | --- |
| 2 | My spouse has experienced a drop in income in the context of his/her professional activity (loss of a job, transition to part-time work, change of profession) | 🞎 |
| 3 | I had difficulty obtaining a loan because of my illness | 🞎 |
| 4 | A loan was refused because of my illness | 🞎 |
| 5 | I lack/have long lacked information on possible social assistance | 🞎 |
| 6 | The social assistance I receive is insufficient in my opinion | 🞎 |
| 7 | I encountered difficulties in receiving my allowances from my pension scheme | 🞎 |
| 8 | Other financial difficulties related to the disease, please specify............................................................... | 🞎 |
| 9 | I am not affected by any financial difficulties that would be related to the disease | 🞊 |

**Q47B.** [if ≠9 in Q47] Were you able to discuss your financial difficulties?

*Only one possible answer per column*

|  |  | With a health professional in the hospital or clinic who is following you for your cancer | With a healthcare professional outside the hospital or clinic where you are being treated for your cancer |
| --- | --- | --- | --- |
| **1** | Yes and he/she was very helpful | 🞊 | 🞊 |
| **2** | Yes, but he/she has not been particularly helpful | 🞊 | 🞊 |
| **3** | No, but I don't want to talk about this type of subject | 🞊 | 🞊 |
| **4** | No, but I would have liked to have been able to address this type of subject | 🞊 | 🞊 |

**Q47C.** [if ≠ code 9 in Q47] To what extent do these financial difficulties related to the disease affect your daily life?

**Please give a score from 1 to 10.**

*1 = My daily life/quality of life is not affected at all by these difficulties*

*10 = My daily life/quality of life is very affected by these difficulties*

*Intermediate grades allow you to modulate your judgment*

| ***1***  💣 | ***2***  💣 | ***3***  💣 | ***4***  💣 | ***5***  💣 | ***6***  💣 | ***7***  💣 | ***8***  💣 | ***9***  💣 | ***10***  💣 |
| --- | --- | --- | --- | --- | --- | --- | --- | --- | --- |

***Not affected at all***	***Is very affected***

H. Life projects

**Q48.** Could you indicate whether you are affected by some of these difficulties in the implementation of your projects?

**Please select only the difficulties that you attribute to your ALK+ lung cancer**

*Several possible answers*

| 1 | I have difficulty projecting myself into the future | 🞎 |
| --- | --- | --- |
| 2 | I have difficulty setting up projects, planning | 🞎 |
| 3 | I don't feel able to carry out my projects since I'm sick | 🞎 |
| 4 | I had to give up some sports activities | 🞎 |
| 5 | I had to give up some cultural and artistic activities | 🞎 |
| 6 | I had to give up organizing trips, vacation plans | 🞎 |
| 7 | Other difficulties related to my life projects related to the disease, please specify............................................................... | 🞎 |
| 8 | I am not concerned by any difficulty in making plans for those related to the disease | 🞊 |

**Q48B**. [if ≠8 in Q48] Were you able to discuss your difficulties in carrying out your projects?

*Only one possible answer per column*

|  |  | With a health professional in the hospital or clinic who is following you for your cancer | With a healthcare professional outside the hospital or clinic where you are being treated for your cancer |
| --- | --- | --- | --- |
| **1** | Yes and he/she was very helpful | 🞊 | 🞊 |
| **2** | Yes, but he/she has not been particularly helpful | 🞊 | 🞊 |
| **3** | No, but I don't want to talk about this type of subject | 🞊 | 🞊 |
| **4** | No, but I would have liked to have been able to address this type of subject | 🞊 | 🞊 |

**Q48C.** [if ≠8 in Q48] To what extent do these difficulties in carrying out your projects affect your daily life?

**Please give a score from 1 to 10.**

*1 = My daily life/quality of life is not affected at all by these difficulties*

*10 = My daily life/quality of life is very affected by these difficulties*

*Intermediate grades allow you to modulate your judgment*

| ***1***  💣 | ***2***  💣 | ***3***  💣 | ***4***  💣 | ***5***  💣 | ***6***  💣 | ***7***  💣 | ***8***  💣 | ***9***  💣 | ***10***  💣 |
| --- | --- | --- | --- | --- | --- | --- | --- | --- | --- |

***Not affected at all	Is very affected***

**Q49.** In summary, among all these difficulties caused by the disease, please select the 3 that affect your daily life the most?

Please rank in hierarchical order and rate 1 for the difficulty that affects your daily life the most, then 2 for the difficulty you rank in 2nd position, then 3 for the third

| 1 | [if ≠9 in Q40] Physical difficulties in performing daily life | /____/ |
| --- | --- | --- |
| 2 | [if ≠15 or 16 in Q42] Career difficulties | /____/ |
| 3 | [if ≠5 Q41] Difficulties in school/student life | /____/ |
| 4 | [if ≠9 or 10 in Q43] Difficulties in the life of a couple, intimate life | /____/ |
| 5 | [if ≠9 in Q44] Difficulties in family life, friendships | /____/ |
| 6 | [if ≠5 in Q45] Difficulties related to your pregnancy/child plans | /____/ |
| 7 | [if ≠12 in Q46] Psychological, moral difficulties | /____/ |
| 8 | [if ≠9 in Q47] Financial Hardship | /____/ |
| 9 | [if ≠8 in Q48] Difficulties in achieving your life projects | /____/ |

[prog instruction: if less than 3 groups of difficulties mentioned from Q40 to Q48] please select the one that affects your daily life the most?

**Q56.** Finally, here are some different actions/initiatives that could be put in place to support patients with ALK+ cancer. How important do you think they are to put in place?

**Please give a score from 1 to 10.**

*1 = action not at all important to put in place according to you*

*10 = very important action to put in place according to you*

*Intermediate grades allow you to modulate your judgment*

| 1 | Allows me to report the side effects of my treatment more easily to the medical team that follows me | /____/ |
| --- | --- | --- |
| 2 | More regular assessments by the medical team of my supportive care needs | /____/ |
| 3 | Not having to go to the hospital to receive supportive care through external structures that would be closer to my home | /____/ |
| 4 | Tools to work on my memory, my concentration, my attention | /____/ |
| 5 | To allow me to access information on returning/accessing work after cancer | /____/ |
| 6 | A more systematic proposal of activities for my well-being (yoga, meditation, sophrology, shiatsu, adapted physical activity, ...) | /____/ |
| 7 | To allow me to exchange with other patients to break my isolation in the face of the disease within local support groups | /____/ |
| 8 | Offer me psychological support to live better with my disease | /____/ |
| 9 | Set up a "Quality of Life" consultation in the day hospital for patients and their caregivers which would be dedicated to the non-medical difficulties encountered (social, family, professional, etc.) | /____/ |

**Signage [1 to 2']**

Finally, thank you for answering these few socio-demographic questions

**S11.** May I ask you your age please?

*Please indicate your age below*

/________/Years

**S12.** You are...

*Only one answer possible*

| 1 | A man | 🞊 |
| --- | --- | --- |
| 2 | A woman | 🞊 |
| 3 | I don't want to answer | 🞊 |

**S13.** Do you have children?

*Only one answer possible*

| 1 | Yes | 🞊 |
| --- | --- | --- |
| 2 | No | 🞊 |

**S14. [if yes in S13]** Your child has / your children have ...

*Several possible answers*

| 1 | Under 10 years old | 🞎 | to Number /___/ |
| --- | --- | --- | --- |
| 2 | Between 10 and 17 years old | 🞎 | Number/___/ |
| 3 | 18 years and older | 🞎 | Number/___/ |

**S15.** Within your home, do you live ...

*Only one answer possible*

| 1 | Alone, with children | 🞊 |
| --- | --- | --- |
| 2 | Alone without children | 🞊 |
| 3 | As a couple, with children | 🞊 |
| 4 | As a couple, without children | 🞊 |
| 5 | Alone, with parents or one of parents / siblings | 🞊 |

**S16.** What is (or was) your profession...

*If you are not currently working or no longer working but have already worked, please indicate your former profession here / only one answer possible*

| 1 | Farmer, operator (self-employed) |  | 🞊 |
| --- | --- | --- | --- |
| 2 | Craftsman, shopkeeper |  | 🞊 |
| 3 | Business Manager |  | 🞊 |
| 4 | Executive and other higher intellectual profession |  | 🞊 |
| 5 | Liberal professions (except paramedics) |  | 🞊 |
| 6 | Professor, scientific profession |  | 🞊 |
| 7 | Foreman, supervisor, paramedical profession, technician |  | 🞊 |
| 8 | Primary school teacher or equivalent |  | 🞊 |
| 9 | Employee |  | 🞊 |
| 10 | Service staff |  | 🞊 |
| 11 | Worker |  | 🞊 |
| 12 | Student |  | 🞊 |
| 13 | No profession |  | 🞊 |

**S17.** Living...

*Only one answer possible*

| 1 | In a large metropolis: more than 100,000 inhabitants | 🞊 |
| --- | --- | --- |
| 2 | In a large city: 50,000 to 99,999 inhabitants | 🞊 |
| 3 | In a medium-sized city: from 10,000 to 49,999 inhabitants | 🞊 |
| 4 | In a small town: 2000 to 9,999 inhabitants | 🞊 |
| 5 | In rural areas: less than 2000 inhabitants | 🞊 |

**S18.** On average, how long does it take you to get to the hospital where you are being treated for your ALK+ lung cancer?

*Only one answer possible*

| 1 | Less than 30 minutes of travel | 🞊 |
| --- | --- | --- |
| 2 | Between 30 minutes and less than 1 hour journey | 🞊 |
| 3 | About 1 hour journey | 🞊 |
| 4 | Between 1 and 2 hours of travel | 🞊 |
| 5 | More than 2 hours of travel | 🞊 |

**S18B.** What means of transport do you use most often when you travel to the hospital?

*Only one answer possible*

| 1 | Public transport | 🞊 |
| --- | --- | --- |
| 2 | My car | 🞊 |
| 3 | My loved one's car | 🞊 |
| 4 | Taxi or VSL | 🞊 |
| 5 | Others, please specify /_____________________/ | 🞊 |
| 6 | [if helping] I don't take care of my sick relative's travel, he/she manages his/her movements alone | 🞊 |

**S19.** What is your level of education (highest degree you have obtained)?

*Only one answer possible*

| 1 | Up to primary education | 🞊 |
| --- | --- | --- |
| 2 | Until secondary education without the baccalaureate | 🞊 |
| 3 | Baccalaureate without post-baccalaureate studies | 🞊 |
| 4 | Baccalaureate + 2 years of post-baccalaureate studies | 🞊 |
| 5 | Baccalaureate + 3 years of post-baccalaureate studies | 🞊 |
| 6 | Baccalaureate + 4 years of post-baccalaureate studies and more | 🞊 |

**S20.** Of the following brackets, what is your net monthly household income (on average)?

*Please count all the resources of your household: income, family allowances and other financial resources of all members of the household / Please check only one answer*

*Only one answer possible*

| 1 | Under €499 | 🞊 |
| --- | --- | --- |
| 2 | From 500 to 1,499 € | 🞊 |
| 3 | From €1,500 to €2,499 | 🞊 |
| 4 | From €2,500 to €3,499 | 🞊 |
| 5 | From €3,500 to €4,499 | 🞊 |
| 6 | From €4,500 to €5,499 | 🞊 |
| 7 | From €5,500 to €6,499 | 🞊 |
| 8 | From €6,500 to €7,499 | 🞊 |
| 9 | 7500 € and more | 🞊 |
| 10 | Don't want to answer | 🞊 |

**S20B. Do** you subscribe to a mutual or complementary health insurance company?

*Only one answer possible*

| 1 | Yes, I subscribe to a mutual insurance company | 🞊 |
| --- | --- | --- |
| 2 | Yes, I subscribe to a complementary health insurance (e.g. CMU-C) | 🞊 |
| 3 | No | 🞊 |

**S20C.** Have you, in the context of your lung cancer, obtained recognition of your disability through procedures with the MDPH (Departmental House for Disabled People)?

*Only one answer possible*

| **1** | Yes and I am still currently on disability | 🞊 |
| --- | --- | --- |
| **2** | Yes, but I am no longer on disability | 🞊 |
| **2** | No | 🞊 |

**S20D.** [If code 1 in S20C], How long have you been using it? /___/ month or /___/ years

**S20E.** [If code 2 in S20C], How long did you benefit from it? /___/ month or /___/ years

**S21.** Which of the following aids do you receive or have been able to receive in the context of your ALK+ lung cancer?

*Several possible answers except last line*

| 1 | Disability allowance | □ |
| --- | --- | --- |
| 2 | RQTH (recognition of the status of disabled worker) | □ |
| 3 | ALD (100% coverage - Long-term condition) | □ |
| 4 | Home Helper | □ |
| 5 | Life assistant | □ |
| 4 | I am not receiving any of these subsidies | 🞊 |

**S22**. Do you know of one or more associations involved in supporting and accompanying cancer patients?

*Only one answer possible*

| **1** | Yes | 🞊 |
| --- | --- | --- |
| **2** | No | 🞊 |

**S22B.** [If yes in S22] What associations do you know?

**S22C.** [If yes in S22] How did you find out about this patient association(s)?

*Several possible answers*

| **1** | Via my oncologist | □ |
| --- | --- | --- |
| **2** | Via another member of the medical team | □ |
| **3** | Via my GP | □ |
| **4** | Through my own personal research | □ |
| **5** | Via my relatives | □ |
| **6** | Via other patients | □ |

**This questionnaire is now closed, thank you for your participation!**
