# Supplementary material for: Living With ALK Fusion-Positive NSCLC: A French Nationwide Survey of the Psychosocial Impact Among Patients, Caregivers, and Health Care Professionals
Source: JTO Clin Res Rep. 2026 Jun 30;7(10):101038. doi: 10.1016/j.jtocrr.2026.101038 (PMC13626941; doi:10.1016/j.jtocrr.2026.101038)
Supplement: Supplementary file 3 [file mmc3.docx]

**Appendix B**

**National Survey of Patients and Their Caregivers:**

**ALK+ lung cancer – Lived experience, needs and expectations in the face of this disease**

**CARERS QUESTIONNAIRE [do not display – code 2 in S0]**

**Part A. [if patient in L1] The time of diagnosis [3-5']**

**[if patient in L2 and +] The time of initial diagnosis and relapse [4-6']**

**Patients in L1** [= code 1 in S6B or code 2 in S6C] [do not display]

**Let's start with your loved one's ALK+ lung cancer diagnosis.**

**Q1.** How old was your loved one when he was diagnosed with ALK+ lung cancer?

/__/__/Years

**Q1B.** Were you present with your loved one when this diagnosis was announced?

*Only one answer possible*

| 1 | Yes | 🞊 |  |
| --- | --- | --- | --- |
| 2 | No | 🞊 |  |

**Q1C.** Did a doctor or other member of the healthcare team subsequently provide you – as a loved one – with information about the diagnosis of their disease and/or its treatment?

*Only one answer possible*

| 1 | Yes | 🞊 |  |
| --- | --- | --- | --- |
| 2 | No | 🞊 |  |

**Q6.** [if yes in Q1B or Q1C] Has this announcement of the diagnosis been the subject of...

|  | *Only one possible answer per line* | Yes | No | I don't remember |
| --- | --- | --- | --- | --- |
| 1 | A specific consultation / an announcement consultation with yourself as a relative (with or without your sick relative) | 🞊 | 🞊 | 🞊 |
| 2 | An appointment with a nurse to announce yourself as a relative (with or without your sick relative) on the same day, or a few days later | 🞊 | 🞊 | 🞊 |

**Q8.** [to all] Would you say that at this moment of diagnosis, you, as a loved one, felt **taken into consideration** by the care team?

*Only one answer possible*

| 1 | Yes, absolutely | 🞊 |
| --- | --- | --- |
| 2 | Yes, partially | 🞊 |
| 3 | No | 🞊 |
| 4 | I don't remember | 🞊 |

**Q9.** [if yes in Q1B or Q1C] Always when the diagnosis is announced and before your loved one starts treatment, **have you found** that **the information that has been brought to you by the care team to better understand** ...

|  | *Only one possible answer per line* | **Were sufficient** | **Were insufficient** | **I don't remember** |
| --- | --- | --- | --- | --- |
| 1 | ALK+ lung cancer | 🞊 | 🞊 | 🞊 |
| 2 | The treatment implemented | 🞊 | 🞊 | 🞊 |

**Q10.** [to all] Would you say that at this point in the diagnosis and before your loved one started their treatment, you understood...

|  | *Only one possible answer per line* | **Yes, absolutely** | **Yes, partially** | **No** |
| --- | --- | --- | --- | --- |
| 1 | The particularities of ALK+ lung cancer | 🞊 | 🞊 | 🞊 |
| 2 | That this disease carries a risk of relapse | 🞊 | 🞊 | 🞊 |
| 3 | The reasons for choosing the treatment protocol for your loved one | 🞊 | 🞊 | 🞊 |
| 4 | How its treatment works | 🞊 | 🞊 | 🞊 |
| 5 | The benefits your loved one can expect from their treatment | 🞊 | 🞊 | 🞊 |
| 6 | Side effects your loved one may experience during treatment | 🞊 | 🞊 | 🞊 |
| 7 | That your loved one should take their treatment chronically (i.e. every day) without stopping it even if he/she feels better | 🞊 | 🞊 | 🞊 |

**Q14.** [to all] Have you searched **for additional information on your own**, following the diagnosis...

|  | *Only one response per line* | **Yes** | **No** |
| --- | --- | --- | --- |
| 1 | About your loved one's ALK+ lung cancer | 🞊 | 🞊 |
| 2 | On the treatment put in place | 🞊 | 🞊 |
| 3 | On the different supportive care that can be offered | 🞊 | 🞊 |
| 4 | On patient associations | 🞊 | 🞊 |

**Q14B.** [If ≥ 1 yes answer in Q14] How did you look for this information?

*Several possible answers*

| 1 | Via personal research on the internet | 🞎 |
| --- | --- | --- |
| 2 | Via patient associations | 🞎 |
| 3 | Via health professionals | 🞎 |
| 4 | Via the attending physician | 🞎 |
| 5 | Others, please specify....................................... | 🞎 |

***Note on supportive care:*** *supportive care is all the care and support offered to sick people throughout the disease (access to a dietician, a pain specialist, adapted physical activity, a psychologist, etc.)*

**Patients in L2+** [= code 2 or 3 in S6B or code 1 in S6C] [do not display]

**Let's start by talking about when your loved one was diagnosed with ALK+ lung cancer for the first time.**

**Q1A.** How old was your loved one when the 1st diagnosis of his ALK+ lung cancer was made?

/__/__/Years

**Q1A.1** Were you present with your loved one when this 1st diagnosis was announced?

*Only one answer possible*

| 1 | Yes | 🞊 |  |
| --- | --- | --- | --- |
| 2 | No | 🞊 |  |

**Q1A.2** Has a doctor or other member of the health care team subsequently provided you as a relative with information about the diagnosis of their illness and/or treatment?

*Only one answer possible*

| 1 | Yes | 🞊 |  |
| --- | --- | --- | --- |
| 2 | No | 🞊 |  |

**Q6A.** [if yes in Q1A.1 or Q1A.2] Has this announcement of the diagnosis been the subject of...

|  | *Only one possible answer per line* | Yes | No | I don't remember |
| --- | --- | --- | --- | --- |
| 1 | A specific consultation / an announcement consultation with yourself as a relative (with or without your sick relative) | 🞊 | 🞊 | 🞊 |
| 2 | An appointment with a nurse to announce yourself as a relative (with or without your sick relative) on the same day, or a few days later | 🞊 | 🞊 | 🞊 |

**Q8A.** [to all] Would you say that at this moment of diagnosis, you yourself, as a loved one, felt **taken into consideration** by the care team?

*Only one answer possible*

| 1 | Yes, absolutely | 🞊 |
| --- | --- | --- |
| 2 | Yes, partially | 🞊 |
| 3 | No | 🞊 |
| 4 | I don't remember | 🞊 |

**Q9A.** [if yes in Q2A or Q3A] Still at this time of the 1st diagnosis and before your loved one starts treatment, **have you found** that **the information that has been brought to you by the care team to better understand** ...

|  | *Only one possible answer per line* | **Were sufficient** | **Were insufficient** | **I don't remember** |
| --- | --- | --- | --- | --- |
| 1 | ALK+ lung cancer | 🞊 | 🞊 | 🞊 |
| 2 | The treatment implemented | 🞊 | 🞊 | 🞊 |

**Q10A.** [to all] Would you say that at this point in the 1st diagnosis and before your loved one starts their treatment, you have understood...

|  | *Only one possible answer per line* | **Yes, absolutely** | **Yes, partially** | **No** |
| --- | --- | --- | --- | --- |
| 1 | The particularities of ALK+ lung cancer | 🞊 | 🞊 | 🞊 |
| 2 | That this disease carries a risk of relapse | 🞊 | 🞊 | 🞊 |
| 3 | The reasons for choosing the treatment protocol for your loved one | 🞊 | 🞊 | 🞊 |
| 4 | How its treatment works | 🞊 | 🞊 | 🞊 |
| 5 | The benefits your loved one can expect from their treatment | 🞊 | 🞊 | 🞊 |
| 6 | Side effects your loved one may experience during treatment | 🞊 | 🞊 | 🞊 |
| 7 | That your loved one should take their treatment chronically (i.e. every day) without stopping it even if he/she feels better | 🞊 | 🞊 | 🞊 |

**Q14A.** [to all] Have you searched **for additional information on your own**, following the 1st diagnosis...

|  | *Only one response per line* | **Yes** | **No** |
| --- | --- | --- | --- |
| 1 | About your loved one's ALK+ lung cancer | 🞊 | 🞊 |
| 2 | On the treatment protocol put in place | 🞊 | 🞊 |
| 3 | On the different supportive care Supportive care that can be offered | 🞊 | 🞊 |
| 4 | On patient associations | 🞊 | 🞊 |

***Note on supportive care:*** *supportive care is all the care and support offered to sick people throughout the disease (access to a dietician, a pain specialist, adapted physical activity, a psychologist, etc.)*

**Q14B2.** [If ≥ 1 yes answer in Q14] How did you look for this information?

*Several possible answers*

| 1 | Via personal research on the internet | 🞎 |
| --- | --- | --- |
| 2 | Via patient associations | 🞎 |
| 3 | Via health professionals | 🞎 |
| 4 | Via the attending physician | 🞎 |
| 5 | Others, please specify....................................... | 🞎 |

**Now let's talk about the moment of the relapse/progression of the disease experienced by your loved one. If your loved one has experienced multiple relapses, we will talk about their last relapse here.**

**Q15.** How old was your loved one when he was diagnosed with ALK+ lung cancer relapse?

/__/__/Years

**Q15B.** Were you present with your loved one when his or her relapse was announced?

*Only one answer possible*

| 1 | Yes | 🞊 |  |
| --- | --- | --- | --- |
| 2 | No | 🞊 |  |

**Q15C.** Did a doctor or other member of the healthcare team subsequently provide you - as a loved one - with information about their relapse and the new treatment to be implemented?

*Only one answer possible*

| 1 | Yes | 🞊 |  |
| --- | --- | --- | --- |
| 2 | No | 🞊 |  |

**Q18.** [if yes in Q15 or Q16] Was this moment of the relapse the subject of the relapse...

|  | *Only one possible answer per line* | Yes | No | I don't remember |
| --- | --- | --- | --- | --- |
| 1 | A specific consultation / an announcement consultation with yourself as a relative (with or without your sick relative) | 🞊 | 🞊 | 🞊 |
| 2 | An appointment with a nurse to announce yourself as a relative (with or without your sick relative) on the same day, or a few days later | 🞊 | 🞊 | 🞊 |

**Q20.** [to all] Would you say that at this point in the relapse, you yourself as a loved one, **you felt taken into consideration** by the care team?

*Only one answer possible*

| 1 | Yes, absolutely | 🞊 |
| --- | --- | --- |
| 2 | Yes, partially | 🞊 |
| 3 | No | 🞊 |
| 4 | I don't remember | 🞊 |

**Q21.** [if yes in Q15 or Q16] Still at this point in the relapse and before your loved one starts their new treatment, have you found that **the information that has been brought to you to better understand** ...

*Only one possible answer per line*

|  |  | **Were sufficient** | **Were insufficient** | **I don't remember** |
| --- | --- | --- | --- | --- |
| 1 | The progression of his disease | 🞊 | 🞊 | 🞊 |
| 2 | The new treatment protocol put in place for your loved one | 🞊 | 🞊 | 🞊 |

**Q22.** [to all] Would you say that at the time of the relapse and before your loved one started their new treatment, you understood ...

|  | *Only one possible answer per line* | **Yes, absolutely** | **Yes, partially** | **No** |
| --- | --- | --- | --- | --- |
| 1 | The reasons for choosing the new treatment protocol implemented | 🞊 | 🞊 | 🞊 |
| 2 | How does his new treatment work? | 🞊 | 🞊 | 🞊 |
| 3 | The benefits your loved one can expect from their new treatment | 🞊 | 🞊 | 🞊 |
| 4 | Side effects your loved one may experience during their new treatment | 🞊 | 🞊 | 🞊 |

**Q26.** [to all] Have you searched **for additional** information on your own, following your loved one's relapse...

|  | *Only one response per line* | **Yes** | **No** |
| --- | --- | --- | --- |
| 1 | The progression of his disease | 🞊 | 🞊 |
| 2 | The new treatment protocol that will be put in place for your loved one | 🞊 | 🞊 |
| 3 | On the different supportive care Supportive care that can be offered | 🞊 | 🞊 |
| 4 | On patient associations | 🞊 | 🞊 |

***Note on supportive care:*** *supportive care is all the care and support offered to sick people throughout the disease (access to a dietician, a pain specialist, adapted physical activity, a psychologist, etc.)*

**Q26B.** [If ≥ 1 yes answer in Q14] How did you look for this information?

*Several possible answers*

| 1 | Via personal research on the internet | 🞎 |
| --- | --- | --- |
| 2 | Via patient associations | 🞎 |
| 3 | Via health professionals | 🞎 |
| 4 | Via the attending physician | 🞎 |
| 5 | Others, please specify....................................... | 🞎 |

**Part B. ALK+ lung cancer treatment and its perception [7-8']**

**Now let's talk about the current targeted therapy treatment your loved one is receiving and their medical follow-up.**

**Q27B.** When did your loved one start this targeted therapy treatment that he/she is currently taking ?

/__/__/ /__/__/__/__/ (please enter the two digits of the month and then the year – if you don't remember the month, you can just enter the year)

🞊 I don't know

**Q28.** [for L2+ patients] What treatment(s) has your loved one received in the past to treat their ALK+ lung cancer?

*Several possible answers*

| 1 | Chemotherapy | 🞎 |
| --- | --- | --- |
| 2 | Targeted therapy | 🞎 |
| 3 | Radiotherapy | 🞎 |
| 4 | Immunotherapy | 🞎 |
| 5 | Surgery | 🞎 |
| 6 | Treatment in a clinical trial | 🞎 |
| 7 | Others, please specify /_______________/ | 🞎 |
| 8 | I don't remember | 🞎 |

**Q29.** Do you feel that you are, today**,** about the targeted therapy treatment that your loved one is receiving...

*Only one answer possible*

| 1 | Very knowledgeable | 🞊 |
| --- | --- | --- |
| 2 | Fairly knowledgeable | 🞊 |
| 3 | Somewhat uninformed | 🞊 |
| 4 | Very uninformed | 🞊 |

**Q31.** Here are some criteria that can be associated with treatment. For each of these criteria, how would you rate each of these treatments in relation to the image you have of them?

*1, means that you do not attribute this criterion to the corresponding treatment at all. 10, that you strongly attribute this criterion to the corresponding treatment. Intermediate grades allow you to modulate your judgment.*

*[programming: cursor to be provided]*

|  |  | **Targeted therapy** | **Chemotherapy** |
| --- | --- | --- | --- |
| 1 | Quality of life preserved with this treatment | \|___\| 🞊NSP | \|___\|¤NSP |
| 2 | Favorable balance between benefits and constraints with this treatment | \|___\| 🞊NSP | \|___\|¤NSP |
| 3 | Tolerable side effects | \|___\| 🞊NSP | \|___\|¤NSP |
| 4 | Treatment that gives hope | \|___\| 🞊NSP | \|___\|¤NSP |
| 5 | Ease of administration of this treatment | \|___\| 🞊NSP | \|___\|¤NSP |
| 6 | Treatment well monitored by the medical team | \|___\| 🞊NSP | \|___\|¤NSP |
| 7 | Treatment that allows you to live almost normally | \|___\| 🞊NSP | \|___\|¤NSP |
| 8 | Treatment with long-lasting effectiveness | \|___\| 🞊NSP | \|___\|¤NSP |

**Q32.** Today, how often does your loved one see the doctor who follows him/her for his/her treatment with targeted therapy?

All |___| month

**Q32A.** Are you present during these consultations?

*Only one answer possible*

| 1 | Systematically | 🞊 |
| --- | --- | --- |
| 2 | Often | 🞊 |
| 3 | From time to time | 🞊 |
| 4 | Rarely | 🞊 |
| 5 | Never | 🞊 |

**Q33**. [if ≠ 4 or 5 in Q32A] How long do these follow-up visits last, on average?

|___| Minutes

**Q34**. Has telephone support been set up to monitor your loved one's current treatment? (Regular phone calls from a nurse at their care facility)

*Only one answer possible*

| 1 | Yes | 🞊 |
| --- | --- | --- |
| 2 | No | 🞊 |

**Q34A**. [If not in Q34] Would you have liked a telephone support to be set up for the follow-up of his current treatment?

*Only one answer possible*

| 1 | Yes | 🞊 |
| --- | --- | --- |
| 2 | No | 🞊 |

**Q35.** Here are some statements about tracking your loved one's illness and their current treatment. For each of them, please indicate if you agree by checking the box that best corresponds to how you feel.

| *Only one response per line* | | Strongly disagree | Rather disagree | Tend to agree | Totally agree |
| --- | --- | --- | --- | --- | --- |
| 1 | The duration of the follow-up consultations is, as a general rule, perceived as sufficient by my loved one | 🞊 | 🞊 | 🞊 | 🞊 |
| 2 | My loved one does not miss any follow-up medical appointments because being monitored regularly reassures him or her | 🞊 | 🞊 | 🞊 | 🞊 |
| 3 | The time between the results of the CT scan and the consultation with the oncologist is often too long and a source of anxiety and stress for my loved one | 🞊 | 🞊 | 🞊 | 🞊 |
| 4 | My loved one always experiences the check-ups with great concern | 🞊 | 🞊 | 🞊 | 🞊 |
| 5 | [if yes in Q34] The telephone support allowed my loved one to better understand his/her treatment | 🞊 | 🞊 | 🞊 | 🞊 |
| 6 | [if yes in Q34] The telephone support allowed my loved one to better manage the side effects of his treatment | 🞊 | 🞊 | 🞊 | 🞊 |
| 7 | As soon as my loved one feels better, he/she wants to stop taking his/her medication | 🞊 | 🞊 | 🞊 | 🞊 |
| 8 | My loved one sometimes feels that his or her treatment is doing more harm than good | 🞊 | 🞊 | 🞊 | 🞊 |

**Q35B.** As a loved one of a patient, would you say that you are **listened to, taken into consideration** by the health professionals who take care of your loved one?

*You have scores from 1 to 10 to answer this question. 1, means that you do not feel listened to/taken into consideration at all; 10, means that you feel strongly listened to/taken into consideration. Intermediate grades allow you to modulate your judgment*

| ***1*** 💣 \| ***2*** 💣 \| ***3*** 💣 \| ***4*** 💣 \| ***5*** 💣 \| ***6*** 💣 \| ***7*** 💣 \| ***8*** 💣 \| ***9*** 💣 \| ***10*** 💣 | | |
| --- | --- | --- |
| ***Not listened to/taken into consideration at all*** | ***Strongly listened to/taken into consideration*** |  |

**Q37.** Who are all of the following professionals who are currently following your loved one for ALK+ lung cancer? *Several possible answers*

**Q37B.** And who are the ones who provide the most support in the face of the repercussions of the disease on their daily life? *Several possible answers*

**Q37C.** And which ones provide you as a loved one with the most support in the face of the concrete repercussions of the disease on your daily life? *Several possible answers*

|  |  | **All current stakeholders in the cancer care of my loved one** | **Those who provide the most support to my loved one** [filter Q33] | **Those who provide me as a loved one with the most support** |
| --- | --- | --- | --- | --- |
| 1 | Oncologist | 🞎 | 🞎 | 🞎 |
| 2 | Pulmonologist | 🞎 | 🞎 | 🞎 |
| 3 | Internist | 🞎 | 🞎 | 🞎 |
| ~~4~~ | Radiotherapist | ~~🞎~~ | ~~🞎~~ | ~~🞎~~ |
| 5 | General practitioner / general practitioner | 🞎 | 🞎 | 🞎 |
| 6 | Surgeon | 🞎 | 🞎 | 🞎 |
| 7 | Psychologist, psychiatrist | 🞎 | 🞎 | 🞎 |
| 8 | Health Facilitator (ERI Meeting and Information Space) | 🞎 | 🞎 | 🞎 |
| 9 | Hospital Nurse, Clinic Nurse | 🞎 | 🞎 | 🞎 |
| 10 | Advanced Practice Nurse (APN) | 🞎 | 🞎 | 🞎 |
| 11 | Home Nurse | 🞎 | 🞎 | 🞎 |
| 12 | Dietician/Nutritionist | 🞎 | 🞎 | 🞎 |
| 13 | Social worker | 🞎 | 🞎 | 🞎 |
| 14 | Physiotherapist | 🞎 | 🞎 | 🞎 |
| 15 | Sexologist | 🞎 | 🞎 | 🞎 |
| 16 | Pain algologist | 🞎 | 🞎 | 🞎 |
| 17 | Psychomotor therapist | 🞎 | 🞎 | 🞎 |
| 18 | Occupational therapist | 🞎 | 🞎 | 🞎 |
| 19 | Entourage (family / friends) | 🞎 | 🞎 | 🞎 |
| 20 | Patient Associations | 🞎 | 🞎 | 🞎 |
| 21 | Other patients met at the hospital | 🞎 | 🞎 | 🞎 |
| 22 | Life assistant | 🞎 | 🞎 | 🞎 |
| 23 | Esthetician / Esthetician | 🞎 | 🞎 | 🞎 |
| 24 | APA (Adapted Physical Activity) Instructor / Educator | 🞎 | 🞎 | 🞎 |
| 25 | Other Patient Caregiver | 🞎 | 🞎 | 🞎 |
| 26 | Other: *please specify*  ……………………………………………………………………… | 🞎 | 🞎 | 🞎 |
| 27 | My loved one is not receiving any support in the face of the repercussions of the disease on their daily life |  | 🞊 |  |
| 28 | I am not receiving any support for the impact of the disease on my daily life |  |  | 🞊 |

**Q37D.** [if the following stakeholders mentioned in Q37] Do the following stakeholders in the management of your loved one's cancer practice...

*Only one possible answer per line*

|  |  | **... At the hospital or clinic** where your loved one is being cared for | ... Outside the hospital or clinic where your loved one is being cared for **(= in the city)** |
| --- | --- | --- | --- |
| 7 | Psychologist, psychiatrist | 🞊 | 🞊 |
| 11 | Dietician/Nutritionist | 🞊 | 🞊 |
| 12 | Social worker | 🞊 | 🞊 |
| 13 | Physiotherapist | 🞊 | 🞊 |
| 14 | Sexologist | 🞊 | 🞊 |
| 15 | Pain algologist | 🞊 | 🞊 |
| 16 | Psychomotor therapist | 🞊 | 🞊 |
| 17 | Occupational therapist | 🞊 | 🞊 |
| 22 | Esthetician / Esthetician | 🞊 | 🞊 |

**Q37E.** [if the following workers mentioned in Q37B] Do the following workers who provide you as a loved one of the sick most support exert...

*Only one possible answer per line*

|  |  | **... At the hospital or clinic** where your loved one is being cared for | ... Outside the hospital or clinic where your loved one is being cared for **(= in the city)** |
| --- | --- | --- | --- |
| 7 | Psychologist, psychiatrist | 🞊 | 🞊 |
| 11 | Dietician/Nutritionist | 🞊 | 🞊 |
| 12 | Social worker | 🞊 | 🞊 |
| 13 | Physiotherapist | 🞊 | 🞊 |
| 14 | Sexologist | 🞊 | 🞊 |
| 15 | Pain algologist | 🞊 | 🞊 |
| 16 | Psychomotor therapist | 🞊 | 🞊 |
| 17 | Occupational therapist | 🞊 | 🞊 |
| 22 | Esthetician / Esthetician | 🞊 | 🞊 |

**Part C. The impact of the disease and its management on your daily life [16-18')**

Now, we would like to know more about how the disease and its management/treatment affect the lives of relatives and patients.

**Q38**. If you had to give a score on a scale of 1 to 10 to **describe the overall impact of your loved one's illness and their care/treatment on your daily life as a caregiver / companion** **over the past month**, what score would you give?

*You have scores from 1 to 10 to answer this question. 1, means that the disease has not limited his daily activities at all; 10, that it has severely limited them. Intermediate grades allow you to modulate your judgment*

| ***1*** 💣 \| ***2*** 💣 \| ***3*** 💣 \| ***4*** 💣 \| ***5*** 💣 \| ***6*** 💣 \| ***7*** 💣 \| ***8*** 💣 \| ***9*** 💣 \| ***10*** 💣 | | |
| --- | --- | --- |
| ***Didn't limit my daily activities at all*** | ***Severely limited my daily activities*** |  |

**Q38B.** Here are a number of disorders that can be expressed by the relatives of sick people, in connection with their role as caregivers.

For each of them, please note:

- On the one hand, if you are currently personally affected
- Then, for each of the disorders that concern you, to what extent it impacts your daily life: you have scores from 1 to 10 to answer this question. 1 means that this disorder does not limit your daily activities at all; 10 that it limits them enormously. Intermediate grades allow you to modulate your judgment
- Finally, has this disorder been treated either through a medical consultation or treatment and if so, are you satisfied with it?

|  | **Are you currently affected?** | **🡆 What impact on your daily life? (score out of 10)** | **🡆 Supported and are you satisfied with it?** |
| --- | --- | --- | --- |
| 1 | **Fatigue**  _1_ ⭘ Yes 🡆  _2_ ⭘ No | _/___/ / 10_ | ⭘ Yes / I am satisfied with it  ⭘ Yes / I'm not satisfied with it  ⭘ No, no support |
| 2 | **Weight problems (weight loss or gain)**  ⭘ Yes 🡆  ⭘ No | _/___/ / 10_ | ⭘ Yes / I am satisfied with it  ⭘ Yes / I'm not satisfied with it  ⭘ No, no support |
| 3 | **Sleep disorders**  ⭘ Yes 🡆  ⭘ No | _/___/ / 10_ | ⭘ Yes / I am satisfied with it  ⭘ Yes / I'm not satisfied with it  ⭘ No, no support |
| 4 | **Low morale**  ⭘ Yes 🡆  ⭘ No | _/___/ / 10_ | ⭘ Yes / I am satisfied with it  ⭘ Yes / I'm not satisfied with it  ⭘ No, no support |
| 5 | **Anxiety/Anguish**  ⭘ Yes 🡆  ⭘ No | _/___/ / 10_ | ⭘ Yes / I am satisfied with it  ⭘ Yes / I'm not satisfied with it  ⭘ No, no support |
| 6 | **Organisational difficulties / burnout related to your role as a caregiver**  ⭘ Yes 🡆  ⭘ No | _/___/ / 10_ | ⭘ Yes / I am satisfied with it  ⭘ Yes / I'm not satisfied with it  ⭘ No, no support |

**[programming, provide a cursor for notes]**

**Here are a number of concrete difficulties/repercussions of the disease on daily life that can be expressed by patients.**

A. Performing everyday gestures

**Q40.** Could you indicate if your loved one is affected by some of these difficulties related to gestures/activities of daily life?

**Be sure to select only the difficulties that you attribute to your loved one's ALK+ lung cancer**

*Several possible answers*

| 1 | It is difficult for them to maintain their home and/or exterior/to perform household chores | 🞎 |
| --- | --- | --- |
| 2 | It is difficult for him to prepare meals | 🞎 |
| 3 | It is difficult for him to wash himself and take care of himself | 🞎 |
| 4 | It is difficult for him to eat | 🞎 |
| 5 | It is difficult for him to stand for too long | 🞎 |
| 6 | It is difficult for him to use public transport | 🞎 |
| 7 | It is difficult for him to maintain physical activity and/or sports | 🞎 |
| 8 | It is difficult for him to concentrate on one activity | 🞎 |
| 9 | Other gestures/activities of daily living that are difficult for your loved one to perform (related to the disease), please specify................................................... | 🞎 |
| 10 | My sick loved one is not affected by any difficulty in carrying out the gestures/activities of daily life that would be related to the disease | 🞊 |

**Q40A.** [if ≠ code 10 in Q40] Has your loved one been able to discuss these difficulties related to gestures/activities of daily living?

*Only one possible answer per column*

|  |  | With a professional in the hospital or clinic who is following your loved one for their cancer | With a professional outside the hospital or clinic in which your loved one is being followed |
| --- | --- | --- | --- |
| **1** | Yes, and it helped | 🞊 | 🞊 |
| **2** | Yes, but it didn't help | 🞊 | 🞊 |
| **3** | No | 🞊 | 🞊 |
| **4** | I don't know | 🞊 | 🞊 |

**Q40B.** [if ≠ code 10 in Q40] To what extent do these difficulties related to the gestures/activities of daily life encountered by your sick loved one affect your own daily life as a loved one?

**Please give a score from 1 to 10.**

*1 = my daily life is not at all affected by these difficulties encountered by my loved one*

*10 = My daily life is very affected by these difficulties encountered by my loved one*

*Intermediate grades allow you to modulate your judgment*

| ***1***  💣 | ***2***  💣 | ***3***  💣 | ***4***  💣 | ***5***  💣 | ***6***  💣 | ***7***  💣 | ***8***  💣 | ***9***  💣 | ***10***  💣 |
| --- | --- | --- | --- | --- | --- | --- | --- | --- | --- |

***My daily life is not affected at all	My daily life is very affected***

**Q40C.** [if ≠10 in Q40] To carry out certain daily tasks, does your loved one benefit from ...

|  | *Only one possible answer per line* | **Yes** | **No** |
| --- | --- | --- | --- |
| 1 | A domestic helper | 🞊 | 🞊 |
| 2 | Meals delivered at home | 🞊 | 🞊 |

**Q40E.** Does your loved one drive a car?

*Only one answer possible*

| 1 | Yes, and he/she has continued to drive since he/she became ill | 🞊 |
| --- | --- | --- |
| 2 | He/she was driving before the illness but has since stopped driving | 🞊 |
| 3 | No, he/she has never driven a car | 🞊 |

**Q40F** [if code 1 or 2 in Q40E] Did his cancer affect his ability to drive long distances?

*Only one answer possible*

| 1 | Yes | 🞊 |
| --- | --- | --- |
| 2 | No | 🞊 |

**Q40G.** [if code 2 in Q40E] For what reason(s) did he/she stop driving?

*Several possible answers*

| 1 | He/she no longer feels capable of doing so | 🞎 |
| --- | --- | --- |
| 2 | Her doctor recommended it | 🞎 |
| 3 | He/she has brain metastases | 🞎 |
| 4 | He/she is taking morphine, or other medication incompatible with driving | 🞎 |
| 5 | Other, please specify............................................. | 🞎 |

**Q40H.** [if code 1 or 2 in Q40E] Has the subject of driving been discussed with the medical team that is treating your loved one for cancer?

*Only one answer possible*

| 1 | Yes | 🞊 |
| --- | --- | --- |
| 2 | No | 🞊 |
| 3 | I don't know | 🞊 |

**Q40I.** [if code 1 or 2 in Q40E] Has your loved one carried out a medical examination of fitness to drive?

*Only one answer possible*

| 1 | Yes | 🞊 |
| --- | --- | --- |
| 2 | No | 🞊 |
| 3 | I don't know | 🞊 |

B. Professional life

**Q41**. What is your loved one's current professional situation?

*Several possible answers*

| 1 | In full-time professional activity | 🞎 |
| --- | --- | --- |
| 2 | In part-time employment | 🞎 |
| 3 | On sick leave | 🞎 |
| 4 | On disability | 🞎 |
| 5 | Looking for a job | 🞎 |
| 6 | School / Student | 🞎 |
| 7 | Retired | 🞎 |
| 8 | Non-active (without profession, ...) | 🞎 |

**Q41B.** [if code 2 or 3 or 4 or 6 or 7 in Q41] Is his current situation [part-time or work stoppage or job search or inactive] related to his ALK+ lung cancer?

| 1 | Yes | 🞊 |
| --- | --- | --- |
| 2 | No | 🞊 |

**Q41C.** What is your own current professional situation?

*Only one answer possible*

| 1 | In full-time professional activity | 🞊 |
| --- | --- | --- |
| 2 | In part-time employment | 🞊 |
| 3 | On sick leave | 🞊 |
| 4 | Looking for a job | 🞊 |
| 5 | School / Student | 🞊 |
| 6 | Retired | 🞊 |
| 7 | Non-active (without profession, ...) | 🞊 |

**Q41D.** [if code 2 or 3 or 4 or 6 or 7 in Q41C] Is your current situation [part-time or work stoppage or job search or inactive] related to your loved one's medical situation?

*Only one answer possible*

| 1 | Yes | 🞊 |
| --- | --- | --- |
| 2 | No | 🞊 |

**Q42.** Could you indicate whether you yourself, as a relative, are affected by some of these professional difficulties?

**Be sure to select only the difficulties related to the disease / your role as a caregiver**

| 1 | I had to adapt my working hours and switch to part-time work to take care of my loved one | 🞎 |
| --- | --- | --- |
| 2 | I lost a job because of my loved one's illness | 🞎 |
| 3 | I have to deal with frequent time off work to take care of my loved one | 🞎 |
| 4 | I had to adjust my working hours due to the illness of my loved one | 🞎 |
| 5 | I had to stop working due to my loved one's illness | 🞎 |
| 6 | I had to change position/profession | 🞎 |
| 7 | I'm having trouble finding a new job | 🞎 |
| 8 | I'm afraid of losing my job | 🞎 |
| 9 | I am having difficulty progressing professionally / I have been refused a promotion | 🞎 |
| 10 | I have relationship difficulties with my employer | 🞎 |
| 11 | I have relationship difficulties with my colleagues | 🞎 |
| 12 | I find it difficult to keep up with my professional obligations as before | 🞎 |
| 13 | If you have any other professional difficulties related to my loved one's illness, please specify........................................................... | 🞎 |
| 14 | I am not affected by any professional difficulty that would be related to my loved one's illness, to my role as a caregiver, even if I have worked or I am working since my loved one has been living with ALK+ lung cancer | 🞊 |
| 15 | I have not been working since my loved one has been living with ALK+ lung cancer | 🞊 |

**Q42A.** [if ≠ code 15 or 16 in Q42] Have you been able to discuss your professional difficulties?

*Only one possible answer per column*

|  |  | With a professional in the hospital or clinic who is following your loved one for their cancer | With a professional outside the hospital or clinic in which your loved one is being followed |
| --- | --- | --- | --- |
| **1** | Yes, and it helped me | 🞊 | 🞊 |
| **2** | Yes, but it didn't help me | 🞊 | 🞊 |
| **3** | No, but I don't want to talk about this type of subject | 🞊 | 🞊 |
| **4** | No, but I would have liked to have been able to address this type of subject | 🞊 | 🞊 |

**Q42C.** [if ≠ code 15 or 16 in Q42] To what extent is your professional life affected by your loved one's illness?

**Please give a score from 1 to 10.**

*1 = My professional life is not affected at all by my loved one's illness*

*10 = my professional life is very affected by my loved one's illness*

*Intermediate grades allow you to modulate your judgment*

| ***1***  💣 | ***2***  💣 | ***3***  💣 | ***4***  💣 | ***5***  💣 | ***6***  💣 | ***7***  💣 | ***8***  💣 | ***9***  💣 | ***10***  💣 |
| --- | --- | --- | --- | --- | --- | --- | --- | --- | --- |

***Not affected at all	Is very affected***

**Q42F.** [if ≠ code 15 or 16 in Q42] Has your employer been a caregiver to adapt your working conditions?

*Only one answer possible*

| **1** | Yes, absolutely | 🞊 |
| --- | --- | --- |
| **2** | Yes partially | 🞊 |
| **3** | No, not at all | 🞊 |
| **4** | My employer doesn't know that I'm accompanying someone who is sick | 🞊 |

**Q42H.** [if code 5 in Q41C] To what extent is your school/student life affected by your loved one's illness?

**Please give a score from 1 to 10.**

*1 = my school/student life is not affected at all by my loved one's illness*

*10 = my school/student life is very affected by my loved one's illness*

*Intermediate grades allow you to modulate your judgment*

| ***1***  💣 | ***2***  💣 | ***3***  💣 | ***4***  💣 | ***5***  💣 | ***6***  💣 | ***7***  💣 | ***8***  💣 | ***9***  💣 | ***10***  💣 |
| --- | --- | --- | --- | --- | --- | --- | --- | --- | --- |

***Not affected at all	Is very affected***

C. Couple life, intimate life

**Q43.** Could you indicate if you are concerned by some of these difficulties in your life as a couple, intimate life?

**Be sure to select only the difficulties related to the disease / your role as a caregiver**

| 1 | I have had difficulty investing in a life as a couple since the illness of my loved one | 🞎 |
| --- | --- | --- |
| 2 | My loved one's illness generates tension in my life as a couple | 🞎 |
| 3 | I feel that my spouse doesn't understand what I'm going through |  |
| 5 | My loved one's illness has strengthened my life as a couple | 🞎 |
| 6 | The disease precipitated my breakup with my spouse | 🞎 |
| 7 | I have been experiencing a drop or loss of libido since my loved one's illness | 🞎 |
| 8 | Other couple or sexual difficulties related to my loved one's illness, please specify......................... | 🞎 |
| 9 | I am not affected by any difficulty in my relationship or sexual life that would be related to the illness of my loved one / my role as a caregiver | 🞊 |
| 10 | I haven't been in a relationship since my loved one is living with ALK+ lung cancer | 🞊 |

**Q43B.** [if ≠ code 9 or 10 in Q43] Have you been able to discuss your marital difficulties?

*Only one possible answer per column*

|  |  | With a professional in the hospital or clinic who is following your loved one for their cancer | With a professional outside the hospital or clinic in which your loved one is being followed |
| --- | --- | --- | --- |
| **1** | Yes, and it helped me | 🞊 | 🞊 |
| **2** | Yes, but it didn't help me | 🞊 | 🞊 |
| **3** | No, but I don't want to talk about this type of subject | 🞊 | 🞊 |
| **4** | No, but I would have liked to have been able to address this type of subject | 🞊 | 🞊 |

**Q43C**. [If sexual difficulties = code 7 in Q43] Has the healthcare team offered you to consult a sexologist or any documentation to overcome the difficulties you are encountering sexually?

*Only one answer possible*

| **1** | Yes | 🞊 |
| --- | --- | --- |
| **2** | No | 🞊 |

**Q43D.** [if ≠ code 9 or 10 in Q43] To what extent is your life as a couple affected by your loved one's illness?

**Please give a score from 1 to 10.**

*1 = my life as a couple is not affected at all by my loved one's illness*

*10 = my life as a couple is very affected by the illness of my loved one*

*Intermediate grades allow you to modulate your judgment*

| ***1***  💣 | ***2***  💣 | ***3***  💣 | ***4***  💣 | ***5***  💣 | ***6***  💣 | ***7***  💣 | ***8***  💣 | ***9***  💣 | ***10***  💣 |
| --- | --- | --- | --- | --- | --- | --- | --- | --- | --- |

***Not affected at all	Is very affected***

D. Family and Friendship Life

**Q44.** Could you indicate if you are affected by some of these difficulties in your family and/or friendship life?

**Be sure to select only the difficulties related to the disease / your role as a caregiver**

| 1 | I don't give my family the time I would like | 🞎 |
| --- | --- | --- |
| 2 | I don't give my friends the time I'd like | 🞎 |
| 3 | My family doesn't understand or doesn't understand that I can't be available as often as I'd like | 🞎 |
| 4 | My friends don't understand or don't understand that I can't be available as often as I'd like | 🞎 |
| 5 | I have difficulty talking about the disease/explaining my loved one's illness to my family and/or friends | 🞎 |
| 6 | It is difficult for me to ask my family and/or friends for help to allow me to be more available for my sick loved one | 🞎 |
| 7 | Since my loved one is sick, I have limited my social/friendly relationships | 🞎 |
| 8 | If you have any other difficulties in family or friendship life related to my loved one's illness, please specify............................................. | 🞎 |
| 9 | I am not affected by any family difficulties that would be related to my loved one's illness/my role as a caregiver | 🞊 |

**Q44B**. [if ≠9 in Q44] Have you been able to discuss your difficulties in your family and/or friendship life?

*Only one possible answer per column*

|  |  | With a professional in the hospital or clinic who is following your loved one for their cancer | With a professional outside the hospital or clinic in which your loved one is being followed |
| --- | --- | --- | --- |
| **1** | Yes, and it helped me | 🞊 | 🞊 |
| **2** | Yes, but it didn't help me | 🞊 | 🞊 |
| **3** | No, but I don't want to talk about this type of subject | 🞊 | 🞊 |
| **4** | No, but I would have liked to have been able to address this type of subject | 🞊 | 🞊 |

**Q44C.** [if ≠9 in Q44] To what extent is your family life affected by your loved one's illness?

**Please give a score from 1 to 10.**

*1 = My family life is not affected at all by my loved one's illness*

*10 = my family life is very affected by my loved one's illness*

*Intermediate grades allow you to modulate your judgment*

| ***1***  💣 | ***2***  💣 | ***3***  💣 | ***4***  💣 | ***5***  💣 | ***6***  💣 | ***7***  💣 | ***8***  💣 | ***9***  💣 | ***10***  💣 |
| --- | --- | --- | --- | --- | --- | --- | --- | --- | --- |

***Not affected at all	Is very affected***

E. Pregnancy/child project

**Q45.** [if spouse of the sick patient] Since your loved one has been living with ALK+ lung cancer, have you had a plan to become pregnant/have a child?

| 1 | Yes, and we were able to have a child | 🞊 |
| --- | --- | --- |
| 2 | Yes, but without success for the moment | 🞊 |
| 3 | Yes, but we gave up on having a child because of the disease | 🞊 |
| 4 | Yes and we have decided to adopt a child | 🞊 |
| 5 | No | 🞊 |

**Q45B**. [if ≠not in Q45] Have you been able to discuss your pregnancy/child project with the medical team that is following your loved one for cancer?

*Only one answer possible*

| **1** | Yes and she was very helpful | 🞊 |
| --- | --- | --- |
| **2** | Yes, but she wasn't particularly helpful | 🞊 |
| **3** | Yes, but she advised us not to embark on this project of having a child | 🞊 |
| **4** | No | 🞊 |

**Q45C.** [if ≠not in Q45] Has the medical team offered a preventive treatment to preserve your fertility or that of your loved one?

| 1 | Yes | 🞊 |
| --- | --- | --- |
| 2 | No | 🞊 |

F. Psychological

**Q46.** Could you indicate if you are affected by some of these psychological difficulties?

**Be sure to select only the difficulties related to the disease / your role as a caregiver**

| 1 | Feeling angry, unfair, unfair, | 🞎 |
| --- | --- | --- |
| 2 | Depressive episodes | 🞎 |
| 3 | Episodes of sadness | 🞎 |
| 4 | Feeling insecure, afraid of tomorrow | 🞎 |
| 5 | Feeling excluded, of being alone | 🞎 |
| 6 | Loss of self-confidence | 🞎 |
| 7 | Mood disorders | 🞎 |
| 8 | Anxiety, Anguish | 🞎 |
| 9 | Dark thoughts | 🞎 |
| 10 | Other psychological difficulties related to the disease, please specify......................................... | 🞎 |
| 11 | I am not affected by any psychological difficulties that would be related to the illness of my loved one / my role as a caregiver | 🞊 |

**Q46B**. [if ≠12 in Q46] Were you able to discuss your psychological difficulties?

*Only one possible answer per column*

|  |  | With a professional in the hospital or clinic who is following your loved one for their cancer | With a professional outside the hospital or clinic in which your loved one is being followed |
| --- | --- | --- | --- |
| **1** | Yes, and it helped me | 🞊 | 🞊 |
| **2** | Yes, but it didn't help me | 🞊 | 🞊 |
| **3** | No, but I don't want to talk about this type of subject | 🞊 | 🞊 |
| **4** | No, but I would have liked to have been able to address this type of subject | 🞊 | 🞊 |

**Q46C.** [if ≠ code 12 in Q46] To what extent do these psychological difficulties related to your loved one's illness affect your daily life?

**Please give a score from 1 to 10.**

*1 = my daily life is not affected at all by these difficulties*

*10 = My daily life is very affected by these difficulties*

*Intermediate grades allow you to modulate your judgment*

| ***1***  💣 | ***2***  💣 | ***3***  💣 | ***4***  💣 | ***5***  💣 | ***6***  💣 | ***7***  💣 | ***8***  💣 | ***9***  💣 | ***10***  💣 |
| --- | --- | --- | --- | --- | --- | --- | --- | --- | --- |

***Not affected at all	Is very affected***

**Q46D.** Would you say today that, faced with your loved one's illness, you feel ...

Please select a maximum of 4 adjectives that best fit you from the list below

| ***1*** | 🞎 |  | Optimistic | | ***6*** | 🞎 |  | Confident | | | ***11*** | 🞎 | Surrounded/listened to |
| --- | --- | --- | --- | --- | --- | --- | --- | --- | --- | --- | --- | --- | --- |
| ***2*** | 🞎 |  | Resigned | | ***7*** | 🞎 |  | Active/Active | | | ***12*** | 🞎 | Angry |
| ***3*** | 🞎 |  | Discouraged | | ***8*** | 🞎 |  | Fragile | | | ***13*** | 🞎 | Lucid |
| ***4*** | 🞎 |  | Combative | | ***9*** | 🞎 |  | Solid | | | ***14*** | 🞎 | Pessimist |
| ***5*** | 🞎 |  | Anxious | | ***10*** | 🞎 |  | Isolated | | | ***15*** | 🞎 | Powerless |
|  |  |  | |  |  |  | | |  |  |  |  |  |

G. Financial angle

**Q47.** Could you indicate whether you are affected by some of these financial difficulties?

**Be sure to select only the difficulties related to the disease / your role as a caregiver**

*Several possible answers*

| 1 | I have experienced a drop in income in the context of my professional activity (loss of a job, part-time work, change of profession) | 🞎 |
| --- | --- | --- |
| 2 | [if spouse] My sick relative has experienced a drop in income in the context of his/her professional activity (loss of a job, transition to part-time work, change of profession, disability, work stoppage,...) | 🞎 |
| 3 | [if spouse] I had difficulty obtaining a loan due to the illness of my loved one | 🞎 |
| 4 | [if spouse] I was refused a loan because of my loved one's illness | 🞎 |
| 5 | I lack/have long lacked information on possible social assistance | 🞎 |
| 6 | If you have any other financial difficulties related to my loved one's illness, please specify........................................................... | 🞎 |
| 7 | I am not affected by any financial difficulties related to my loved one's illness | 🞊 |

**Q47B**. [if ≠9 in Q47] Were you able to discuss your financial difficulties?

*Only one possible answer per column*

|  |  | With a professional in the hospital or clinic who is following your loved one for their cancer | With a professional outside the hospital or clinic in which your loved one is being followed |
| --- | --- | --- | --- |
| **1** | Yes, and it helped me | 🞊 | 🞊 |
| **2** | Yes, but it didn't help me | 🞊 | 🞊 |
| **3** | No, but I don't want to talk about this type of subject | 🞊 | 🞊 |
| **4** | No, but I would have liked to have been able to address this type of subject | 🞊 | 🞊 |

**Q47C.** [if ≠ code 9 in Q47] To what extent do these financial difficulties related to your loved one's illness affect your daily life?

**Please give a score from 1 to 10.**

*1 = My daily life/quality of life is not affected at all by these difficulties*

*10 = My daily life/quality of life is very affected by these difficulties*

*Intermediate grades allow you to modulate your judgment*

| ***1***  💣 | ***2***  💣 | ***3***  💣 | ***4***  💣 | ***5***  💣 | ***6***  💣 | ***7***  💣 | ***8***  💣 | ***9***  💣 | ***10***  💣 |
| --- | --- | --- | --- | --- | --- | --- | --- | --- | --- |

***Not affected at all	Is very affected***

H. Life projects

**Q48.** Could you indicate whether you are affected by some of these difficulties in the implementation of your projects?

**Be sure to select only the difficulties related to the disease / your role as a caregiver**

*Several possible answers*

| 1 | I have difficulty projecting myself into the future | 🞎 |
| --- | --- | --- |
| 2 | I have difficulty setting up projects, planning | 🞎 |
| 3 | I don't feel able to carry out my projects | 🞎 |
| 4 | I had to give up some sports activities | 🞎 |
| 5 | I had to give up certain cultural and/or artistic activities | 🞎 |
| 6 | I had to give up organizing trips, vacation plans | 🞎 |
| 7 | Other difficulties related to my life projects related to my loved one's illness | 🞎 |
| 8 | I am not concerned by any difficulty in making plans for someone who would be related to my loved one's illness | 🞊 |

**Q48B**. [if ≠8 in Q48] Were you able to discuss your difficulties in carrying out your projects?

*Only one possible answer per column*

|  |  | With a professional in the hospital or clinic who is following your loved one for their cancer | With a professional outside the hospital or clinic in which your loved one is being followed |
| --- | --- | --- | --- |
| **1** | Yes, and it helped me | 🞊 | 🞊 |
| **2** | Yes, but it didn't help me | 🞊 | 🞊 |
| **3** | No, but I don't want to talk about this type of subject | 🞊 | 🞊 |
| **4** | No, but I would have liked to have been able to address this type of subject | 🞊 | 🞊 |

**Q48C.** [if ≠ code 8 in Q48] To what extent do these difficulties in carrying out your projects affect your daily life?

**Please give a score from 1 to 10.**

*1 = My daily life/quality of life is not affected at all by these difficulties*

*10 = My daily life/quality of life is very affected by these difficulties*

*Intermediate grades allow you to modulate your judgment*

| ***1***  💣 | ***2***  💣 | ***3***  💣 | ***4***  💣 | ***5***  💣 | ***6***  💣 | ***7***  💣 | ***8***  💣 | ***9***  💣 | ***10***  💣 |
| --- | --- | --- | --- | --- | --- | --- | --- | --- | --- |

***Not affected at all	Is heavily affected***

**Q54.** In short, among all these difficulties caused by your loved one's illness/your role as a caregiver, please select the 3 that affect your daily life the most?

Please rank in hierarchical order and rate 1 for the difficulty that affects your daily life the most, then 2 for the difficulty you rank in 2nd position, then 3 for the third

[prog instruction: if less than 3 groups of difficulties mentioned from Q40 to Q48] please select the one that affects your daily life the most?

| 1 | [if ≠ code 15 or 16 in Q42] Difficulties in working life | /____/ |
| --- | --- | --- |
| 2 | [if code 5 in Q41C] Difficulties in school/student life | /____/ |
| 3 | [if ≠ code 9 or 10 in Q43] Difficulties in couples' life, intimate life | /____/ |
| 4 | [if ≠9 in Q44] Difficulties in family life, friendships | /____/ |
| 5 | [if ≠5 in Q45] Difficulties related to a pregnancy/child project | /____/ |
| 6 | [if ≠ code 12 in Q46] Psychological, moral difficulties | /____/ |
| 7 | [if ≠9 in Q47] Financial Hardship | /____/ |
| 8 | [if ≠ code 8 in Q48] Difficulties in carrying out your life projects | /____/ |

**Support needs**

**Q50.** Here are various actions/initiatives that could be put in place to support the relatives of patients with ALK+ cancer. How important do you think they are to put in place?

**Please give a score from 1 to 10.**

*1 = action not at all important to put in place according to you*

*10 = very important action to put in place according to you*

*Intermediate grades allow you to modulate your judgment*

| 1 | Have additional information about the disease and its evolution | /____/ |
| --- | --- | --- |
| 2 | To have tools and supports to better train myself as a caregiver | /____/ |
| 3 | Learn to better dialogue/communicate with a sick person | /____/ |
| 4 | Learning to reconcile my professional life with my loved one's illness | /____/ |
| 5 | Have information about my rights as a caregiver | /____/ |
| 6 | Have time for respite through the offer of well-being activities (yoga, meditation, sophrology, shiatsu, adapted physical activity, etc.) | /____/ |
| 7 | Offering support groups for the relatives of patients | /____/ |
| 8 | Offer me psychological support to better cope with my role as a caregiver and/or my loved one's illness | /____/ |
| 9 | Set up a "Quality of life" consultation in the day hospital for patients and their carers which would be dedicated to the non-medical difficulties encountered (social, family, professional, etc.) | /____/ |

**Signage [1 to 2']**

Finally, thank you for answering these few socio-demographic questions

**S11.** May I ask you your age please?

*Please indicate your age below*

/________/Years

**S12.** You are...

*Only one answer possible*

| 1 | A man | 🞊 |
| --- | --- | --- |
| 2 | A woman | 🞊 |
| 3 | I don't want to answer | 🞊 |

**S13.** Do you have children?

*Only one answer possible*

| 1 | Yes | 🞊 |
| --- | --- | --- |
| 2 | No | 🞊 |

**S14.** [if yes in S13] Your child has / your children have ...

*Several possible answers*

| 1 | Under 10 years old | 🞎 | to Number /___/ |
| --- | --- | --- | --- |
| 2 | Between 10 and 17 years old | 🞎 | Number/___/ |
| 3 | 18 years and older | 🞎 | Number/___/ |

**S15.** In your home, you live...

*Only one answer possible*

| 1 | Alone, with children | 🞊 |
| --- | --- | --- |
| 2 | Alone without children | 🞊 |
| 3 | As a couple, with children | 🞊 |
| 4 | As a couple, without children | 🞊 |
| 5 | With my parents or one of my parents/siblings | 🞊 |

**S16.** What is (or was) your profession...

*If you are not currently working or are no longer working, but have already worked, please enter your former profession here / only one possible answer*

| 1 | Farmer, operator (self-employed) | 🞊 |
| --- | --- | --- |
| 2 | Craftsman, shopkeeper | 🞊 |
| 3 | Business Manager | 🞊 |
| 4 | Executive and other higher intellectual profession | 🞊 |
| 5 | Liberal professions (except paramedics) | 🞊 |
| 6 | Professor, scientific profession | 🞊 |
| 7 | Foreman, supervisor, paramedical profession, technician | 🞊 |
| 8 | Primary school teacher or equivalent | 🞊 |
| 9 | Employee | 🞊 |
| 10 | Service staff | 🞊 |
| 11 | Worker | 🞊 |
| 12 | Student | 🞊 |
| 13 | No profession | 🞊 |

**S17.** You live ...

*Only one answer possible*

| 1 | In a large metropolis: more than 100,000 inhabitants | 🞊 |
| --- | --- | --- |
| 2 | In a large city: 50,000 to 99,999 inhabitants | 🞊 |
| 3 | In a medium-sized city: from 10,000 to 49,999 inhabitants | 🞊 |
| 4 | In a small town: 2000 to 9,999 inhabitants | 🞊 |
| 5 | In rural areas: less than 2000 inhabitants | 🞊 |

**S18.** On average, how long does it take you to get to the hospital where your loved one is being treated for their ALK+ lung cancer?

*Only one answer possible*

| 1 | Less than 30 minutes | 🞊 |
| --- | --- | --- |
| 2 | Between 30 minutes and less than 1 hour | 🞊 |
| 3 | Approximately 1 hour | 🞊 |
| 4 | Between 1 and 2 hours | 🞊 |
| 5 | More than 2 hours | 🞊 |

**S18B.** What means of transport do you use most often when you travel with your loved one to accompany them to the hospital?

*Only one answer possible*

| 1 | Public transport | 🞊 |
| --- | --- | --- |
| 2 | My car | 🞊 |
| 3 | My loved one's car | 🞊 |
| 4 | Taxi or VSL | 🞊 |
| 5 | Others, please specify /_____________________/ | 🞊 |
| 6 | I don't take care of my sick relative's travel, he/she manages his/her travels alone | 🞊 |

**S18C.** You live...

*Only one answer possible*

| 1 | In the same home as your sick loved one | 🞊 |  |
| --- | --- | --- | --- |
| 2 | Away from home | 🞊 |  |

**S18D**. [if code 2 in S18C] On average, how long does it take you to get to your loved one's home?

*Only one answer possible*

| 1 | Less than 30 minutes | 🞊 |
| --- | --- | --- |
| 2 | Between 30 minutes and less than 1 hour | 🞊 |
| 3 | Approximately 1 hour | 🞊 |
| 4 | Between 1 and 2 hours | 🞊 |
| 5 | More than 2 hours | 🞊 |

**S19.** What is your level of education (highest degree you have obtained)?

*Only one answer possible*

| 1 | Up to primary education | 🞊 |
| --- | --- | --- |
| 2 | Until secondary education without the baccalaureate | 🞊 |
| 3 | Baccalaureate without post-baccalaureate studies | 🞊 |
| 4 | Baccalaureate + 2 years of post-baccalaureate studies | 🞊 |
| 5 | Baccalaureate + 3 years of post-baccalaureate studies | 🞊 |
| 6 | Baccalaureate + 4 years of post-baccalaureate studies and more | 🞊 |

**S20.** Of the following brackets, what is your net monthly household income (on average)?

*Please count all the resources of your household: income, family allowances and other financial resources of all members of the household / Please check only one answer*

*Only one answer possible*

| 1 | Under €499 | 🞊 |
| --- | --- | --- |
| 2 | From 500 to 1,499 € | 🞊 |
| 3 | From €1,500 to €2,499 | 🞊 |
| 4 | From €2,500 to €3,499 | 🞊 |
| 5 | From €3,500 to €4,499 | 🞊 |
| 6 | From €4,500 to €5,499 | 🞊 |
| 7 | From €5,500 to €6,499 | 🞊 |
| 8 | From €6,500 to €7,499 | 🞊 |
| 9 | 7500 € and more | 🞊 |
| 10 | Don't want to answer | 🞊 |

**S21.** Which of the following aids do you or your sick loved one receive in the context of their ALK+ lung cancer?

*Several possible answers except last line*

| 1 | Disability allowance | □ |
| --- | --- | --- |
| 2 | RQTH (recognition of the status of disabled worker) | □ |
| 3 | ALD (100% coverage - Long-term condition) | □ |
| 4 | Home Helper | □ |
| 5 | Life assistant | □ |
| 4 | My loved one does not receive any of these benefits | 🞊 |

**S22**. Do you know of one or more patient associations involved in lung cancer?

*Only one answer possible*

| **1** | Yes | 🞊 |
| --- | --- | --- |
| **2** | No | 🞊 |

**S22B**. [if yes in S22] What patient associations involved in lung cancer do you know?

**S22C.** [If yes in S22] How did you find out about these patient associations involved in lung cancer?

*Several possible answers*

| **1** | Via my loved one's oncologist | □ |
| --- | --- | --- |
| **2** | Via another member of the medical team | □ |
| **3** | Via the attending physician | □ |
| **4** | Through my own personal research | □ |
| **5** | Via my relatives | □ |
| **6** | Through other patients | □ |

**S23.** [If yes in S22] What is your relationship with patient associations involved in lung cancer?

*Several possible answers*

|  |  | **Yes** | **No** |
| --- | --- | --- | --- |
| **1** | You are a member of one of these patient associations | 🞊 | 🞊 |
| **2** | You have participated in some discussion groups organized by a patient association | 🞊 | 🞊 |
| **3** | You have visited the website of one or more patient associations | 🞊 | 🞊 |
| **4** | You have called one or more patient organizations for information | 🞊 | 🞊 |
| **5** | You have participated in workshops or events organized by a patient association | 🞊 | 🞊 |

**This questionnaire is now closed, thank you for your participation!**
