# Supplementary material for: Living With ALK Fusion-Positive NSCLC: A French Nationwide Survey of the Psychosocial Impact Among Patients, Caregivers, and Health Care Professionals
Source: JTO Clin Res Rep. 2026 Jun 30;7(10):101038. doi: 10.1016/j.jtocrr.2026.101038 (PMC13626941; doi:10.1016/j.jtocrr.2026.101038)
Supplement: Supplementary file 4 [file mmc4.docx]

**Appendix C**

**Towards a chronicization of ALK+ NSCLC: patients' experiences and needs in the face of this disease**

**PRESENTATION OF THE SURVEY AND REGULATORY ASPECTS RELATED TO YOUR PARTICIPATION [do not display]**

Hello Doctor and welcome to the website of AplusA, a market research company specializing in the field of health.

This information explains the objectives of this survey, who initiated it and how your personal data is being participated in and processed.

**Why this survey?**

The objective of this survey ("Survey") is to report on the impact of the disease and its management on the daily lives of patients, to precisely assess the societal issues (family, professional, social, mental health, etc.).

In more detail, this Survey should make it possible to:

- To better understand the patient's journeys and their experiences of the disease at the different stages of their journey.
- To study the concrete impact of the disease on the daily lives of patients and their caregivers, to bring out the difficulties encountered by patients in their daily lives and in their future projects.
- To analyse the support needs and expectations of patients and their relatives to improve their daily lives with the disease.

This survey is **conducted among healthcare professionals and patients and/or caregivers**.

The results of the survey will be published and will make it possible to make visible the impact of ALK+ bronchial cancer on the daily lives of patients and their close relatives.

**Who is behind the establishment of this Survey?**

This Survey was initiated by a pharmaceutical laboratory ("Sponsor") and is led by a scientific committee bringing together a group of medical experts in this disease and patient associations.

The **various members of the scientific committee** are:

- Professor Jacques Cadranel (Pulmonologist, APHP, Tenon, Paris)
- Doctor Romain Corre (Pulmonologist, Cornouaille Quimper Hospital, Concarneau)
- Doctor Bertrand Mennecier (Pulmonologist, CHRU Nancy)
- Doctor Alice Mogenet (Pulmonologist, APMH, Marseille)
- Peggy Mouton (Advanced Practice Nurse, CHRU Lille)
- Network Patient Association
- Association ALK+ ROS1 France
- Association "PATIENTS EN RESEAU - Mon Réseau Cancer du Poumon", Paris, France

The company *AplusA,* which specialises in health surveys, was mandated to carry out this survey.

**How does the Inquiry work?**

The Inquiry takes place in France.

This is a **survey called "doctor/patient mirror".**

In practice, it consists of you:

**1/ Get 2 to 4 of your patients** with ALK+ NSCLC to participate **by giving them the questionnaire (online version accessible via a QR code).** Your patients will complete this questionnaire on their own. The inclusion of your patients will be done as you go through your next consultations without any patient selection criteria on your part.

**2/ Fill in an online questionnaire (an access link will be sent to you)**

In order to take into account the time spent, compensation will be given to you. This compensation will vary according to the number of patients you include (see explanations on the following screens). Compensation is also provided to patients who participate in this survey.

By agreeing to participate in this Survey, you confirm that no legal obligation, in particular the obligation set out in Article 432-13 of the Penal, Regulatory or Ethical Code, prevents you from participating in this Survey and from being compensated for this participation.

The Survey will be based solely on the analysis of questionnaires.

**About your privacy**

*AplusA* is a research company specialising in the field of health. As a member of ESOMAR and EphMRA, *AplusA* respects the International Code of Practice for Opinion Research. APLUSA also complies with ASOCS.

In order not to bias the quality of the responses collected during this Survey, the name of the Sponsor will remain confidential. You may be informed of the identity of the Sponsor if you request it from AplusA at the end of the Survey. If you wish to have their identity revealed beforehand during the Survey, you may make a request to AplusA at any time, but this will terminate your participation in this Survey.

We would like to assure you that the information you provide will be treated confidentially. In order to preserve your anonymity, the Sponsor will not have access to your directly identifying personal data (such as your surname, first name), except in the context of pharmacovigilance and quality control feedback, as defined below. You agree to maintain the confidentiality of any new information or potential new products, as appropriate. You are free to terminate this survey at any time.

**About the transparency of ties of interest and the framework of benefits**

Your participation in this survey will be the subject of a "Market Research" agreement between you and AplusA, the main information of which will be processed in accordance with the applicable regulations on the protection of personal data and published in accordance with the regulations on the transparency of links of interest (Article L.1453-1 of the Public Health Code).

AplusA will transmit the information required for publication on the single public website https://www.transparence.sante.gouv.fr/pages/accueil/. The information, in particular your personal data, published is as follows: surname, first name, title, address, RPPS number, order number of the Health Professional, subject of the agreement (market study), date of signature of the agreement and possibly the nature of the benefit and its amount including VAT.

Your personal data processed in this context will only be kept for as long as necessary for the purposes for which they were collected and for a maximum period of ten (10) years.

You have the right to access and rectify the data published by contacting AplusA: [dpo@aplusaresearch.com](mailto:dpo@aplusaresearch.com). As this is a legal obligation, you cannot object to it.

This agreement will also be the subject of a prior declaration to your Council of the Order in accordance with the regulations on the supervision of benefits (Article L.1453-3 et seq. of the Public Health Code).

In return for your collaboration, you will receive the following compensation(s):

- **a compensation of €50 including taxes per patient included in the survey** (a minimum of 2 patients and a maximum of 4 patients can be included for each doctor) = > receipt of the questionnaire completed by the patient.

The patient will receive compensation for his or her participation, in the amount of €25. The refilling time is estimated at 35 minutes.

- **Compensation of €75 euros including taxes for the questionnaire that you will complete online**. The refilling time is estimated at about 35 minutes.

To comply with applicable regulations, by agreeing to participate in this Survey, you consider that the compensation provided for your participation corresponds to normal compensation for your participation. It is your responsibility to make any necessary declarations to the Departmental Council of the Order or the relevant body to which you belong, as well as to the tax services.

If you are a hospital doctor or university hospital, according to [Decree No. 2007-658 of 2 May 2007 relating to the combination of activities of civil servants, non-tenured civil servants under public law and workers in State industrial establishments - Légifrance (legifrance.gouv.fr),](https://www.legifrance.gouv.fr/jorf/id/JORFTEXT000000649834) you confirm that you have obtained the necessary authorisations and you undertake to comply with all of the said provisions.

**Data processing in the context of pharmacovigilance and quality assurance**

During this Survey, you may be required to disclose information, including personal data, relating to the safety or quality of one or more of the Sponsor's therapeutic products or medical devices. In some cases, there may be certain regulatory and legal obligations to report certain types of safety or quality information to regulatory agencies (e.g., U.S. Food and Drug Administration, European Medicines Agency). The processing of this information in the context of pharmacovigilance and quality assurance is also in the public interest in the field of public health. It should therefore be noted that we may be required to report to the Sponsor and/or the Sponsor's authorized delegates, business partners or collaborators the safety and quality information you have mentioned.

Sponsor and/or Sponsor's authorized designee may need to contact you for additional information necessary to comply with Sponsor's regulatory and legal reporting obligations.

**About the processing of your personal data**

As part of this Survey, some of your personal data may be collected and subject to automated processing by AplusA. The personal data processed for the purpose of carrying out this Survey and collected directly from you may include your surname, first name, specialty, email address, postal address, department, as well as data relating to your professional life.

This processing is based on the Client's legitimate interest in carrying out this Survey.

For the purposes set out in this policy, your personal data may be shared with the other subsidiaries of the Client's group, the service providers with whom the Sponsor collaborates, as well as any their subcontractors. In this context, your personal data may be transferred to other countries that do not offer the same level of protection as the data protection laws applicable in your own country. Safeguards will be put in place where necessary to adequately protect your personal data, including standard contractual clauses.

You may request access, rectification and deletion of your personal data processed in the context of the Survey, and request the limitation of their processing, as well as object to it (if applicable) and request their portability (if applicable).

For any request concerning the processing of your personal data by AplusA or if you wish to exercise your rights, you can contact AplusA's Data Protection Officer by e-mail: [dpo@aplusaresearch.com](mailto:dpo@aplusaresearch.com).

If you have any complaints about the processing of your personal data, you can contact the competent data protection authority. In France, it is the CNIL: Commission Nationale de l'Informatique et des Libertés - www.cnil.fr. Postal address: 3 Place de Fontenoy - TSA 80715 - 75334 PARIS CEDEX 07. Tel: 01 53 73 22 22 / Fax: 01 53 73 22 00.

To consult AplusA's privacy policy: [Privacy Policy for Surveys - APLUSA (aplusaresearch.com).](https://www.aplusaresearch.com/fr/politique-de-confidentialite-pour-les-enquetes/)

AplusA may transfer your data to the Sponsor or to service providers based outside the European Economic Area (EEA) under the strict conditions permitted by law, including protective measures that will be put in place where necessary to adequately protect your personal data processed in the context of the Survey, in particular through the implementation of standard contractual clauses.

Your data will be stored on AplusA's secure servers in pseudonymized form for a period of three (3) years after the end of the Survey, but this period may not exceed that necessary for the purposes for which they were collected, and this is subject to applicable local laws and regulations.

**[R4 – Final Consent]**

**R4.** I confirm that all information relating to this Survey has been duly explained to me, I confirm my agreement with the terms and conditions and I confirm my participation in this Survey.

Please note that your agreements will be valid for recruitment and interviews for the duration of this Survey.

| **1** | Yes | ○ |
| --- | --- | --- |
| **2** | No | ○ |

| TYPE | CODED UNIQUE – mandatory |
| --- | --- |
| ROUTINE | All Respondents |
| LOGIC | If CODE 2 = thanks and close |

**RECRUITMENT SCREENER [4']** [do not display]

Now, I am going to ask you a few questions because we want to diversify the profile of the doctors included in the survey.

**S1. Could you please confirm your specialty?**

| **1** | Oncologist | ○ |
| --- | --- | --- |
| **2** | Pulmonologist | ○ |
| **3** | Other | ○ |

| TYPE | CODED UNIQUE – mandatory |
| --- | --- |
| ROUTINE | All Respondents |
| LOGIC | If code 2 selected thanks and close |

**S2. You mainly work as a ...**

| **1** | In CH(R)U/ AP / HCL | ○ |
| --- | --- | --- |
| **2** | In CH / CHG / ESPIC | ○ |
| **3** | In PBC | ○ |
| **4** | In clinic, private hospital | ○ |

| TYPE | CODED UNIQUE – mandatory |
| --- | --- |
| ROUTINE | All Respondents |
| LOGIC | No quotas |

**S3. In which department is your establishment located?**

/___/ Please note the first two digits of your department

| TYPE | NUMERIC MIN 01 MAX 99 – mandatory |
| --- | --- |
| ROUTINE | All Respondents |
| LOGIC |  |

**S4. How many years have you been practicing?**

/___/ years	○ less than one year

| TYPE | NUMERIC MIN 1 MAX 50 – mandatory |
| --- | --- |
| ROUTINE | All Respondents |
| LOGIC | If less than a year thanks and close |

**S5. How many patients do you estimate to have ALK+ NSCLC in your active file, whether it is a localized, locally advanced or metastatic form?**

/___/ ALK+ NSCLC patients in my active queue

| TYPE | NUMERIC MIN 0 MAX 999 – mandatory |
| --- | --- |
| ROUTINE | All Respondents |
| LOGIC | If < 2 thanks and close |

**S6. How are these [shown in S5] patients divided into the following different forms?**

| **1** | Present in localized **ALK+ NSCLC** | /___/ patients |
| --- | --- | --- |
| **2** | Have locally advanced **ALK+ NSCLC** | /___/ patients |
| **3** | Have metastatic **ALK+ NSCLC** | /___/ patients |
|  | Total = | N in S5 |

| TYPE | NUMERIC MIN 0 MAX 999 – mandatory |
| --- | --- |
| ROUTINE | All Respondents |
| LOGIC | If 2+3 < 2 thanks and close |

**S7. How many of your patients with locally advanced or metastatic ALK+ NSCLC [shown=2+3 in S6]?**

/___/ patients with locally advanced or metastatic ALK+ NSCLC treated with targeted therapy

| TYPE | NUMERIC MIN 0 MAX 999 – mandatory |
| --- | --- |
| ROUTINE | All Respondents |
| LOGIC | If < 2 thanks and close |

**S8. And how are these [shown in S7] patients on targeted therapy divided according to the following lines of treatment?**

| **1** | Online 1 treatment | /___/ patients |
| --- | --- | --- |
| **2** | Online 2+ processing | /___/ patients |
|  | Total = | N in S7 |

| TYPE | NUMERIC MIN 0 MAX 999 – mandatory |
| --- | --- |
| ROUTINE | All Respondents |
| LOGIC | If line 1 = 0 thanks and close  If line 2 and + = 0 thanks and close |

**S9. How many of your [shown in S8 – line 1] patients on targeted therapy in line 1 are treated with targeted therapy**

| **1** | For less than a year | /___/ patients |
| --- | --- | --- |
| **2** | For a year and more | /___/ patients |
|  | Total = | N in S8 – line 1 |

| TYPE | NUMERIC MIN 0 MAX 999 – mandatory |
| --- | --- |
| ROUTINE | All Respondents |
| LOGIC | If line 2 = 0 thanks and close |

**S10. Do you agree to give a questionnaire to some of your patients (reminder: between 2 and 4 patients)?**

Here is **the profile of patients to be included** in this survey:

**Patients must have locally advanced or metastatic ALK+ NSCLC and be on targeted therapy at the time of the investigation.**

**1 or 2 patients in L1 for at least 12 months**

**1 or 2 patients in L2 or more for at least 2 years**

| **1** | Yes, I agree to give a questionnaire to some of my patients | ○ |
| --- | --- | --- |
| **2** | No, I don't accept | ○ |

| TYPE | CODED UNIQUE – mandatory |
| --- | --- |
| ROUTINE | All Respondents |
| LOGIC |  |

**S11. [if code 1 in S10] How many patients in each of these profiles do you plan to include in the survey?**

| **1** | Patient **with locally advanced or metastatic ALK+ NSCLC on L1-targeted therapy for at least 12 months** | ○ 1 patient  ○ 2 patients |
| --- | --- | --- |
| **2** | Patient **with locally advanced or metastatic ALK+ NSCLC on L2-targeted therapy or higher for at least 2 years** | ○ 1 patient  ○ 2 patients |

| TYPE | CODED UNIQUE – mandatory |
| --- | --- |
| ROUTINE | All Respondents |
| LOGIC |  |

**S13. [if code 1 or 2 in S10] Would you like to participate in the survey yourself, which, as a reminder, consists of filling out a 35-minute online questionnaire?**

| **1** | Yes | ○ |
| --- | --- | --- |
| **2** | No | ○ |

| TYPE | CODED UNIQUE – mandatory |
| --- | --- |
| ROUTINE | All Respondents |
| LOGIC | If code 2 in S8 and 2 in S11 Thanks and close |

Thank you very much for your participation in this survey.

[if code 1 in S10 and code 1 in S13] We will send you the file for your patients by post and at the same time send you a link to your own questionnaire.

Please fill in your contact details below

Doctor's contact information

Name:/__________________________________________________/

Forename:/__________________________________________________/

Address:/__________________________________________________/

Such:/__________________________________________________/

Email:/__________________________________________________/

| TYPE | BOX OF TEXT – mandatory |
| --- | --- |
| ROUTINE | All Respondents |
| LOGIC | For Tel has to be a phone number  For email has to be an email address |

[if code 2 in S8 and code 1 in S11] You can now fill in your questionnaire.

**DOCTORS' QUESTIONNAIRE**

**PART A: Profile of your patients with ALK+ NSCLC [3']**

Please note that this survey focuses on patients with locally advanced or metastatic ALK+ NSCLC on targeted therapy, whether they are L1 or in a more advanced line.

Let's start with the socio-demographic profile of your active patient line...

**Q1.** ... How many in % are:

Attention: we are talking about your patients with locally advanced or metastatic ALK+ NSCLC on targeted therapy...

| **1** | Male | /___/ |
| --- | --- | --- |
| **2** | Female | /___/ |
|  | Total = | =100% |

**Q2.** ... How do they break down into the next **age groups**?

Attention: we are talking about your patients with locally advanced or metastatic ALK+ NSCLC on targeted therapy...

| **1** | Under 30 years of age | /___/ |
| --- | --- | --- |
| **2** | 30 to 50 years | /___/ |
| **3** | Over 50 years | /___/ |
|  | Total = | =100% |

**Q3.** What proportion of your patients ...

Attention: we are talking about your patients with locally advanced or metastatic ALK+ NSCLC on targeted therapy...

|  |  | The majority of my patients | A minority of my patients | I don't know |
| --- | --- | --- | --- | --- |
| **1** | **live alone** in their own home | 🞊 | 🞊 | 🞊 |
| **2** | **live with one or more children** in their household | 🞊 | 🞊 | 🞊 |
| **3** | **have a family caregiver** who regularly accompanies them through the disease**?** | 🞊 | 🞊 | 🞊 |
| **4** | **have benefited or currently benefit from a professional caregiver** (home help service)? | 🞊 | 🞊 | 🞊 |
| **5** | **have received or are currently receiving a disability pension** at some point in their care pathway | 🞊 | 🞊 | 🞊 |

**PART B: Accessible care within the institution [2']**

Let's now talk about the different services and care devices available to patients with locally advanced ALK+ NSCLC or metastatic on targeted therapy within your establishment.

**Q7.** For your oncology patients, do you have access to the following services within the facility in which you primarily practice?

|  |  | **Yes** | **No** |
| --- | --- | --- | --- |
| 1 | Therapeutic education workshops |  | 🞊 |
| 2 | LPNs (Advanced Practice Nurses) | 🞊 | 🞊 |
| 3 | IDEC (Coordination Nurses) | 🞊 | 🞊 |
| 4 | Meeting and information space dedicated to cancer patients | 🞊 | 🞊 |
| 5 | Medication reconciliation with a pharmacist | 🞊 | 🞊 |
| 6 | Educational tools to support shared medical decision-making to facilitate exchanges between healthcare professionals and patients (videos, brochures, etc.) | 🞊 | 🞊 |
| 7 | Telephone support for patients undergoing systemic treatment (by a nurse or medical secretary) | 🞊 | 🞊 |
| 8 | Remote monitoring platform | 🞊 | 🞊 |

**Q8.** [filter YES in Q7] Do you use each of these services for your patients with locally advanced or metastatic ALK+ NSCLC on targeted therapy?

|  |  | **Yes, systematically** | **Yes, for the majority** | **Yes, for a minority** | **No** |
| --- | --- | --- | --- | --- | --- |
| 1 | Therapeutic education workshops | 🞊 | 🞊 | 🞊 | 🞊 |
| 2 | LPNs (Advanced Practice Nurses) | 🞊 | 🞊 | 🞊 | 🞊 |
| 3 | IDEC (Coordination Nurses) | 🞊 | 🞊 | 🞊 | 🞊 |
| 4 | Meeting and information space dedicated to cancer patients | 🞊 | 🞊 | 🞊 | 🞊 |
| 5 | Medication reconciliation with a pharmacist | 🞊 | 🞊 | 🞊 | 🞊 |
| 6 | Shared medical decision support tools | 🞊 | 🞊 | 🞊 | 🞊 |
| 7 | Telephone support for patients undergoing systemic treatment (by a nurse or medical secretary) | 🞊 | 🞊 | 🞊 | 🞊 |
| 8 | Remote monitoring platform | 🞊 | 🞊 | 🞊 | 🞊 |

**Q9.** Among the following list of supportive oncology care (SOS), which are available to your oncology patients (in the broad sense) within the establishment in which you mainly practice?

| 1 | Psychologist | 🞎 |
| --- | --- | --- |
| 2 | Dietician, nutritionist | 🞎 |
| 3 | Social worker | 🞎 |
| 4 | Physiotherapist | 🞎 |
| 5 | Sexologist | 🞎 |
| 6 | Algologist (pain doctor) | 🞎 |
| 7 | Psychomotor therapist | 🞎 |
| 8 | Occupational therapist | 🞎 |
| 9 | Esthetician | 🞎 |
| 10 | Adapted Physical Activity (APA) | 🞎 |
| 11 | My school does not offer any of these SOS | 🞊 |

**Q10.** [SOS filter cited in Q9] Do you use each of these SOS for your patients with locally advanced or metastatic ALK+ NSCLC on targeted therapy?

|  |  | **Yes, systematically** | **Yes, for the majority** | **Yes, for a minority** | **No** |
| --- | --- | --- | --- | --- | --- |
| 1 | Psychologist | 🞊 | 🞊 | 🞊 | 🞊 |
| 2 | Dietician, nutritionist | 🞊 | 🞊 | 🞊 | 🞊 |
| 3 | Social worker | 🞊 | 🞊 | 🞊 | 🞊 |
| 4 | Physiotherapist | 🞊 | 🞊 | 🞊 | 🞊 |
| 5 | Sexologist | 🞊 | 🞊 | 🞊 | 🞊 |
| 6 | Algologist (pain doctor) | 🞊 | 🞊 | 🞊 | 🞊 |
| 7 | Psychomotor therapist | 🞊 | 🞊 | 🞊 | 🞊 |
| 8 | Occupational therapist | 🞊 | 🞊 | 🞊 | 🞊 |
| 9 | Esthetician | 🞊 | 🞊 | 🞊 | 🞊 |
| 10 | Adapted Physical Activity (APA) | 🞊 | 🞊 | 🞊 | 🞊 |

**PART C: The announcement of the initial diagnosis of ALK+ NSCLC and relapse [4']**

Let's now look at the time of diagnosis in your patients currently suffering from locally advanced ALK+ NSCLC or metastatic on targeted therapy.

**Q11.** Concerning your patients currently suffering from locally advanced ALK+ NSCLC or metastatic on targeted therapy, was it you who announced

|  |  | **Yes, systematically** | **Yes, for the majority** | **Yes, for a minority** | **No** |
| --- | --- | --- | --- | --- | --- |
| 1 | The initial diagnosis of their ALK+ NSCLC | 🞊 | 🞊 | 🞊 | 🞊 |
| 2 | Relapse/progression of the disease for your patients concerned | 🞊 | 🞊 | 🞊 | 🞊 |

**Q12.** Was the announcement of the initial diagnosis of ALK+ NSCLC in these patients the subject of

|  |  | **Yes, systematically** | **Yes, for the majority** | **Yes, for a minority** | **No** |
| --- | --- | --- | --- | --- | --- |
| 1 | A specific consultation / an announcement consultation | 🞊 | 🞊 | 🞊 | 🞊 |
| 2 | An appointment with an announcement nurse on the same day or a few days later | 🞊 | 🞊 | 🞊 | 🞊 |

**Q12B.** Has ALK+ NSCLC relapsed/progressed in your affected patients?

|  |  | **Yes, systematically** | **Yes, for the majority** | **Yes, for a minority** | **No** |
| --- | --- | --- | --- | --- | --- |
| 1 | A specific consultation / an announcement consultation | 🞊 | 🞊 | 🞊 | 🞊 |
| 2 | An appointment with an announcement nurse on the same day or a few days later | 🞊 | 🞊 | 🞊 | 🞊 |

**Q13.**Were your patients accompanied by a relative?

|  |  | **Yes, systematically** | **Yes, for the majority** | **Yes, for a minority** | **No** |
| --- | --- | --- | --- | --- | --- |
| 1 | At the time of initial diagnosis | 🞊 | 🞊 | 🞊 | 🞊 |
| 2 | At the time of disease relapse/progression | 🞊 | 🞊 | 🞊 | 🞊 |

**Q14**. Who are all the health professionals who, **for the majority of your patients,** have been involved in the announcement of the diagnosis?

|  |  | At the time of **initial diagnosis** | At the time of **disease relapse/progression** for affected patients |
| --- | --- | --- | --- |
| 1 | Oncologist | 🞎 | 🞎 |
| 2 | Pulmonologist | 🞎 | 🞎 |
| 3 | Hospital Nurse | 🞎 | 🞎 |
| 4 | Internist | 🞎 | 🞎 |
| 5 | Radiotherapist | 🞎 | 🞎 |
| 6 | Surgeon | 🞎 | 🞎 |
| 7 | General practitioner | 🞎 | 🞎 |
| 8 | Psychologist / Psychiatrist | 🞎 | 🞎 |
| 9 | Health Facilitator (ERI Meeting and Information Space) | 🞎 | 🞎 |
| 10 | Social worker | 🞎 | 🞎 |
| 11 | Radiologist | 🞎 | 🞎 |
| 12 | Others: *please specify*  …………………………………………………………… | 🞎 | 🞎 |

**Q15.** Would you say that when the diagnosis was announced and before the patient started treatment with targeted therapy, you addressed the following points...

|  |  | **Yes, systematically** | **Yes, for the majority** | **Yes, for a minority** | **No** |
| --- | --- | --- | --- | --- | --- |
| 1 | The particularities of ALK+ lung cancer | 🞊 | 🞊 | 🞊 | 🞊 |
| 2 | That this disease carries a risk of relapse | 🞊 | 🞊 | 🞊 | 🞊 |
| 3 | The reasons for choosing the treatment protocol put in place | 🞊 | 🞊 | 🞊 | 🞊 |
| 4 | How its treatment works | 🞊 | 🞊 | 🞊 | 🞊 |
| 5 | The benefits he can expect from his treatment | 🞊 | 🞊 | 🞊 | 🞊 |
| 6 | Side effects they may encounter during their treatment | 🞊 | 🞊 | 🞊 | 🞊 |
| 7 | The need to take this treatment for the long term, without stopping it (even if the patient feels better) | 🞊 | 🞊 | 🞊 | 🞊 |
| 8 | That it is a disease that is becoming chronic thanks to current treatments | 🞊 | 🞊 | 🞊 | 🞊 |

**Q15B.** Would you say that when the diagnosis was announced and before the patient started treatment with targeted therapy, the following information was understood by your patients?

|  |  | **Yes, for the majority** | **Yes, for a minority** | **No** |
| --- | --- | --- | --- | --- |
| 1 | The particularities of ALK+ lung cancer | 🞊 | 🞊 | 🞊 |
| 2 | That this disease carries a risk of relapse | 🞊 | 🞊 | 🞊 |
| 3 | The reasons for choosing the treatment protocol put in place | 🞊 | 🞊 | 🞊 |
| 4 | How its treatment works | 🞊 | 🞊 | 🞊 |
| 5 | The benefits he can expect from his treatment | 🞊 | 🞊 | 🞊 |
| 6 | Side effects they may encounter during their treatment | 🞊 | 🞊 | 🞊 |
| 7 | The need to take this treatment over the long term, without stopping it (even if the patient feels better | 🞊 | 🞊 | 🞊 |
| 8 | That it is a disease that is becoming chronic thanks to current treatments | 🞊 | 🞊 | 🞊 |

**Q15C.** When the diagnosis was announced and before the patient started treatment with targeted therapy, did you talk to him or her about other possible treatment(s) that could be put in place in the event of a relapse?

|  |  | **Yes, systematically** | **Yes, for the majority** | **Yes, for a minority** | **No** |
| --- | --- | --- | --- | --- | --- |
| 1 | At the time of initial diagnosis | 🞊 | 🞊 | 🞊 | 🞊 |
| 2 | At the time of disease relapse/progression in your affected patients | 🞊 | 🞊 | 🞊 | 🞊 |

**Q16.** What other possible treatments have you talked to your patients on targeted therapy?

|  | At the time of initial diagnosis [if ≠ not in Q15 line 1] | At the time of disease relapse/progression in your affected patients [if ≠ not in Q15 line 2] |
| --- | --- | --- |
| Chemotherapy | 🞎 | 🞎 |
| Other targeted therapies | 🞎 | 🞎 |
| Immunotherapy | 🞎 | 🞎 |
| Radiotherapy | 🞎 | 🞎 |
| Clinical Trial | 🞎 | 🞎 |

**Q17.** When the initial diagnosis or relapse is announced and before the patient starts treatment with targeted therapy, did you provide a written information material / brochure

|  |  | **Yes, systematically** | **Yes, for the majority** | **Yes, for a minority** | **No** |
| --- | --- | --- | --- | --- | --- |
| 1 | About ALK+ lung cancer | 🞊 | 🞊 | 🞊 | 🞊 |
| 2 | On the treatment protocol put in place | 🞊 | 🞊 | 🞊 | 🞊 |
| 3 | On the different supportive care that can be offered | 🞊 | 🞊 | 🞊 | 🞊 |
| 4 | On patient associations | 🞊 | 🞊 | 🞊 | 🞊 |

**Q18A. [if ≠ No online 3]** At what point in their journey do you give them a written information material / a brochure on the different supportive care offered?

| 1 | At the time of initial diagnosis most often | 🞊 |
| --- | --- | --- |
| 2 | At the time of disease relapse/progression most often | 🞊 |
| 3 | It's very variable, there isn't a moment that emerges | 🞊 |

**PART D: Follow-up of ALK+ NSCLC patients under targeted therapy [6']**

Let's talk about the follow-up of your patients with NSCLC ALK+ under targeted therapy.

**Q19.** Here are different criteria that patients can associate with each of these treatments. For each of these criteria, how would you rate each of these treatments in relation to the image your patients have of them:

*1 means that your patients do not attribute this criterion to the corresponding treatment at all. 10 that they strongly attribute this criterion to the corresponding treatment. Intermediate grades allow you to modulate your judgment*

|  |  | **Targeted therapy** | **Chemotherapy** |
| --- | --- | --- | --- |
| 1 | Quality of life preserved with this treatment | \|___\| 🞊NSP | \|___\|¤NSP |
| 2 | Favorable balance between benefits and constraints with this treatment | \|___\| 🞊NSP | \|___\|¤NSP |
| 3 | Tolerable side effects | \|___\| 🞊NSP | \|___\|¤NSP |
| 4 | Treatment that gives hope | \|___\| 🞊NSP | \|___\|¤NSP |
| 5 | Ease of administration of this treatment | \|___\| 🞊NSP | \|___\|¤NSP |
| 6 | Treatment well monitored by the medical team | \|___\| 🞊NSP | \|___\|¤NSP |
| 7 | Treatment that allows you to live almost normally | \|___\| 🞊NSP | \|___\|¤NSP |
| 8 | Treatment with long-lasting effectiveness | \|___\| 🞊NSP | \|___\|¤NSP |

**Q20.** How long do follow-up consultations last for your patients with ALK+ NSCLC on targeted therapy on average?

|___| mn on average

**Q21.** For each of the following statements, please indicate whether you agree by ticking the box that best reflects your feelings about your patients' attitudes towards ALK+ NSCLC or its treatment (targeted therapy)

| *Only one response per line* | | Strongly disagree | Rather disagree | Tend to agree | Totally agree | I don't know |
| --- | --- | --- | --- | --- | --- | --- |
| 1 | The duration of the follow-up consultations is generally perceived by my patients as sufficient | 🞊 | 🞊 | 🞊 | 🞊 | 🞊 |
| 2 | My patients don't miss any follow-up medical appointments because being monitored regularly reassures them | 🞊 | 🞊 | 🞊 | 🞊 | 🞊 |
| 3 | The time between the results of the CT scan and the consultation is often too long and a source of anxiety and stress for my patients | 🞊 | 🞊 | 🞊 | 🞊 | 🞊 |
| 4 | My patients always experience check-ups with great concern | 🞊 | 🞊 | 🞊 | 🞊 | 🞊 |
| 5 | [if yes in Q1 – line 7] The telephone support allows my patients on targeted therapy to better understand their treatment | 🞊 | 🞊 | 🞊 | 🞊 | 🞊 |
| 6 | [if yes in Q1 – line 7] Telephone support allows my patients on targeted therapy to better manage the side effects of their treatment | 🞊 | 🞊 | 🞊 | 🞊 | 🞊 |
| 7 | As soon as my patients on targeted therapy feel better, they want to stop taking their treatment | 🞊 | 🞊 | 🞊 | 🞊 | 🞊 |
| 8 | My patients sometimes feel that their targeted therapy treatment is doing them more harm than good | 🞊 | 🞊 | 🞊 | 🞊 | 🞊 |
| 9 | My patients often ask me about their life expectancy, the duration of effectiveness of their treatment | 🞊 | 🞊 | 🞊 | 🞊 | 🞊 |
| 10 | Patients with locally advanced or metastatic ALK+ NSCLC have great difficulty projecting themselves into the future | 🞊 | 🞊 | 🞊 | 🞊 | 🞊 |

**Q22.** During follow-up visits, assess yourself with your ALK+ NSCLC patients on targeted therapy for each of the following:

| *Only one response per line* | | **Yes, systematically** | **Yes, for the majority** | **Yes, for a minority** | **No** |
| --- | --- | --- | --- | --- | --- |
| 1 | The results of the **CT scan** | 🞊 | 🞊 | 🞊 | 🞊 |
| 2 | The **symptoms** of the disease: fatigue, pain, etc. | 🞊 | 🞊 | 🞊 | 🞊 |
| 3 | The possible impact of the disease/its treatment on the **professional life** of the patient or their caregiver | 🞊 | 🞊 | 🞊 | 🞊 |
| 4 | The possible repercussions of the disease/its treatment on the **patient's married and/or sexual life** | 🞊 | 🞊 | 🞊 | 🞊 |
| 5 | The **tolerance** of the treatment | 🞊 | 🞊 | 🞊 | 🞊 |
| 6 | **The effectiveness** of the treatment | 🞊 | 🞊 | 🞊 | 🞊 |
| 7 | The potential impact of the illness/treatment on the **patient's mental health** | 🞊 | 🞊 | 🞊 | 🞊 |
| 8 | The potential impact of the illness/treatment on the **mental health of the caregiver** | 🞊 | 🞊 | 🞊 | 🞊 |
| 9 | The possible impact of the disease/its treatment on **the patient's family life** | 🞊 | 🞊 | 🞊 | 🞊 |

**Q23**. Which health professionals are involved in the follow-up **for the majority of your patients** with locally advanced ALK+ NSCLC or metastatic on targeted therapy?

|  |  | **In L1** | **In L2 or higher** |
| --- | --- | --- | --- |
| 1 | Oncologist | 🞎 | 🞎 |
| 2 | Pulmonologist | 🞎 | 🞎 |
| 3 | Internist | 🞎 | 🞎 |
| 4 | General practitioner | 🞎 | 🞎 |
| 5 | Radiotherapist | 🞎 | 🞎 |
| 6 | Surgeon | 🞎 | 🞎 |
| 7 | Psychologist, psychiatrist | 🞎 | 🞎 |
| 8 | Nurses in hospitals, clinics | 🞎 | 🞎 |
| 9 | Home Nurses | 🞎 | 🞎 |
| 10 | Dietician / Nutritionist | 🞎 | 🞎 |
| 11 | Social worker | 🞎 | 🞎 |
| 12 | Physiotherapist | 🞎 | 🞎 |
| 13 | Sexologist | 🞎 | 🞎 |
| 14 | Algologist | 🞎 | 🞎 |
| 15 | Psychomotor therapist | 🞎 | 🞎 |
| 16 | Occupational therapist | 🞎 | 🞎 |
| 17 | Patient Associations | 🞎 | 🞎 |
| 18 | Life assistant | 🞎 | 🞎 |
| 19 | Esthetician/Esthetician | 🞎 | 🞎 |
| 20 | APA Instructor / Educator | 🞎 | 🞎 |
| 21 | Others: *please specify*  …………………………………………………………… | 🞎 | 🞎 |

**PART E: The impact of the disease and its management on the quality of life of patients [12 to 15']**

Now, we would like to better understand the extent to which patients with locally advanced ALK+ NSCLC + metastatic on targeted therapy are impacted in their daily lives.

**Q24**. What proportion of your patients with locally advanced or metastatic ALK+ NSCLC are on targeted therapy?

|  |  | **All my patients** | **The majority of my patients** | **A minority of my patients** | **None of my patients** | **I don't know, I don't talk about this subject with my patients** |
| --- | --- | --- | --- | --- | --- | --- |
| 1 | ... for whom the disease severely limits their daily activities | 🞊 | 🞊 | 🞊 | 🞊 | 🞊 |
| 2 | ... for whom the adverse effects of their treatment severely limit their daily activities | 🞊 | 🞊 | 🞊 | 🞊 | 🞊 |

**Q25**. [if code 1 to 3 in Q24 line 3] To what extent do these **difficulties in performing certain daily life gestures related to the disease** (moving, carrying out daily tasks, driving,...) affect the daily life of your patients affected by these difficulties?

You have scores from 1 to 10 to answer this question.

*1 means that these difficulties do not affect the daily life of the patients concerned at all. 10 that they strongly affect the daily life of your patients concerned. Intermediate grades allow you to modulate your judgment*

| ***1***  💣 | ***2***  💣 | ***3***  💣 | ***4***  💣 | ***5***  💣 | ***6***  💣 | ***7***  💣 | ***8***  💣 | ***9***  💣 | ***10***  💣 |
| --- | --- | --- | --- | --- | --- | --- | --- | --- | --- |

***Not affected at all	Is heavily affected***

**25B.** On the issue of driving, would you say that

|  | | **Yes, systematically** | **Yes, for the majority** | **Yes, for a minority** | **No** |
| --- | --- | --- | --- | --- | --- |
| 1 | ALK+ NSCLC affects patients' ability to drive | 🞊 | 🞊 | 🞊 | 🞊 |
| 2 | I recommend that patients stop driving as soon as they have brain metastases | 🞊 | 🞊 | 🞊 | 🞊 |
| 3 | I refer my patients to a licensed doctor so that he or she can carry out a medical check of fitness to drive | 🞊 | 🞊 | 🞊 | 🞊 |

**Q25C.** Do you feel comfortable with the advice to give to your patients with brain metastases on the issue of driving?

| 1 | Yes, absolutely | 🞊 |
| --- | --- | --- |
| 2 | Yes, partially | 🞊 |
| 3 | No, I lack knowledge on this subject | 🞊 |

**Please consider your patients with locally advanced ALK+ NSCLC or metastatic on targeted therapy as a professional practitioner at the time of their care pathway**.

**Q26.** What proportion of your patients are **affected by professional difficulties related to the disease** (relationship difficulties with the employer or colleagues, difficulty in meeting professional obligations, job change/loss, difficulty in progressing professionally,...)

| 1 | All my patients | 🞊 |
| --- | --- | --- |
| 2 | The majority of my patients | 🞊 |
| 3 | A minority of my patients | 🞊 |
| 4 | None of my patients | 🞊 |
| 5 | I don't know, I don't talk about this subject with my patients | 🞊 |

**Q26B**. [if code 1 to 3 in Q26] To what extent these occupational **difficulties related to the illness** (relationship difficulties with the employer or colleagues, difficulty meeting professional obligations, job change/loss, difficulty progressing professionally,...) affect the daily lives of your patients affected by these difficulties?

You have scores from 1 to 10 to answer this question.

*1 means that these difficulties do not affect the daily life of the patients concerned at all. 10 that they strongly affect the daily life of your patients concerned. Intermediate grades allow you to modulate your judgment*

| ***1***  💣 | ***2***  💣 | ***3***  💣 | ***4***  💣 | ***5***  💣 | ***6***  💣 | ***7***  💣 | ***8***  💣 | ***9***  💣 | ***10***  💣 |
| --- | --- | --- | --- | --- | --- | --- | --- | --- | --- |

***Not affected at all	Is heavily affected***

**Q26C.** If you consider your patients who are looking to return to work after a work stoppage, what proportion actually return to work?

| 1 | All my patients | 🞊 |
| --- | --- | --- |
| 2 | The majority of my patients | 🞊 |
| 3 | A minority of my patients | 🞊 |
| 4 | None of my patients | 🞊 |
| 5 | I don't know, I don't talk about this subject with my patients | 🞊 |

**Q26D.** If you consider your patients who are looking to return to work after a work stoppage, do you put them in touch with the following professionals?

|  | | **Yes, systematically** | **Yes, for the majority** | **Yes, for a minority** | **No** |
| --- | --- | --- | --- | --- | --- |
| 1 | Occupational physician | 🞊 | 🞊 | 🞊 | 🞊 |
| 2 | Social worker | 🞊 | 🞊 | 🞊 | 🞊 |

**Q26E.** Do you feel comfortable with the advice to give to your patients regarding the resumption of a professional activity?

| 1 | Yes, absolutely | 🞊 |
| --- | --- | --- |
| 2 | Yes, partially | 🞊 |
| 3 | No, I lack knowledge on this subject | 🞊 |

**Please now consider your patients with locally advanced ALK+ NSCLC or metastatic on targeted therapy as a couple at the time of their care pathway**.

**Q27**. What proportion of your patients are **affected by relationship difficulties** **related to the disease** (tensions in the life of the couple, breakup, complicated sexual relations, difficulty in investing in a relationship, etc.)

| 1 | All my patients | 🞊 |
| --- | --- | --- |
| 2 | The majority of my patients | 🞊 |
| 3 | A minority of my patients | 🞊 |
| 4 | None of my patients | 🞊 |
| 5 | I don't know, I don't talk about this subject with my patients | 🞊 |

**Q27B**. [if code 1 to 3 in Q27] To what extent are these **couple difficulties related to the disease** (tensions in the couple's life, breakup, complicated sexual relations, difficulty investing in a relationship, etc.) affect the daily lives of your patients affected by these difficulties?

You have scores from 1 to 10 to answer this question.

*1 means that these difficulties do not affect the daily life of the patients concerned at all. 10 that they strongly affect the daily life of your patients concerned. Intermediate grades allow you to modulate your judgment*

| ***1***  💣 | ***2***  💣 | ***3***  💣 | ***4***  💣 | ***5***  💣 | ***6***  💣 | ***7***  💣 | ***8***  💣 | ***9***  💣 | ***10***  💣 |
| --- | --- | --- | --- | --- | --- | --- | --- | --- | --- |

***Not affected at all	Is heavily affected***

**Q28.** What proportion of your patients with locally advanced ALK+ NSCLC or metastatic on targeted therapy **are affected by family difficulties** **related to the disease** (difficulty talking to children about the disease, asking for help, feeling of being a burden to those around them, feeling guilty about children or grandchildren, ...)

| 1 | All my patients | 🞊 |
| --- | --- | --- |
| 2 | The majority of my patients | 🞊 |
| 3 | A minority of my patients | 🞊 |
| 4 | None of my patients | 🞊 |
| 5 | I don't know, I don't talk about this subject with my patients | 🞊 |

**Q28B**. [if code 1 to 3 in Q28] To what extent do these family **difficulties related to the disease** (difficulty talking to children about the disease, asking for help, feeling of being a burden to those around them, feeling of guilt towards children or grandchildren, etc.) affect the daily lives of your patients affected by these difficulties?

You have scores from 1 to 10 to answer this question.

*1 means that these difficulties do not affect the daily life of the patients concerned at all. 10 that they strongly affect the daily life of your patients concerned. Intermediate grades allow you to modulate your judgment*

| ***1***  💣 | ***2***  💣 | ***3***  💣 | ***4***  💣 | ***5***  💣 | ***6***  💣 | ***7***  💣 | ***8***  💣 | ***9***  💣 | ***10***  💣 |
| --- | --- | --- | --- | --- | --- | --- | --- | --- | --- |

***Not affected at all	Is heavily affected***

**Q28C.** Do you have a patient in your active queue of patients with ALK+ NSCLC on targeted therapy, who has told you about a pregnancy/parenthood project?

| **1** | Yes | ○ |
| --- | --- | --- |
| **2** | No | ○ |

**Q28D.** Are you comfortable addressing this question of pregnancy / parenthood project with your patients?

| 1 | Yes, absolutely | 🞊 |
| --- | --- | --- |
| 2 | Yes, partially | 🞊 |
| 3 | No, I lack knowledge on this subject | 🞊 |

**Q28E.** Do you offer fertility preservation solutions for your patients of childbearing age?

| 1 | Yes, systematically | 🞊 |
| --- | --- | --- |
| 2 | Yes, for the majority of my patients | 🞊 |
| 3 | Yes, for a minority | 🞊 |
| 4 | No, I don't discuss this subject with my patients | 🞊 |

**Q29**. What proportion of your patients with locally advanced ALK+ NSCLC or metastatic on targeted therapy **are affected by psychological difficulties related to the disease** (anxiety, mood disorders, feelings of guilt,...)

| 1 | All my patients | 🞊 |
| --- | --- | --- |
| 2 | The majority of my patients | 🞊 |
| 3 | A minority of my patients | 🞊 |
| 4 | None of my patients | 🞊 |
| 5 | I don't know, I don't talk about this subject with my patients | 🞊 |

**Q29B**. [if code 1 to 3 in Q29] To what extent are these **psychological difficulties related to the disease** (anxiety, mood disorders, feelings of guilt,...) affect the daily lives of your patients affected by these difficulties?

You have scores from 1 to 10 to answer this question.

*1 means that these difficulties do not affect the daily life of the patients concerned at all. 10 that they strongly affect the daily life of your patients concerned. Intermediate grades allow you to modulate your judgment*

| ***1***  💣 | ***2***  💣 | ***3***  💣 | ***4***  💣 | ***5***  💣 | ***6***  💣 | ***7***  💣 | ***8***  💣 | ***9***  💣 | ***10***  💣 |
| --- | --- | --- | --- | --- | --- | --- | --- | --- | --- |

***Not affected at all	Is heavily affected***

**Q30**. What proportion of your patients with locally advanced ALK+ NSCLC or metastatic on targeted therapy **are affected by financial difficulties related to the disease** (reduced income, difficulties in obtaining a loan, insufficient social assistance,...)

| 1 | All my patients | 🞊 |
| --- | --- | --- |
| 2 | The majority of my patients | 🞊 |
| 3 | A minority of my patients | 🞊 |
| 4 | None of my patients | 🞊 |
| 5 | I don't know, I don't talk about this subject with my patients | 🞊 |

**Q30B**. [if code 1 to 3 in Q30] To what extent are these **financial difficulties related to the disease** (drop in income, difficulty obtaining a loan, insufficient social assistance,...) affect the daily lives of your patients affected by these difficulties?

You have scores from 1 to 10 to answer this question.

*1 means that these difficulties do not affect the daily life of the patients concerned at all. 10 that they strongly affect the daily life of your patients concerned. Intermediate grades allow you to modulate your judgment*

| ***1***  💣 | ***2***  💣 | ***3***  💣 | ***4***  💣 | ***5***  💣 | ***6***  💣 | ***7***  💣 | ***8***  💣 | ***9***  💣 | ***10***  💣 |
| --- | --- | --- | --- | --- | --- | --- | --- | --- | --- |

***Not affected at all	Is heavily affected***

**Q31**. What proportion of your patients with locally advanced ALK+ NSCLC or metastatic on targeted therapy **are affected by difficulties in projecting themselves into the future** (giving up certain projects/activities/trips)

| 1 | All my patients | 🞊 |
| --- | --- | --- |
| 2 | The majority of my patients | 🞊 |
| 3 | A minority of my patients | 🞊 |
| 4 | None of my patients | 🞊 |
| 5 | I don't know, I don't talk about this subject with my patients | 🞊 |

**Q31B**. [if code 1 to 3 in Q31] To what extent do these **difficulties of projecting into the future** (renunciation of certain projects/activities/travel) affect the daily life of your patients affected by these difficulties?

You have scores from 1 to 10 to answer this question.

*1 means that these difficulties do not affect the daily life of the patients concerned at all. 10 that they strongly affect the daily life of your patients concerned. Intermediate grades allow you to modulate your judgment*

| ***1***  💣 | ***2***  💣 | ***3***  💣 | ***4***  💣 | ***5***  💣 | ***6***  💣 | ***7***  💣 | ***8***  💣 | ***9***  💣 | ***10***  💣 |
| --- | --- | --- | --- | --- | --- | --- | --- | --- | --- |

***Not affected at all	Is heavily affected***

**Q31C.** In summary, among all these difficulties caused by the disease, please select the 3 that in your opinion most affect the daily life of the majority of your patients ?

Please rank in hierarchical order and rate 1 for the difficulty that affects their daily life the most, then 2 for the difficulty you rank in 2nd position, then 3 for the third

| 1 | Physical difficulties in performing daily life | /____/ |
| --- | --- | --- |
| 2 | Professional difficulties | /____/ |
| 3 | Difficulties in married life, intimate life | /____/ |
| 4 | Difficulties in family life | /____/ |
| 6 | Psychological difficulties | /____/ |
| 7 | Financial hardship | /____/ |
| 8 | Difficulties in projecting oneself into the future | /____/ |

**Q32.** Finally, here are some different actions/initiatives that could be put in place to support patients with ALK+ cancer on targeted therapy. How important do you think they are to put in place?

**Please give a score from 1 to 10.**

*1 = action not at all important to put in place according to you*

*10 = very important action to put in place according to you*

*Intermediate grades allow you to modulate your judgment*

| 1 | To make it easier for patients to report the side effects of their treatment to the medical team that follows them | /____/ |
| --- | --- | --- |
| 2 | More regular assessments by the medical team of their supportive care needs | /____/ |
| 3 | That patients can benefit from supportive care via external structures that would be closer to their home | /____/ |
| 4 | Tools to work on their memory, concentration, attention | /____/ |
| 5 | Give them more systematic access to activities for their well-being (yoga, meditation, sophrology, shiatsu, adapted physical activity, etc.) | /____/ |
| 6 | Encourage their exchanges with other patients to break the isolation in the face of the disease through local support groups | /____/ |
| 7 | Giving patients access to return-to-work information | /____/ |
| 8 | Offer psychological support in a more systematic way to enable them to live better with the disease | /____/ |
| 9 | Set up a "Quality of Life" consultation for patients and their caregivers in HDJ which would be dedicated to the non-medical difficulties encountered (social, family, professional,...) | /____/ |

**This questionnaire is now closed, thank you for your participation!**
